# Non-pharmaceutical interventions, vaccination, and the SARS-CoV-2 delta variant in England: a mathematical modelling study

**DOI:** 10.1016/S0140-6736(21)02276-5

**Published:** 2021-11-13

**Authors:** Raphael Sonabend, Lilith K Whittles, Natsuko Imai, Pablo N Perez-Guzman, Edward S Knock, Thomas Rawson, Katy A M Gaythorpe, Bimandra A Djaafara, Wes Hinsley, Richard G FitzJohn, John A Lees, Divya Thekke Kanapram, Erik M Volz, Azra C Ghani, Neil M Ferguson, Marc Baguelin, Anne Cori

**Affiliations:** a MRC Centre for Global Infectious Disease Analysis, Jameel Institute, School of Public Health, Imperial College London, London, UK; b National Institute for Health Research Health Protection Research Unit in Modelling Methodology, Imperial College London, Public Health England, London School of Hygiene & Tropical Medicine, London, UK; c Modelling and Economics Unit, National Infection Service, Public Health England, London, UK; d Department of Infectious Disease Epidemiology, Faculty of Epidemiology and Population Health, London School of Hygiene & Tropical Medicine, London, UK

## Abstract

**Background:**

England's COVID-19 roadmap out of lockdown policy set out the timeline and conditions for the stepwise lifting of non-pharmaceutical interventions (NPIs) as vaccination roll-out continued, with step one starting on March 8, 2021. In this study, we assess the roadmap, the impact of the delta (B.1.617.2) variant of SARS-CoV-2, and potential future epidemic trajectories.

**Methods:**

This mathematical modelling study was done to assess the UK Government's four-step process to easing lockdown restrictions in England, UK. We extended a previously described model of SARS-CoV-2 transmission to incorporate vaccination and multi-strain dynamics to explicitly capture the emergence of the delta variant. We calibrated the model to English surveillance data, including hospital admissions, hospital occupancy, seroprevalence data, and population-level PCR testing data using a Bayesian evidence synthesis framework, then modelled the potential trajectory of the epidemic for a range of different schedules for relaxing NPIs. We estimated the resulting number of daily infections and hospital admissions, and daily and cumulative deaths. Three scenarios spanning a range of optimistic to pessimistic vaccine effectiveness, waning natural immunity, and cross-protection from previous infections were investigated. We also considered three levels of mixing after the lifting of restrictions.

**Findings:**

The roadmap policy was successful in offsetting the increased transmission resulting from lifting NPIs starting on March 8, 2021, with increasing population immunity through vaccination. However, because of the emergence of the delta variant, with an estimated transmission advantage of 76% (95% credible interval [95% CrI] 69–83) over alpha, fully lifting NPIs on June 21, 2021, as originally planned might have led to 3900 (95% CrI 1500–5700) peak daily hospital admissions under our central parameter scenario. Delaying until July 19, 2021, reduced peak hospital admissions by three fold to 1400 (95% CrI 700–1700) per day. There was substantial uncertainty in the epidemic trajectory, with particular sensitivity to the transmissibility of delta, level of mixing, and estimates of vaccine effectiveness.

**Interpretation:**

Our findings show that the risk of a large wave of COVID-19 hospital admissions resulting from lifting NPIs can be substantially mitigated if the timing of NPI relaxation is carefully balanced against vaccination coverage. However, with the delta variant, it might not be possible to fully lift NPIs without a third wave of hospital admissions and deaths, even if vaccination coverage is high. Variants of concern, their transmissibility, vaccine uptake, and vaccine effectiveness must be carefully monitored as countries relax pandemic control measures.

**Funding:**

National Institute for Health Research, UK Medical Research Council, Wellcome Trust, and UK Foreign, Commonwealth and Development Office.

## Introduction

Despite the UK being the first country to start nationwide vaccination campaigns,^1^ the emergence of the alpha (B.1.1.7) variant of concern drove the severe second wave over the 2020–21 winter leading to a third lockdown in England from Jan 5, 2021.^2^ Informed by mathematical modelling, the UK Government published a roadmap out of lockdown policy for England, setting out the conditions for and expected timeline of a stepwise lifting of non-pharmaceutical interventions (NPIs).^3^ Between March 8 and July 19, 2021, NPIs were incrementally lifted as vaccination coverage increased. By July 19, 87·5% of the adult population in England had received at least one dose of vaccine, and 68·2% had received two doses.^1^ The impact of each roadmap step was assessed in real time before further interventions were lifted, against the Government's four tests as follows: continued success of the vaccine programme; evidence of the effectiveness of vaccines against hospitalisation; no risk of overwhelming the National Health Service (NHS); and new variants of concern do not change the risk assessment.^3^

**Research in context**

**Evidence before this study**

We searched PubMed up to July 23, 2021, with no language restrictions using the following search terms: (COVID-19 or SARS-CoV-2 or 2019-nCoV or “novel coronavirus”) AND (vaccine or vaccination) AND (“non pharmaceutical interventions” OR “non-pharmaceutical interventions”) AND (model*). We found nine studies that analysed the relaxation of controls with vaccination roll-out. However, none explicitly analysed real-world evidence, balancing lifting of interventions, vaccination, and emergence of the delta variant.

**Added value of this study**

Our data synthesis approach combines real-world evidence from multiple data sources to retrospectively assess how relaxation of COVID-19 measures have been balanced with vaccination roll-out. We explicitly capture the emergence of the delta variant, its transmissibility over alpha, and quantify its impact on the roadmap. We show the benefits of maintaining non-pharmaceutical interventions while vaccine coverage continues to increase and capture key uncertainties in the epidemic trajectory after NPIs are lifted.

**Implications of all the available evidence**

Our study shows that lifting interventions must be balanced carefully and cautiously with vaccine roll-out. In the presence of a new, highly transmissible variant, vaccination alone might not be enough to control COVID-19. Careful monitoring of vaccine uptake, effectiveness, variants, and changes in contact patterns as restrictions are lifted will be crucial in any exit strategy.

The emergence of variants of concern, notably the lineages alpha, beta (B.1.351), gamma (P.1), and delta (B.1.617.2) first detected in the UK,^4^ South Africa,^5^ Brazil,^6^ and India,^7^ respectively, has posed recurring challenges for pandemic control efforts globally. The delta variant was designated a variant of concern on May 6, 2021, by Public Health England (PHE) and quickly became the dominant variant in the UK.^8^ Delta is substantially more transmissible than alpha,^8^, ^9^ a variant already 50–80% more transmissible than previously circulating variants,^4^, ^10^ and it is estimated to have a 1·85–2·6-fold increase in the risk of hospital admission.^11^, ^12^ It is also associated with partial immune escape and consequent reductions in vaccine effectiveness and cross-protection from previous non-delta infections.^13^, ^14^

Delta's emergence in the UK in mid-April, 2021,^15^ drove a rapid increase in cases and hospital admissions across all areas of England,^8^ prompting the final roadmap step (step four) to be delayed by a month to July 19.^16^ Daily case numbers in England started increasing from mid-May, 2021, and grew exponentially from mid-June to mid-July, reaching a peak of 56 282 on July 15. Numbers then unexpectedly and synchronously fell to half that value over the following 2 weeks, before plateauing and beginning to rise slowly again in August.^17^

In this study, we quantify the impact of each of the four steps of the roadmap—school reopening; outdoor hospitality and non-essential retail reopening; indoor hospitality reopening; and lifting of all remaining restrictions.^3^ Our model framework was developed and adapted throughout the COVID-19 epidemic to provide quantitative evidence and epidemiological insights to the UK Government. As an update to this work, we show what the impact of the full roadmap would have been in the absence of delta, and summarise the real-time modelling of policy options that informed the delaying of the final step four.^18^, ^19^, ^20^ We also assess the potential epidemic magnitude, timing, and main sources of uncertainty after step four under different vaccine effectiveness and immune escape assumptions of delta and the level of transmissibility after NPIs are lifted,^21^ taking into account recent trends in case incidence and hospital admissions.

## Methods

### Study design

This epidemiological mathematical modelling study was done to assess the UK Government's four-step process to easing lockdown restrictions in England, UK.

Ethics permission was sought for the study via Imperial College London's (London, UK) standard ethical review processes and was approved by the College's Research Governance and Integrity Team (ICREC reference 21IC6945).

### Epidemiological model and fitting

We extended a previously described stochastic SARS-CoV-2 transmission model to include vaccination and to capture multiple variants.^22^ We used Bayesian methods to fit a single strain transmission model (capturing alpha and pre-alpha variants, referred to as alpha hereafter) to multiple data sources, including daily deaths, hospital admissions and bed occupancy, serological data, and population-level PCR tests, from each English NHS region up to March 8, 2021. By fitting a piecewise linear time-varying transmission rate with change points aligned to policy change dates, the model reliably captures the age-specific scale and timing of the first two waves in England.^22^ To model vaccine roll-out, we assumed an 11-week interval between first and second vaccine doses with the distribution of doses by vaccine type and uptake by age informed by detailed NHS data on vaccine administration. To explicitly model the emergence of the delta variant, we then fitted a two-strain version of the same model to data from March 8 to July 31, 2021, using information propagated from the first inference step and additionally fitting to the PHE variant and mutation dataset. The variant and mutation dataset lists all genotyped or sequenced SARS-CoV-2 cases in England by region and date of specimen. By fitting to the frequency of delta among alpha and delta cases over time, we were able to estimate the transmission advantage of delta over alpha.

We then used the fitted two-strain model to project the epidemic trajectory after July 19 under different scenarios. For these forward projections, we accounted for the effect of school holidays on transmission (except July 23–Sept 1, which overlaps with the period directly after step four where we assume a gradual increase in contacts) and seasonality in SARS-CoV-2 transmission (for full model description and data sources see appendix pp 3–6).

### Characteristics of the delta variant

In our model fitting and forward projections (note that in our forward projections, our central projected trend is the median across all daily projections and is not a single trajectory), we explored plausible ranges of key epidemiological characteristics of delta. First, we used estimates of the transmission advantage of delta over alpha, which were informed by fitting the two-strain model to variant and mutation data. Second, we allowed for imperfect cross-immunity between alpha and delta: infection with a pre-delta variant (eg, alpha) provides only 75–100% protection against infection with delta (appendix p 7). This cross-protection was modelled independently from vaccine effectiveness. Third, we explored central, optimistic, and pessimistic assumptions for vaccine effectiveness against delta, including protection against death, severe disease, mild disease or infection, and onward infectiousness, informed by recent studies (table).^12^, ^28^, ^34^ Fourth, we assumed that vaccine-induced and infection-induced immunity were independent, with vaccines inducing long-lasting immunity. We examined varying assumptions about the duration of infection-induced immunity: lifelong or average of 6-year or 3-year duration (appendix pp 38, 42).^35^, ^36^, ^37^ Furthermore, we accounted for the increased severity of delta relative to alpha by assuming a 1·85-fold increased risk of hospital admission.^12^

**Table:** Vaccine effectiveness assumptions for three two-dose vaccines licensed for use in England

	**Alpha**	**Delta (central)**	**Delta (optimistic)**	**Delta (pessimistic)**
**Effectiveness against death**				
AZ (one dose)^23^, ^24^	80%	80%	80%	75%
AZ (two doses)^23^, ^24^, ^25^	95%	95%	95%	95%
PF (one dose)^23^, ^24^	85%	85%	85%	80%
PF (two doses)^23^, ^24^	95%	95%	95%	95%
**Effectiveness against severe disease**				
AZ (one dose)^26^, ^27^	80%	80%	80%	75%
AZ (two doses)^26^, ^27^, ^28^*	90%	90%	90%	85%
PF (one dose)^29^	85%	85%	85%	80%
PF (two doses)^28^, ^30^*	95%	95%	95%	90%
**Effectiveness against mild disease or infection**†				
AZ (one dose)^12^, ^28^, ^31^	50%	33%	45%	20%
AZ (two doses)^12^, ^13^, ^31^, ^32^	74%	58%	70%	45%
PF (one dose)^12^, ^13^, ^27^, ^30^	50%	33%	45%	20%
PF (two doses)^12^, ^13^, ^30^, ^33^	93%	85%	90%	78%
**Effectiveness against infectiousness if infected**				
All vaccines (one and two doses)^23^	45%	40%	45%	35%

We assumed that MD had the same vaccine effectiveness as PF for first and second doses. AZ=Oxford–AstraZeneca ChadOx1 nCov-19 AZD1222. MD=Moderna mRNA-1273. PF=Pfizer–BioNTech COVID-19 vaccine BNT162b2.

† Vaccine effectiveness against infection was assumed equal to vaccine effectiveness against mild disease.

* Assumed greater than mild disease.

### Assessing the impact of delta on the roadmap

We explored counterfactual scenarios of the impact of the roadmap on the epidemic trajectory in the presence and absence of delta. We compared the projected number of infections, hospital admissions, and deaths for scenarios with and without delta, varying cross-protection, vaccine effectiveness, and waning immunity as described above. To capture the easing of restrictions at step four, we sampled from a range of values for the reproduction number *R,* the average number of secondary infections generated by one case, in the absence of naturally induced and vaccine-induced immunity (*R*_excl_immunity_) that could occur at that stage. We also estimated, for each scenario, the resulting effective reproduction number (*R*_t_^eff^), which accounts for naturally induced and vaccine-induced immunity (appendix p 36). Our baseline scenario assumes contacts increase gradually over an 11-week period after step four to a maximum of 40% (low mixing), 70% (moderate mixing), or 100% (high mixing) greater than contact rates estimated for the period that step three was in place (average *R*_excl_immunity_; appendix p 43),^38^ central vaccine effectiveness and cross-immunity (appendix pp 9, 42), and a 3-year average duration of infection-induced immunity. We also assessed the impact of delaying step four, planned initially for not before June 21, but delayed to July 19.

### Sensitivity analysis

To understand the main drivers of uncertainty of the magnitude of the third wave, in the forward projections we systematically varied four factors as follows: vaccine effectiveness against delta; cross-protection against delta from previous infection with non-delta variants; the duration of natural immunity; and the level of transmissibility after step four (appendix pp 47–48). Shapley values are used to quantify the relative importance of each parameter (appendix p 71). We explored the impact of waning vaccine-induced protection in a separate sensitivity analysis (appendix pp 12, 76). We also assessed a further counterfactual scenario in which contacts increased to maximum levels immediately after the step four date, rather than gradually as in the baseline scenario.

### Role of the funding source

The funders of this study had no role in study design, data collection, data analysis, data interpretation, or writing of the report.

## Results

**Figure 1:**
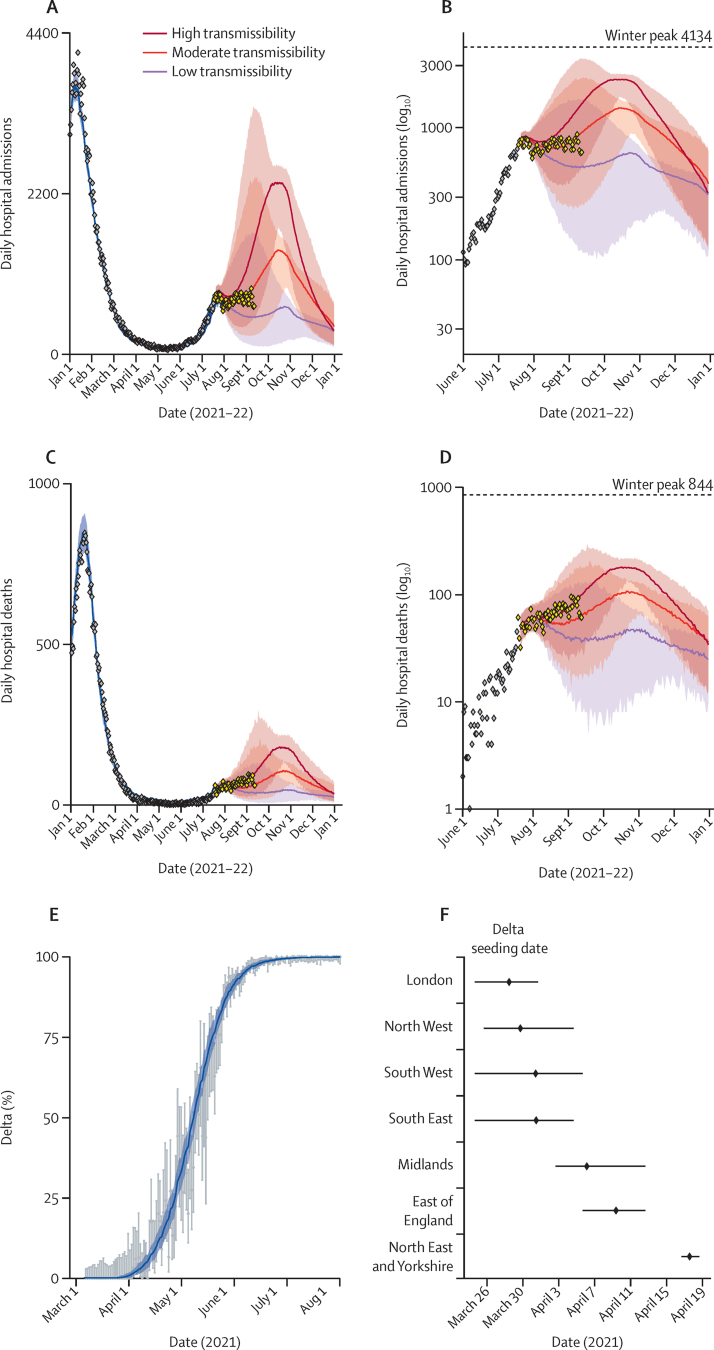
Trajectory of the COVID-19 epidemic in England and the emergence of the delta variant Observed (grey points) daily hospital admissions (A), log hospital admissions (B), hospital deaths (C), and log hospital deaths (D). The blue line shows the model fit up to July 19, 2021, and the red and purple lines show the projected admissions and deaths assuming a central vaccine effectiveness, cross-immunity, and immunity duration and a gradual increase in contacts and a return to high (dark red), moderate (light red), or low (purple) transmissibility (contact rates) after non-pharmaceutical interventions are lifted. The shaded area is the 95% credible interval. Note that the central projected trend is the median across all daily projections and is not a single trajectory. The yellow points in panels A–D show the most recent data (July 19–Sept 16, 2021), which were not fitted to. (E) Model fit to the daily proportion of cases due to the delta variant (variant and mutation data) over time from March 8–July 19, 2021, in London as an example (appendix p 54 shows other regions). Points show the data, bars are 95% CIs, the blue line is the model fit, and the shaded area is the 95% credible interval. (F) Estimated delta seeding date by UK National Health Service region. Points show the median estimate and horizontal bars show the 95% credible interval.

The model effectively reproduces national (figure 1) and regional (appendix p 49) trends in the SARS-CoV-2 epidemic in England between Dec 1, 2020, and July 19, 2021, including the July dip in case numbers, hospital admissions (figure 1A, B), and hospital deaths (figure 1C, D), and the emergence and eventual dominance of the delta variant (figure 1E, F). In our model validation (figure A–D), we show that our original model projections match the subsequently observed hospital admissions and deaths between July 19 and Sept 16, 2021.

**Figure 2:**
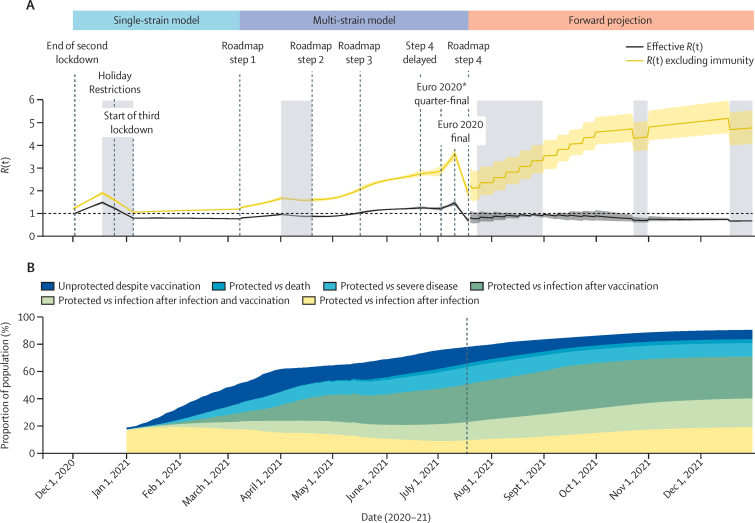
Prevalence-weighted effective *R*(t) and *R*(t) excluding infection-induced or vaccine-induced immunity (A) and proportion of the population in England protected after infection or vaccination against infection, severe disease, or death (B), over time (A) Estimated values from the end of the second national lockdown up to July 19, 2021, and assumed values thereafter. The solid line shows the median R(t) and the shaded area shows the 95% credible interval. Shaded area shows school holidays—note we do not explicitly model the impact of school closures for the period July 23–Aug 31 in order to capture the overall gradual increase in contacts from July 19 to Oct 1 (appendix p 44). The forward projection section of the figure corresponds to the central immunity and gradual return to moderate mixing scenario. Note that our central projected trend is the median across all daily projections and is not a single trajectory. (B) Proportion of the English population, from Jan 1, 2021, protected after infection or vaccination over time against infection, severe disease, or death. The vertical dashed line shows the separation between the observed vaccination schedule up to July 31, followed by the simulated schedule assuming central immunity and a gradual return to moderate transmissibility after non-pharmaceutical restrictions are lifted. The white space in the plot corresponds to individuals who have neither been vaccinated against nor infected with SARS-CoV-2. *R*(t)=reproduction number. *Euro 2020 football tournament.

Figure 2 shows estimates of how the reproduction number and population immunity, under our central immunity and moderate transmissibility scenario, changed over time since December, 2020. Overall, while *R*_excl_immunity_ increased markedly from March to July, 2021, as a result of the relaxation of lockdown and then the emergence of the delta variant, the rapid roll-out of vaccines progressively increases the gap between *R*_excl_immunity_ and *R*_t_^eff^. The Christmas school holidays and accompanying near-lockdown physical distancing in December, 2020, followed by the third national lockdown in January, 2021, successfully brought *R*_t_^eff^ below the critical threshold of one. This period coincided with a rapid expansion of the national vaccination programme. By March 8 (step one of the roadmap) when educational institutions reopened, 43% of eligible adults (>18 years) had received their first vaccine dose and 2% had their second dose.^1^ Cases, hospital admissions, and deaths continued to decrease and remained at low levels even after schools reopened (figure 1A, C). Although we estimated a slight increase in *R*_t_^eff^ after step one, Easter holidays (from April 1, 2021) and the roll-out of vaccination maintained *R*_t_^eff^ below one (figure 2A) when non-essential retail opened (61% first dose and 15% second dose coverage by April 12, step two).^1^ The delta variant, detected in early April, 2021, predominantly in London and the North West NHS regions (figure 1F), was designated as a variant under investigation on April 15, after increasing numbers of locally acquired infections with that variant were detected (figure 1E).^15^

We estimated that the *R*_t_^eff^ for alpha remained less than one (appendix p 54) after step two because of increasing population immunity from vaccination, with uptake increasing to 69% for first dose and 39% for second dose by May 17 (step three, resumption of indoor hospitality and inter-household mixing^1^). However, after initial seeding between late March and early April (figure 1F) the proportion of delta variant cases increased rapidly across all regions in this period (figure 1E; appendix p 54). This rapidly drove *R*_t_^eff^ to greater than one by mid-May, reflecting the 76% (95% credible interval [CrI] 69–83) transmission advantage of delta compared with alpha that we estimate (figure 2A).

The increase in contacts after step three continued to be offset by the increasing vaccine-induced population immunity (figure 2); 82% had a first dose and 60% had a second dose by June 21).^1^ However, the net impact of infection-induced and vaccine-induced immunity differed qualitatively by variant. We estimated that *R*_t_^eff^ for alpha remained less than one through to mid-July, whereas *R*_t_^eff^ for delta remained greater than one (appendix p 54).

A further sharp increase in *R*_t_^eff^ was then seen in the first half of July. At the time, we and other modelling groups advising the UK Government concluded that the increase was a belated result of step three, but the rapid drop after July 11 (and other data indicating a sex imbalance in case incidence),^39^ suggest that the increase in transmission rates seen in that period resulted from a transient increase in population contact rates, particularly in young men, probably associated with the Euro 2020 football tournament. At the time of writing, that decrease has reversed, with a gradual increase in case incidence in August.^17^

Despite high vaccine coverage in adults, sufficient population susceptibility remains for a third wave to occur as contact rates rise (figure 2). The proportion protected against a delta infection after vaccination is substantially lower than for alpha. Additionally, most individuals younger than 18 years have neither been vaccinated nor infected. In this context, delta's high transmissibility means that population immunity, whether vaccine induced or infection induced, is insufficient to keep *R*_t_^eff^ below one.

**Figure 3:**
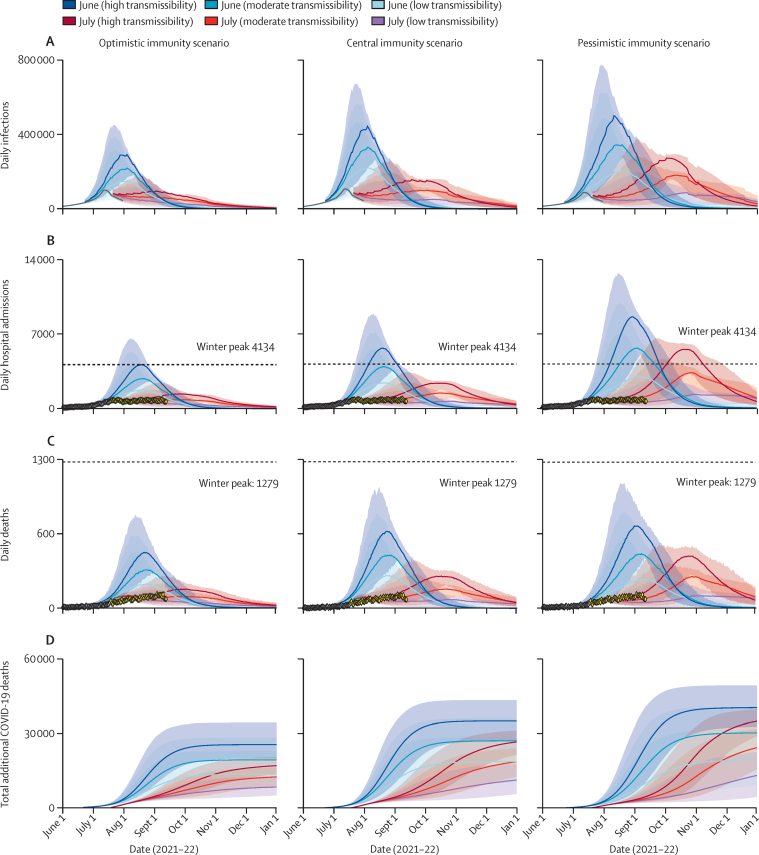
England COVID-19 daily infections (A), hospital admissions (B), deaths (C), and total additional deaths between June 21, 2021, and Jan 1, 2022 (D) Assumptions were that all remaining NPIs were lifted on June 21 (blue) or July 19 (red and purple) with a gradual increase in contacts over 11 weeks thereafter, and a return to low (light blue or purple), moderate (medium blue or red), or high (dark blue or red) transmissibility (contact rates). The grey points show the fitted data and yellow the most recent trends (July 19–Sept 16, not fitted). Each column shows projections assuming delta variant with optimistic (left column); central (middle column); and pessimistic (right column) vaccine effectiveness, cross-protection, and waning immunity assumptions (appendix pp 9, 38). The plots are truncated on Jan 1, 2022, but model results in the main text are based on simulations up to June 1, 2022. The central projected trend is the median across all daily projections and is not a single trajectory. Shading shows 95% credible intervals.

With the emergence of delta, our projections show that had step four occurred on June 21, as initially planned, it might have caused a substantial third wave of hospital admissions and deaths, but with wide uncertainty regarding the magnitude and trajectory of that wave (figure 3). Projected total deaths between June 21, 2021, and June 1, 2022, ranged from 13 400 (95% CrI 8300–22 700) in the most optimistic scenario (high vaccine effectiveness, high cross-protection, slower waning of natural immunity, and 40% increase in contacts) to 40 600 (33 000–49 500) in the most pessimistic scenario (low vaccine effectiveness, low cross-protection, faster waning of natural immunity, and 100% increase in contacts; appendix pp 9, 38, 43).

We found that delaying step four until July 19, was beneficial for all scenarios considered, with a much smaller projected wave, although still highly uncertain and sensitive to assumptions around vaccine effectiveness against delta and mixing after NPIs are lifted (figure 3). The delay allowed the distribution of an additional 2·8 million first doses and 3·8 million second doses between June 21 and July 19,^1^ reducing the projected future peak of daily hospital admissions by three fold from 3900 (95% CrI 1500–5700) to 1400 (700–1700) in the baseline scenario of central immunity and moderate mixing (figure 3B). It also reduced the total predicted deaths between June 21, 2021, and June 1, 2022, by about 20% (appendix pp 62–70). Had contact rates increased more abruptly after step four, the reductions in peak hospital admissions and deaths caused by delaying until July 19, would have been substantially greater (see appendix p 60). However, trends in cases, hospital admissions, and death data in the months up to mid-August, 2021, suggest that contacts have increased gradually since step four, in line with the assumptions of our baseline scenario (figure 1B, D).

In sensitivity analyses, we found that the level of mixing of people after NPIs are lifted was the main driver of uncertainty of the magnitude of the third wave, followed by vaccine effectiveness, the level of cross-immunity, and waning immunity. The rate at which previous infection-induced immunity waned had a greater impact on expected total infections but only minimally impacted expected hospital admissions or deaths (appendix pp 72–73). As expected, allowing for waning of vaccine-induced immunity resulted in a higher number of hospital admissions and deaths. However, this sensitivity analysis did not include the potential mitigating impact of planned booster doses (appendix pp 12, 76).

We project future deaths predominantly in fully vaccinated individuals aged 75 years or older because of the high vaccine uptake of a highly effective, but not 100% effective, vaccine in the most vulnerable age groups; it does not imply a poor vaccine effectiveness against death. We also anticipate a substantial number of deaths among fully vaccinated adults aged 50–74 years (appendix p 61).

## Discussion

Mathematical models are valuable tools to inform the design, monitoring, and evaluation of vaccination programmes.^40^ In this study, we retrospectively assessed steps one to three of England's roadmap out of lockdown policy, and prospectively explored the impact of step four. By extending our evidence synthesis framework to account for vaccinations and multiple variants, we reliably captured the past epidemic and quantified the impact of each phase of lifting interventions and the emergence of the delta variant on transmission.^22^

We show that the roadmap was successful in mitigating the increase in mixing due to lifting NPIs, by increasing population-level immunity through the mass vaccination programme. Our projections show that in the absence of the delta variant, lifting NPIs at the planned earliest date of the final step (June 21) would not have resulted in a substantial third wave. This emphasises the importance of carefully aligning the lifting of interventions with immunity levels in the population. Israel rapidly vaccinated a high proportion of their population throughout their reopening plan, and then implemented vaccination passes for entry to high-risk settings.^41^, ^42^ Conversely, case incidence and hospital admissions are currently surging in several US states, due to restrictions being relaxed when vaccination coverage was too low to give sufficient population immunity.^43^

Our analyses emphasise the significant impact that the emergence of delta had on the planned roadmap. Similar to previous studies, we estimated that delta has an average 76% (95% CrI 69–83) transmissibility advantage over alpha.^9^ Together with reductions in vaccine effectiveness for delta and waning of natural immunity, this drove a rapid increase in cases and hospital admissions from mid-May that was not offset by the vaccination programme.^28^, ^34^ A similar displacement of alpha and a surge in cases was observed in India where delta was first detected.^44^ The dominance of delta has now led to a tightening of restrictions in many countries, including in Israel, the country with the fastest vaccination roll-out.^45^

Across all the scenarios we examined, delaying step four until July 19 was beneficial because it allowed more individuals to be vaccinated. In the baseline scenario, this delayed and reduced the peak of hospital admissions by three fold and reduced total deaths between June, 2021 and June, 2022 by about 20%.

We projected that a substantial autumn wave of transmission is possible, at least in the absence of substantial additional vaccination (eg, booster doses and full vaccination of teenagers), but with large uncertainty around the resulting peak number of hospital admissions and total deaths. This uncertainty is driven by uncertainty around levels of mixing after NPIs are lifted and by imperfect knowledge of vaccine effectiveness against delta. Transmission intensity (*R*_t_^eff^) in the coming months will depend on how high and how quickly population contact rates will increase, the continued use of face coverings,^46^ physical distancing, and adherence to case isolation.^47^, ^48^

The rapid exponential growth in case incidence in the first half of July, 2021, illustrates the high transmission levels that could be reached if contact rates approach prepandemic levels in the coming months. Fortunately, that increase in contact rates, probably caused by the Euro 2020 football tournament,^39^ proved transient, and was then followed by the synchronous self-isolation of contacts alerted through NHS test and trace^49^ and the start of school holidays a week later, driving transmission down for the last 2 weeks of July. Polls showed that 57% of UK adults were worried about the removal of legal restrictions and 66% would continue to wear a face covering after July 19.^50^ The average number of contacts in early September, 2021, was still much lower than prepandemic levels,^51^ and our baseline scenario, which assumes a gradual increase in contact rates, most closely reflects current trends. We estimate that this slow increase in contact rates after July 19 will reduce the peak number of hospital admissions and total deaths compared with an abrupt increase.

Uncertainty around vaccine effectiveness means it is difficult to accurately estimate the overall level of population immunity accounting for waning and imperfect cross-protection. At high vaccine coverage, even a difference of 98% versus 95% in vaccine effectiveness against mortality translates to a doubling of projected deaths.^52^ The duration of infection-induced and vaccine-induced immunity for all SARS-CoV-2 lineages remains another key unknown that will determine long-term transmission dynamics. We explored the impact of waning vaccine-induced protection in a simple sensitivity analysis. Including waning of vaccine protection had no impact on our retrospective assessment of steps one to three of the roadmap, but resulted in a substantially higher number of projected hospital admissions and deaths. Better characterising the duration of natural and vaccine-induced protection against infection and severe disease will be important for informing booster vaccination programmes. We have focused on deaths and hospital admissions as primary outcomes in our analysis, given the impact of hospital admissions on NHS capacity. However, an estimated 1·5% of individuals in the UK had symptoms of so-called long COVID (symptoms lasting >4 weeks) on July 4, 2021.^53^ Although our estimates of infections and cases over time might capture this wider burden of disease,^54^ we did not explicitly quantify this. Overall infection levels also determine the risks of new variants of concern emerging within the UK with the risk increasing with transmission levels.

Our analysis has a number of limitations. We did not consider reintroduction of NPIs, vaccination of people younger than 18 years, nor booster doses in our projections. These measures might at least partially mitigate the third wave. A first dose of vaccine has now been advised for all eligible 12–17-year-olds and booster doses for individuals aged 50 years or older is being rolled out.^55^, ^56^ However, given the age profile of projected hospital admissions and deaths, we anticipate expansion of vaccine eligibility to children might only have a moderate effect. Although we modelled heterogeneity by age in mixing patterns and capture changes in the overall level of mixing over time, we assumed that age-related mixing patterns remained constant over time. We also modelled age-dependent vaccine uptake, which we assumed was independent of mixing patterns or viral transmission, but did not explicitly model other types of heterogeneity (eg, by occupation, sociodemographic, and ethnic groups^57^, ^58^) which might affect both the risk of infection and vaccine uptake. Groups of individuals who are both at high risk of infection and less likely to take the vaccine might lead to continued outbreaks among vulnerable populations and reduce the overall impact of vaccination. Furthermore, our analysis focused only on outcomes directly related to COVID-19: we did not consider the impact on health services, other diseases, mental health, or the economic impact of measures.

In summary, our study shows how the phased lifting of NPIs in England, coordinated with vaccine roll-out, has been largely successful at keeping hospital admissions and deaths at low levels since March, 2021. However, our projections show that the high transmissibility of delta, imperfect vaccine effectiveness, and future increases in contact rates are likely to lead to a substantial wave of transmission in the autumn, albeit of highly uncertain magnitude. Overall, our analysis highlights the clear benefit of early and accessible national vaccination programmes that allow population immunity to increase to high levels before NPIs are lifted. Furthermore, we have shown that vaccination alone in the absence of NPIs might not be sufficient to control delta, even with high vaccination coverage. We quantified how the emergence of delta affected the progress of the roadmap and the benefit of delaying step four of that roadmap by 1 month. The experience of delta highlights the threat posed by any future variants of concern and underscores the need for global collaborative efforts to control transmission and mitigate the risk of emergence of new variants of concern through equitable global access to vaccines.

## Data sharing

All data files and source code required to reproduce this analysis are publicly available at GitHub.

## Declaration of interests

AC has received payment from Pfizer for teaching of mathematical modelling of infectious diseases. KAMG has received honoraria from Wellcome Genome Campus for lectures and salary support from the Bill & Melinda Gates Foundation and Gavi, the Vaccine Alliance, through Imperial College London for work outside this study. All other authors declare no competing interests.
