# Supplementary material for: Non-pharmaceutical interventions, vaccination, and the SARS-CoV-2 delta variant in England: a mathematical modelling study
Source: Lancet. 2021 Nov 13;398(10313):1825–35. doi: 10.1016/S0140-6736(21)02276-5 (PMC8550916; doi:10.1016/S0140-6736(21)02276-5)
Supplement: Supplementary appendix [file mmc1.pdf]

THE LANCET

Supplementary appendix

This appendix formed part of the original submission and has been peer reviewed. We post it as supplied by the authors.

Supplement to: Sonabend R, Whittles LK, Imai N, et al. Non-pharmaceutical interventions, vaccination, and the SARS-CoV-2 delta variant in England: a mathematical modelling study. *Lancet* 2021; published online Oct 27. http://dx.doi.org/10.1016/S0140-6736(21)02276-5.

Supplementary Material

CONTENTS

1 Model Description and Definitions 3
1.1 Overview of the model . . . 3
1.2 Reproduction number . . . 4
1.3 Fitting to data . . . 5

2 The Delta Variant 5
2.1 Transition from single to two-variant model . . . 5
2.2 Transmissibility compared to Alpha . . . 7
2.3 Vaccine effectiveness . . . 7
2.4 Increased severity . . . 7
2.5 Protection from previous infection . . . 7

3 Vaccination 7
3.1 Vaccine effectiveness . . . 9
3.2 Conditional dependencies of vaccine-immunity . . . 11
3.3 Vaccine-induced immunity . . . 11
3.4 Vaccine roll-out . . . 12
3.5 Waning of vaccine-induced immunity . . . 12

4 Model Fitting 13
4.1 Model compartments and parameters . . . 13
4.1.1 Parallel flows . . . 14
4.2 Equations . . . 16
4.2.1 Force of infection . . . 16
4.2.2 Pathway probabilities and rates . . . 19
4.2.3 Compartmental model equations . . . 21
4.3 Observation process . . . 31
4.3.1 Notation for distributions used in this section . . . 31
4.3.2 Hospital admissions and new diagnoses in hospital . . . 31
4.3.3 Hospital bed occupancy by confirmed COVID-19 cases . . . 32
4.3.4 Hospital, community and care homes COVID-19 deaths . . . 33
4.3.5 Serosurveys . . . 34
4.3.6 PCR testing . . . 34
4.3.7 Variant and Mutation data . . . 35
4.3.8 Full likelihood . . . 36
4.4 Reproduction number . . . 36

4.5 Fixed parameters . . . 38
4.6 Prior distributions . . . 40
4.7 Running the model . . . 42

5 Forward Projections 43
5.1 Transmissibility of SARS-CoV-2 . . . 43
5.1.1 NPI lifting: the new baseline . . . 43
5.1.2 School closures . . . 44
5.1.3 Seasonality . . . 44
5.2 Vaccine roll-out . . . 45
5.3 Counterfactual simulations . . . 45
5.4 Sensitivity analysis . . . 47

6 Software and Implementation 48

7 Supplementary Results 49
7.1 Model fit to data . . . 49
7.2 Simulation results . . . 59
7.3 Sensitivity analyses . . . 71
7.4 Waning vaccine effectiveness simulations . . . 76

Symbol Glossary 79

List of Figures 81

List of Tables 82

1 Model Description and Definitions

In this section we provide a brief description to our model along with key definitions. Full details about the fitting procedure, parameter assumptions, and model equations are provided in Section 4.

1.1 Overview of the model

We used a discrete-time stochastic compartmental model of SARS-CoV-2 transmission, illustrated in Figure S1, which has previously been described in detail in Knock et al. (2021) [1]. In short, the model is an extended SEIR-type model, stratified into 16 five-year age groups (0-4, 5-9, ..., 75-79), 80+, a group of care home residents (CHR) and a group of care home workers (CHW). Mixing between these groups is informed by survey data [2]. Upon infection with SARS-CoV-2, individuals enter an exposed compartment, before becoming infectious. A proportion of infectious individuals are assumed to develop symptoms, while the rest remain asymptomatic. All asymptomatic cases and a fraction of symptomatic cases recover naturally, while the rest of the symptomatic cases develop severe disease requiring hospitalisation. Of these, a proportion die at home, while the remainder are admitted to hospital. Hospital pathways are described in detail, with patients being either triaged before intensive care unit (ICU) admission, then admitted to ICU, before being transferred into general wards for stepdown recovery, or remaining in general beds throughout. Hospitalised cases are either confirmed as SARS-CoV-2 cases upon admission or may be tested and confirmed later during their stay.

Each compartment in the model is further stratified to account for vaccination status. We used four vaccination strata (Table S2 and Figure S1), which describe the recommended two-dose vaccination regimen (common to the three double-dose vaccines currently licensed and available for use in England: Oxford-AstraZeneca ChAdOx1 nCoV-19 (AZD1222) [3], Pfizer-BioNTech COVID-19 Vaccine BNT162b2 [4], and Moderna mRNA-1273 [5], henceforth referred to as AZ, PF, and Mod respectively), capturing a delay between receiving a dose and onset of dose-specific effectiveness.

As presented in Knock et al. (2021) [1], the model is utilised in two stages: an initial model fitting stage, to build posterior estimations of model parameters fitting to multiple epidemiological data streams, followed by a forecasting ('forward projection') stage, whereby the posterior estimates inform medium- and long-term projections for the pandemic trajectory as well as counterfactual "what if" scenarios. Polymerase chain reaction (PCR) tests and serology status are modelled with parallel flows.

The model has since been extended to include the spread of variants of concern (VOC). In the context of this paper, we consider Alpha (B.1.1.7) coexisting with the Delta variant (B.1.617.2). All references to 'Alpha' here refer to the Alpha variant and all other previously circulating variants. Before the emergence of Delta, we fit a one-variant model, and then switch to a two-variant model on 8 March 2021, where Delta is seeded at a region-specific date determined by the model fit. Additionally we added a second parallel serology flow, which allows us to fit to samples using two different serology assays, with different durations of seropositivity.

We now include waning of infection-induced immunity in the model. Individuals who have recovered from COVID-19 infection are protected against reinfection with the same variant of SARS-CoV-2 for an exponentially distributed duration with mean 6 years, after which they move back to the susceptible compartment. Other waning rates are considered within sensitivity analyses (Table S8). Further, we model asymmetrical cross-immunity between SARS-CoV-2 variants (Section 2.5). We will use the term 'susceptible' only to refer to individuals in compartment 'S', whereas 'uninfected' will refer to those in compartment 'S' or 'R' (recovered).

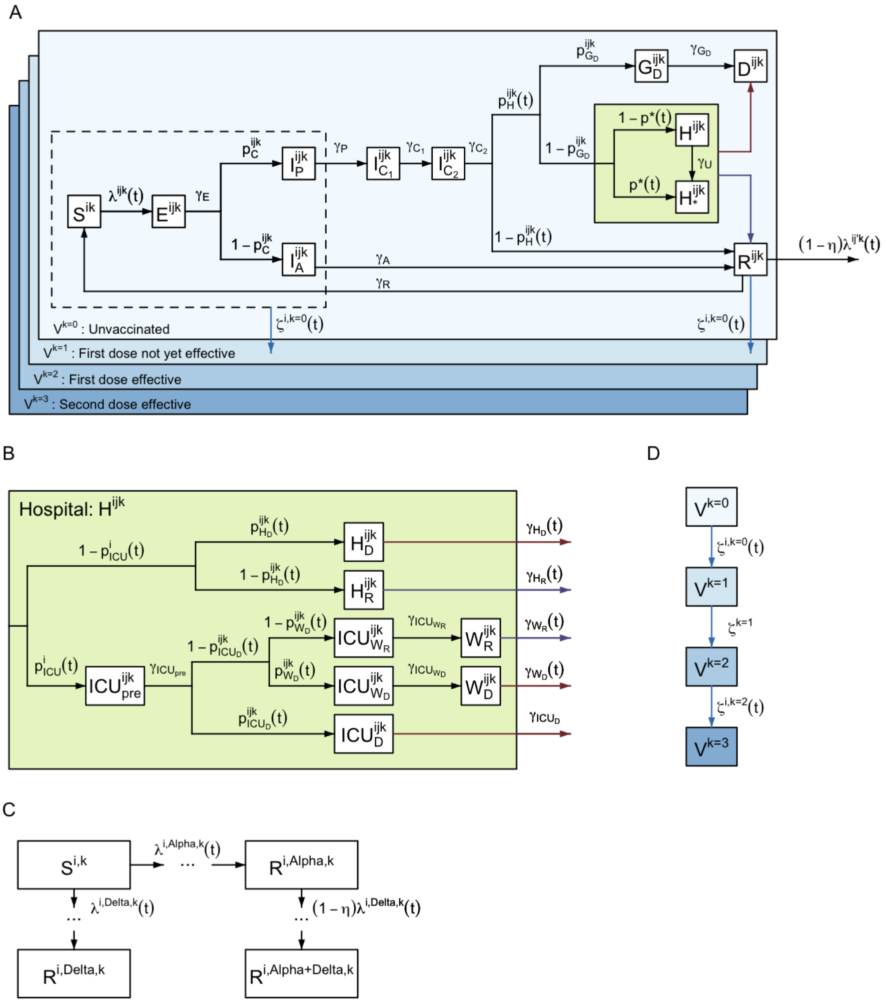

Figure S1: Model structure flow diagram with rates of transition between states. (A) Extended SEIR transmission model flow diagram overview. (B) Hospital flow diagram. (C) Vaccination flow diagram. (D) Multi-variant flow diagram showing possible infection with *Alpha*, *Delta*, or both in turn (*Alpha*➔*Delta*). All variables and parameters defined in tables S6, S7, and S8. Superscripts refer to the age or care home group ($i \in [0-4], [5-9], \ldots, [75-79], [80+], CHW, CHR$), variant ($j = Alpha, Delta$, or $Alpha$➔$Delta$), and vaccination stratum ($k = 0, \ldots, 3$).

1.2 Reproduction number

We use two definitions of the reproduction number throughout. We denote $R^j(t)$ as the reproduction number for variant j ($j = Alpha, Delta$) in the absence of immunity at time t. This is defined as the average number of secondary infections that an individual infected at time t with variant j would generate in an entirely susceptible and unvaccinated population. In contrast, the effective reproduction number, $R_e^j(t)$, for variant j

at time t is the number of secondary infections in the actual uninfected population, accounting for immunity (natural and vaccine-induced). Hence, by definition, $R_e^j(t) \leq R^j(t)$.

1.3 Fitting to data

The model is fitted to multiple data streams from each National Health Service (NHS) region in England, as described in Knock et al. (2021); this is summarised in Table S1.

2 The Delta Variant

The model switches to a two-variant set-up at 8 March 2021 to capture the emergence and spread of Delta, due to its proliferation to being the established dominant variant in all NHS regions of England. Key differences between the modelled variants are summarised in the next subsections, these are:

1. Date of introduction.
2. Transmissibility of Delta compared to Alpha.
3. Vaccine effectiveness against Delta.
4. Severity of Delta compared to Alpha.
5. Cross immunity conferred by prior infection with Alpha.

2.1 Transition from single to two-variant model

To account for the emergence of the Delta variant, while keeping the fitting process as efficient as possible, our dynamic transmission model is calibrated in two parts. We first run the inference using a single variant model until 8 March 2021. While the model only considers one variant in this part, some of the fitted epidemiological parameters (such as the transmission parameters or the probability of dying in hospitals) are time-varying. These time-varying parameters capture the trends observed in the data following changes in behaviour, in treatment practice, or the replacement by the Alpha variant of previously circulating variants. At 8 March 2021, we switch to a two-variant model and propagate the information from the first inference part in two ways. At the connection timepoint, models should be equivalent as there are no individuals infected with the Delta variant at this date. Infections with the Delta variant are then introduced on a region-specific date (t_{Delta}) determined by the model fits. 20 cases per million inhabitants are then seeded to the regions, distributed across the 7 days immediately following the initial seeding date t_{Delta}, and distributed evenly across the regional 5-year age groups as described by Office for National Statistics (ONS) mid-2020 population estimates [9].

We propagate the sample of filtered end model states on 8 March (obtained with our particle Markov chain Monte Carlo (MCMC) algorithm [1]) and use it as the initial distribution of the two-variant model. All compartments associated with being infected with the Delta variant are set to 0. Second, we propagate the information from the fitted posterior distributions of parameters common between parts 1 and 2. We fit a functional form (Table S9) to each of the parameters' marginal posterior distribution obtained in part 1 using maximum likelihood. These distributions are then used as priors for part 2.

The two-variant model was fitted to the same data streams as in Table S1, as well as the variant and mutation (VAM) dataset, which contains the daily number of variant tests by NHS region identified as Delta and as identified as Alpha (or other non-Delta circulating variants).

Data	Description	Source	Reference
Hospital deaths	Daily number of COVID-19 deaths reported by NHS England within 28 days of a positive result	Public Health England (PHE)	These data underlie the Gov.uk dashboard data [6]
Care home deaths	Daily number of deaths with COVID-19 mentioned as a cause on the death certificate and "care home", "hospice" or "other institution" as the place of death	ONS	These data underlie the Gov.uk dashboard data [6]
Community deaths	Daily number of deaths with COVID-19 mentioned as a cause on the death certificate and any place of death that is not one of "hospital", "care home", "hospice" or "other institution"	ONS	These data underlie the Gov.uk dashboard data [6]
ICU occupancy	Daily number of confirmed COVID-19 patients in ICU	Gov.uk Dashboard	[6]
General bed occupancy	Daily number of confirmed COVID-19 patients in non-ICU beds	Gov.uk Dashboard	[6]
Admissions	Daily number of confirmed COVID-19 patients admitted to hospital	Gov.uk Dashboard	[6]
Pillar 2 testing	Daily number of positive (cases) and negative PCR test results for individuals aged 25 or over	PHE	These data underlie the Gov.uk dashboard data [6]
REACT-1 testing	Real-time Assessment of Community Transmission (REACT) daily number of positive and negative PCR test results	REACT	[7]
Serology	Serology survey conducted on blood donors aged 15-65. Results using the EuroImmun and Roche N assays are used, fitting to each assay separately. EuroImmun results are only used up to (and including) 14th January 2020,	PHE	These data are collected as part of [8].
Vaccinations by age	Daily number of first and second-vaccine doses - reported in 5-year age groups	PHE	[6]

Table S1: Data sources and definitions.

2.2 Transmissibility compared to Alpha

We fit region-specific transmission advantages, σ, of the Delta variant compared to Alpha. These are initialised with uniform priors between 0 and 3 (Table S9), with an initial value of 1. Investigation into the generation interval for Delta compared to Alpha was unable to identify a significant difference between the two variants [10], hence we assume an identical generation interval for all variants within the model, with an increase in disease spread fully explained by differences in transmission.

2.3 Vaccine effectiveness

On top of the innate transmission advantage considered for Delta, transmission will also be boosted due to reduced vaccine effectiveness against Delta. Both the transmission advantage, σ, and variant-specific vaccine effectiveness are captured in the force of infection terms presented in Section 4.2. For full details on vaccine effectiveness against Delta, see Section 3 and in particular Table S3.

2.4 Increased severity

Following Sheikh et al. (2021) [11], we additionally multiply the relative probability of hospitalisation by 1.85 for those infected by Delta.

2.5 Protection from previous infection

The level of cross-protection from prior infection with non-Delta variants is difficult to quantify but in-vitro neutralisation studies found Delta was less susceptible to antibodies from previous infections [12]. We model asymmetric cross immunity between the two variants and assume that infection with Delta confers perfect immunity to infection with Alpha, whilst infection with Alpha is only partially protective against infection with Delta (see Table S8). In addition, for individuals infected by Delta following on from an infection with Alpha ($I_{C_2}^{i,Alpha \to Delta,k}$), we assume that, if the second infection is symptomatic, the probability of hospitalisation is reduced compared to individuals with no prior infection history. We consider this factor reduction equivalent to the conditional effectiveness against severe disease dependent on being symptomatic afforded by one dose of PF against Delta ($e_{SD|sympt}$ in Table S4 below). Probability of infection or hospitalisation by either variant 'resets' to base assumptions once an individual's acquired immunity wanes and they re-enter the susceptible compartment, $S^{i,k}$.

3 Vaccination

The Medicines and Healthcare Products Regulatory Agency issued temporary authorisation grants for both the Oxford-AstraZeneca (AZ) and the Pfizer-BioNTech (PF) vaccines in December 2020 [3, 4], and approved the Moderna (Mod) vaccine shortly after in January 2021 [5]. All three vaccines require two doses to be administered, with increasing levels of vaccine effectiveness seen after each dose. As such, our model considers four distinct vaccination strata (V_k, for $k \in \{0,1,2,3\}$) representing the four stages of vaccine effectiveness available, as detailed in Table S2 and illustrated in Figure S2. We model the United Kingdom (UK) vaccination strategy, i.e. to delay the second dose to, on average, 11 weeks after the first dose [13].

Individuals in our model move out of an unvaccinated (V_0) stratum at a rate determined by the age-specific vaccine roll-out and prioritisation strategy adopted by the UK government (Section 3.4). We only allow vaccination of individuals who are not symptomatic and not hospitalised, i.e. only individuals in the following

Vaccination stratum name	Number of doses	Vaccine effectiveness for that group	Mean duration	References
V_0	0	None	Determined by first dose vaccine roll-out	Section 3.4
V_1	1	None	3 weeks	[14, 15]
V_2	1	Full first dose effectiveness	In model-fit stage, determined by second dose vaccine roll-out; in simulation-stage, 8 weeks	[13, 14, 15]
V_3	2	Full second dose effectiveness	Infinite	Section 3.4

Table S2: Vaccination strata considered for individuals with the mean duration an individual spends in each strata and vaccine effectiveness at each stage.

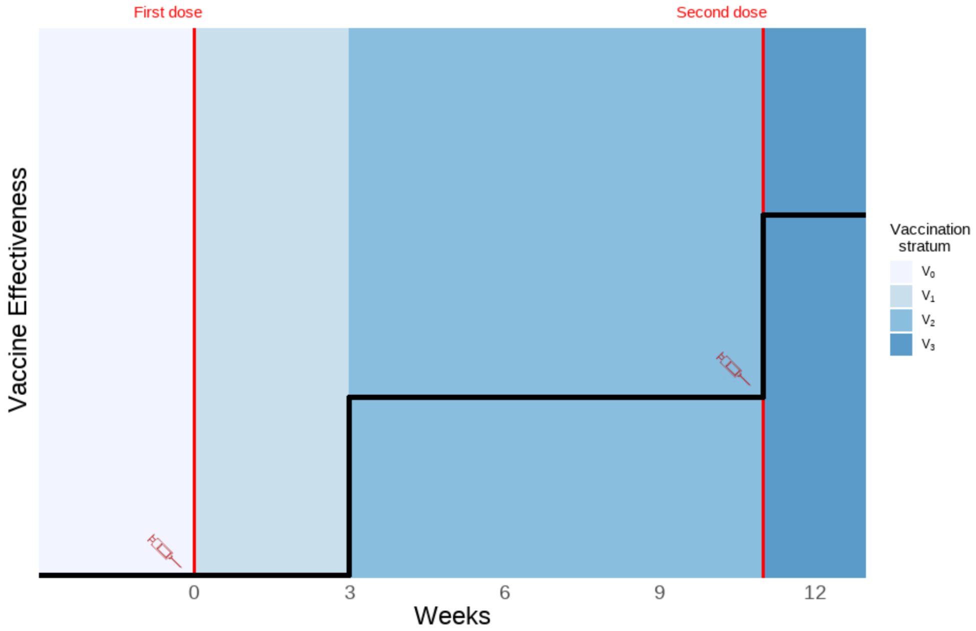

Figure S2: Vaccination strata duration and associated illustrative vaccine effectiveness. Red lines depict points at which a vaccine dose is administered. y-axis is an illustration of increasing vaccine effectiveness. Vaccination strata are defined in Table S2.

compartments can be vaccinated: susceptible (S), exposed (E), infected asymptomatic (I_A), infected pre-symptomatic (I_P) or recovered (R). Other compartments are also stratified by our four vaccination strata but for those there is no movement between vaccine strata (V_k).

Phase 2 trials for the AZ and PF vaccines suggested substantial increases in immunogenicity started approximately two to three weeks after receiving the first dose [14, 15]. We therefore assumed a 21-day delay between first dose injection and onset of dose-specific effectiveness. In our model, after receiving their first dose, individuals remain in V_1 for 21 days on average, during which the vaccine offers no protection. They then move on to the V_2 strata, where they stay for eight weeks on average and are protected with one dose. After this eight week period, individuals move on to the V_3 stratum where they are assumed to achieve maximal

protection offered by the two doses. This is illustrated in Figure S2. Note that whilst the data shows an average of 10 days between receiving the second dose and it taking full effect, the difference in our model reflects individuals receiving their second dose earlier than 11 weeks after the first. In our main analysis we assume no waning of vaccine-induced immunity, though briefly explore this as a sensitivity (Section 3.5).

3.1 Vaccine effectiveness

The assumed values for vaccine effectiveness (VE) are derived from both vaccine efficacy measured in clinical trials and vaccine effectiveness studies. Where possible, data from the UK have been used and represent effectiveness of dosing schedules with an 11 week gap between doses. We assumed that there are no significant differences in vaccine effectiveness by age, sex, or underlying health conditions [16, 17]. Table S3 summarises our vaccine effectiveness assumptions for the PF, AZ, and Mod vaccines. We assume that vaccine protection against symptomatic disease, as determined from the original trials and real-world data, also provides a similar level of protection against infection. We further assume that, in those individuals who do become infected after vaccination, onward transmission is also reduced [18]. Finally, we incorporate a higher overall level of vaccine effectiveness against hospitalisation and against death. Due to a high degree of uncertainty in the literature surrounding the effectiveness against the Delta variant, we adopted central, optimistic, and pessimistic values to be considered through sensitivity analysis. Note that our sensitivity analysis is concerned with Delta and therefore we vary VE against Delta only whilst always using central estimates for Alpha. We enforce that all Delta VE estimates are capped by the associated Alpha VE estimate. Figure S3 demonstrates our assumed values against the 95% credible intervals (CrI) of the associated literature, where UK-based studies are prioritised where available.

Vaccine effectiveness	Vaccine (dose)	Alpha	Delta (Central)	Delta (Pessimistic/Optimistic)	Informed by
Against death	AZ (1)	80%	80%	75%/80%	[19, 20]
	AZ (2)	95%	95%	95%/95%	[19, 20, 21]
	PF (1)	85%	85%	80%/85%	[19, 20]
	PF (2)	95%	95%	95%/95%	[19, 20]
	Mod	Assume same as PF for 1 and 2 doses			
Against severe disease	AZ (1)	80%	80%	75%/80%	[22, 23]
	AZ (2)	90%	90%	85%/90%	[23, 24, 25], Assumed greater than mild disease.
	PF (1)	85%	85%	80%/85%	[24]
	PF (2)	95%	95%	90%/95%	[25, 26], Assumed greater than mild disease.
	Mod	Assume same as PF for 1 and 2 doses			
Against mild disease	AZ (1)	50%	33%	20%/45%	[16, 25]
	AZ (2)	74%	58%	45%/70%	[16, 27, 28]
	PF (1)	50%	33%	20%/45%	[11, 23, 26, 28]
	PF (2)	93%	85%	78%/90%	[11, 26, 28, 29]
	Mod	Assume same as PF for 1 and 2 doses			
Against infection	AZ (1)	50%	33%	20%/45%	[11] Assumed same as effectiveness against disease.
	AZ (2)	74%	58%	45%/70%	[11]
	PF (1)	50%	33%	20%/45%	[11]
	PF (2)	93%	85%	78%/90%	[11]
	Mod	Assume same as PF for 1 and 2 doses			
Against infectiousness if infected	AZ (1)	45%	40%	35%/45%	[18] / Assumed
	AZ (2)	45%	40%	35%/45%	[18] / Assumed
	PF (1)	45%	40%	35%/45%	[18] / Assumed
	PF (2)	45%	40%	35%/45%	[18] / Assumed
	Mod	Assume same as PF for 1 and 2 doses			

Table S3: Vaccine effectiveness assumptions for AstraZeneca (AZ), Pfizer (PF), and Moderna (Mod).

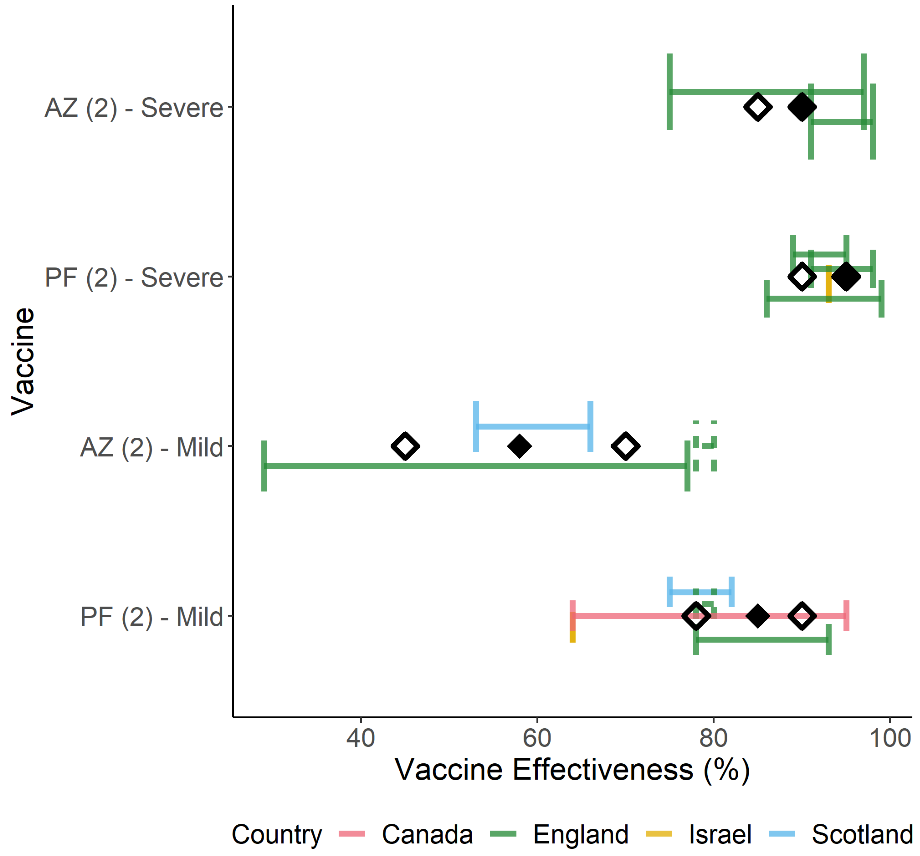

Figure S3: Comparison of our chosen Delta-specific VE values for two vaccine doses (Table S3) against associated 95% CrI values from the literature. Estimates taken from studies conducted in Canada [29], England [25, 23, 21, 28], Scotland [11], and Israel [30]. Black diamonds indicate our central assumptions, and hollow diamonds our pessimistic/optimistic assumptions. Dashed lines indicate a study that reported combined AZ/PF effect.

We model cases that require hospitalisation and are hospitalised, as well as cases that require hospitalisation but are not hospitalised; for this reason we refer to vaccine effectiveness against *severe disease* and not *hospitalisation*. Vaccine effectiveness against severe disease, conditional on symptoms, acts on transition to both this compartment of individuals and those admitted to hospital.

We do not model individual vaccines separately, instead vaccine compartments are type-agnostic, and for vaccine effectiveness we compute an age-dependent weighted mean of each vaccine's effectiveness (where weights for a given age group are the proportion of each vaccine type administered to that age group as of 19 July 2021). Whilst we assume vaccine effectiveness does not vary by age, the proportion of each vaccine (PF, AZ or Mod) administered to each age group varied substantially (Figure S4) and vaccine effectiveness varies between vaccines (Table S3), therefore our weighted vaccine-effectiveness varies by age.

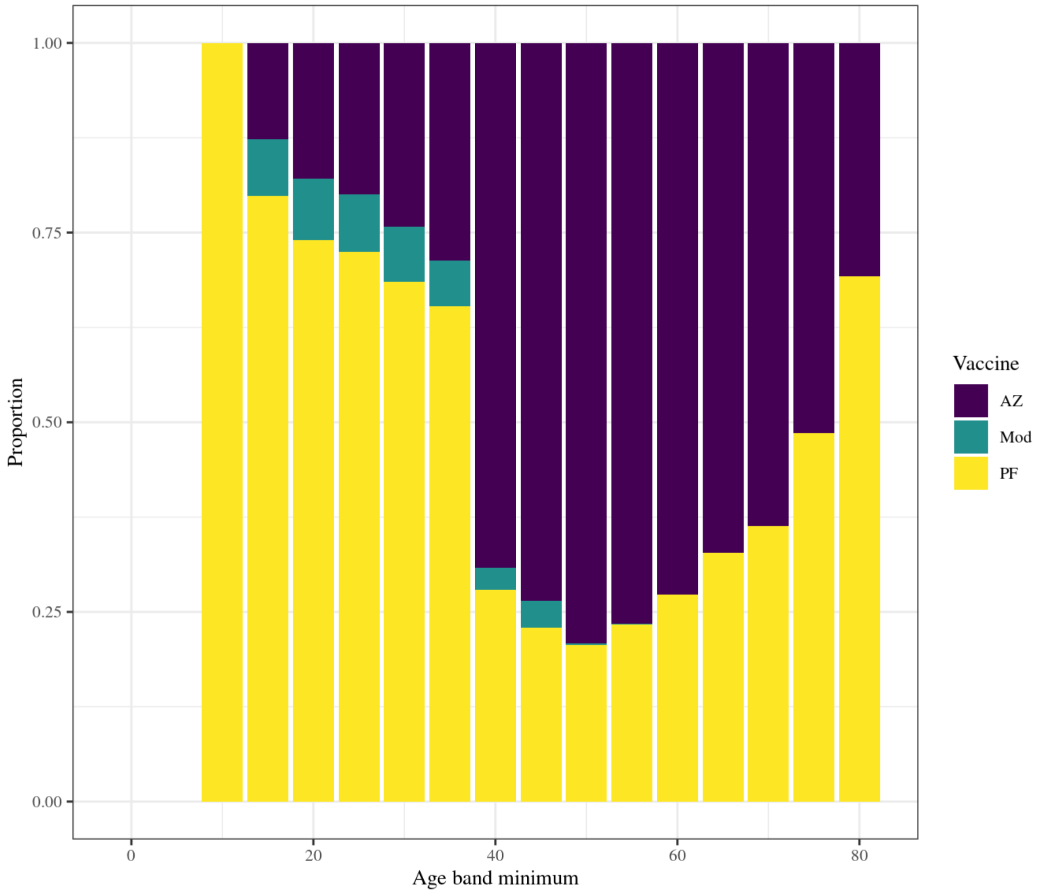

Figure S4: Proportion of each vaccine type: (Oxford-AstraZeneca (AZ), Pfizer-BioNTech (PF), Moderna (Mod)) dispensed to each five-year age band as of 31 July 2021. Data taken from PHE Immunisations database for vaccine delivery and ONS population estimates for each age group.

3.2 Conditional dependencies of vaccine-immunity

We present unconditional VE in Table S3 however our model is framed as a compartmental cascade of symptom severity, hence we convert unconditional effectiveness to conditional as detailed in Table S4.

3.3 Vaccine-induced immunity

In the absence of long-term follow-up studies on the duration of COVID-19 vaccine-induced immunity, we assume that IgG and T cell responses will be maintained for longer than the time horizon modelled. As such, we do not consider the waning of vaccine-protection in our main analyses [31], although we briefly explore this as a sensitivity (Section 3.5).

VE vs.	Symbol / Calculation
Infection	e_{inf}
Symptoms	e_{sympt}
Severe disease	e_{SD}
Death	e_{death}
Symptoms given infection	$e_{sympt\|inf} = \frac{e_{sympt} - e_{inf}}{1 - e_{inf}}$
Severe disease given symptoms	$e_{SD\|sympt} = \frac{e_{SD} - e_{sympt}}{(1 - e_{inf})(1 - e_{sympt\|inf})}$
Death given severe disease	$e_{death\|SD} = \frac{e_{death} - e_{SD}}{(1 - e_{inf})(1 - e_{sympt\|inf})(1 - e_{SD\|sympt})}$

Table S4: Conditional vaccine effectiveness values that we model.

3.4 Vaccine roll-out

The Joint Committee on Vaccination and Immunisation (JCVI) established an ordered list of individuals prioritised for vaccination in the UK, first prioritising care home residents and care home workers, and then other adults by decreasing age and clinical vulnerability [32, 33].

As our model is stratified using 5-year age classes, we model the vaccination of individuals aged 18-19 by assuming the uptake in the 15-19 age group is 2/5 of the uptake in the 20-24 year olds. Children under 18 years are not vaccinated in our model. We assume first and second doses were delivered in England between 8 December 2020 and 19 July 2021 as reported in age-stratified data received from PHE and the Department of Health and Social Care (DHSC) via the Scientific Pandemic Influenza Group on Modelling (SPI-M) (Figure S5) as reported on the COVID-19 dashboard [6]. It is not possible to identify individuals in our CHR and CHW groups from the data however as these are the first two priority groups we assume that all individuals vaccinated under 65 years were carehome workers and all individuals 65 years or over were carehome residents, up until the desired uptake in CHR and CHW is reached.

In forward projections we assume an average vaccine dose roll out of 1.9 million doses per week, based on past schedule, split between first and second doses to prioritise the latter, so that individuals having already received one dose receive their second dose approximately 11 weeks after their first. We assumed doses are split between NHS regions in proportion of their population size. Precise age-specific uptake assumptions are given in Table S13 and Section 5.2.

The UK government initially employed a 12-week prime-boost interval as supported by vaccine trials [27]. This was changed to a 8-week delay in mid-May 2021 to help combat the spread of Delta [34]. Initially altered only for those over the age of 50, this 8-week interval was expanded to the whole population in June 2021. By modelling an 11-week interval with immediate immunization after second dose, we create an approximate weighted average of timings between vaccine types and schedules.

3.5 Waning of vaccine-induced immunity

We briefly explored the impact of the waning of vaccine effectiveness over time and the results are presented in section 7.4. Since little quantitative evidence is currently available regarding the magnitude of waning VE,

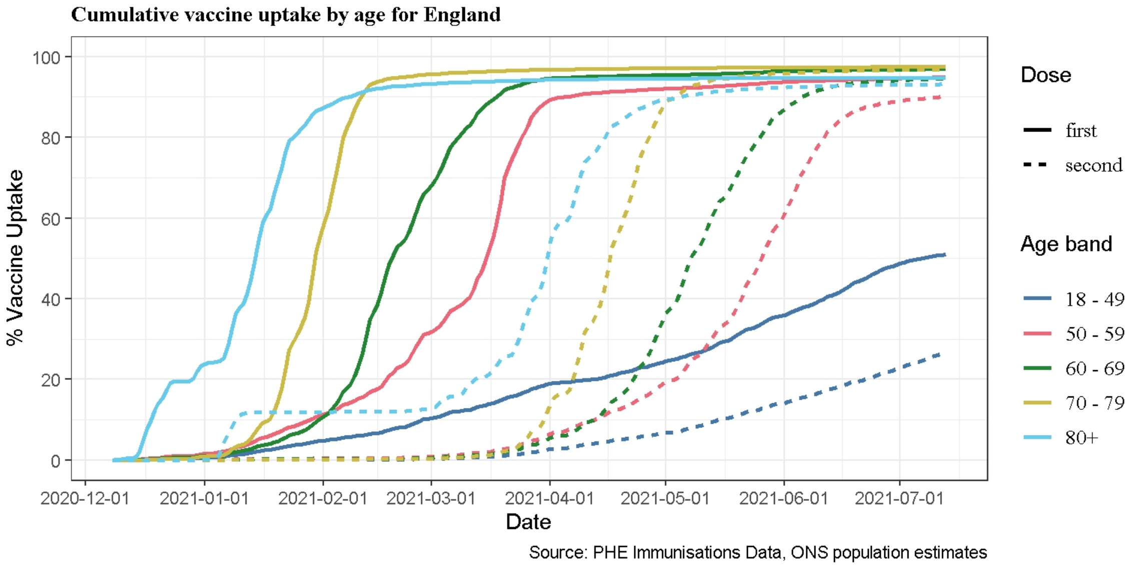

Figure S5: Cumulative vaccine uptake by age for England shown for first (solid lines) and second (dashed lines) doses, up to 19 July 2021. Shown as the proportion of the population age group of England (ONS).

we made the following simplifying assumptions:

1. The effect of waning VE is explored within our pessimistic model assumptions only (Tables S3, S10, S11).
2. Twenty weeks after receiving their second dose, all vaccinated individuals move to a new 'waned effectiveness' vaccine strata.
3. We assume that VE wanes against Delta in line with the latest PHE estimates [35]. As no estimates were available for the waned VE against infectiousness if infected, we assumed this was reduced by approximately 30% compared to the value before waning in our most pessimistic scenario (Table S5).
4. We assume the same ratio of VE decay for Alpha as for Delta (Table S5).

4 Model Fitting

4.1 Model compartments and parameters

In the following, i denotes the age or care home group of individuals ($i = [0,5), [5,10), \dots, [75-80), [80+)$, CHW, CHR), and j denotes their variant status ($j = Alpha$ for infection with Alpha, $Delta$ for infection with the Delta variant, or $Alpha \rightarrow Delta$ if an individual is infected with Delta immediately following infection from Alpha). Finally, k denotes the index of the vaccination stratum of individuals (with k corresponding to V_k as defined in Table S2).

$\zeta^{i,k}(t)$ is the rate of movement from vaccination stratum k to vaccination stratum $k+1$ at time t, for individuals in group i. This was calculated dynamically to match the number of daily doses aimed to be given to each group at time step t, given the number of daily doses available for distribution each day, the JCVI priority order, and the uptake in each group. Note that there is no movement out of vaccination stratum 3, so by

Vaccine effectiveness	Vaccine	Delta	Alpha
Against death	AZ	79%	79%
	PF/Mod	90%	90%
Against severe disease	AZ	77%	77%
	PF/Mod	90%	90%
Against mild disease	AZ	47%	67.2%
	PF/Mod	70%	86%
Against infection	AZ	47%	67.2%
	PF/Mod	70%	86%
Against infectiousness	AZ	25%	31.2%
	PF/Mod	25%	31.2%

Table S5: Waned vaccine effectiveness assumptions, starting 20 weeks after second dose, for AstraZeneca (AZ), Pfizer (PF), and Moderna (Mod) [35]. These values were used for sensitivity analysis only.

definition $\zeta^{i,3}(t) = 0$. Additionally we have no movement into vaccination stratum 0, so for ease of notation and equation simplicity we let $k = -1$ be a dummy vaccination strata with empty compartments and let $\zeta^{i,-1}(t) = 0$.

We define all model compartments and parameters in Table S6 below, and illustrate the model structure and flows between compartments in Figure S1 (this figure is copied again below in Figure S6 for easy reference). The model assumes discrete time and four time steps are taken per day.

4.1.1 Parallel flows

In addition to compartments involved in the transmission dynamics and clinical progression, there are three parallel flows which we use for fitting to testing data from surveys: (i) one for PCR testing and (ii) two for serology testing (Figure S7), with separate flows used for testing with the EuroImmun and Roche N assays.

The PCR flow is used for fitting to data from the REACT-1 study. Upon infection, an individual enters the PCR flow in a pre-positivity compartment ($T_{PCR_{pre}}$) before moving into the PCR positivity compartment ($T_{PCR_{pos}}$) and then ultimately into the PCR negativity compartment ($T_{PCR_{neg}}$).

We have two serology flows to allow us to assume different distributions for the time to seroreversion when fitting to samples tested with two different assays: EuroImmun and Roche N. EuroImmun was used for testing NHS Blood and Transplant (NHSBT) samples from the first wave onwards, while Roche N only started being used in November 2020. Roche N tests only for seropositivity resulting from infection, whereas EuroImmun does not distinguish between seropositivity resulting from infection or from vaccination. Since our serology flows only are designed to capture seroconversion resulting from infection, we do not fit to samples using the EuroImmun assay from 15th January 2021 onwards as we can expect the vaccination to impact beyond this. After a seroconversion period ($T_{sero^1_{pre}}$ for EuroImmun, $T_{sero^2_{pre}}$ for Roche N), individuals can seroconvert ($T_{sero^1_{pos}}$ for EuroImmun, $T_{sero^2_{pos}}$ for Roche N) or not ($T_{sero^1_{neg}}$ for EuroImmun, $T_{sero^2_{neg}}$ for Roche N) ; if they do seroconvert, they eventually serorevert to $T_{sero^1_{neg}}$ or $T_{sero^2_{neg}}$ accordingly.

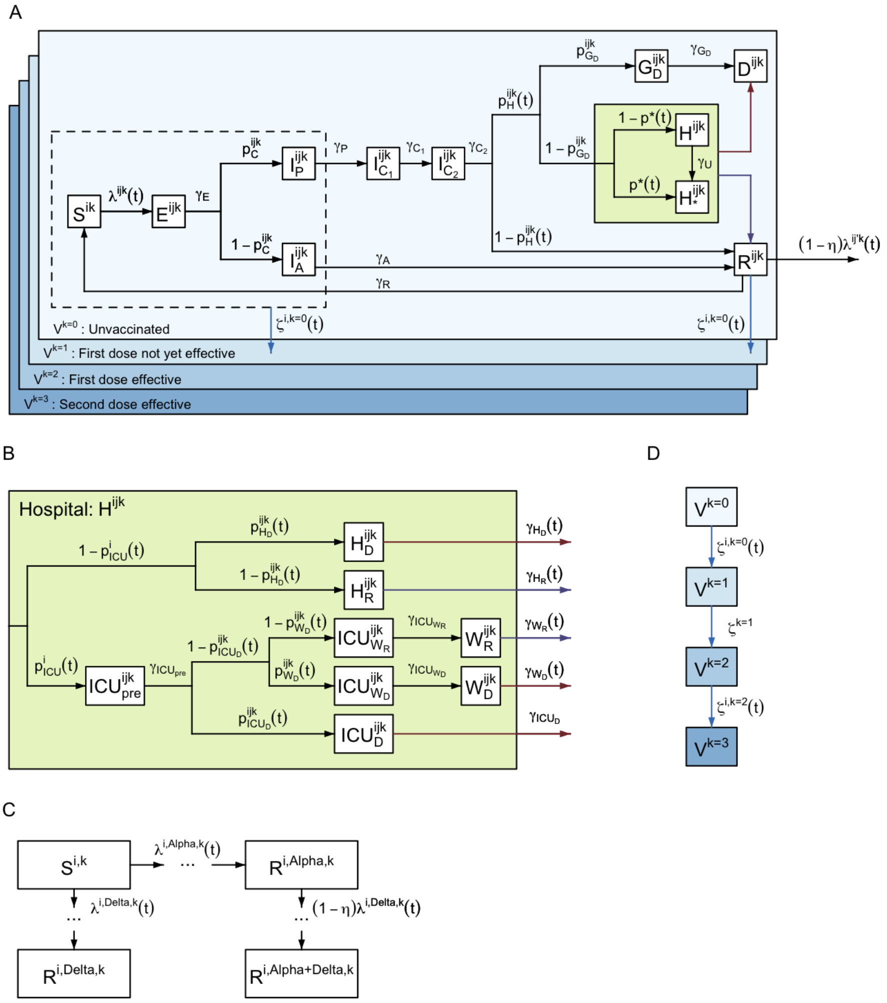

Figure S6: Model structure flow diagram with rates of transition between states. (A) Extended SEIR transmission model flow diagram overview. (B) Hospital flow diagram. (C) Vaccination flow diagram. (D) Multi-variant flow diagram showing possible infection with *Alpha*, *Delta*, or both in turn (*Alpha*➔*Delta*). All variables and parameters defined in tables S6, S7, and S8. Superscripts refer to the age or care home group ($i \in [0-4], [5-9], \ldots, [75-79], [80+], CHW, CHR$), variant ($j = Alpha, Delta$, or *Alpha*➔*Delta*), and vaccination stratum ($k = 0, \ldots, 3$). Identical to Figure S1, copied here for easy reference.

Compartment	Definition
$S^{i,k}(t)$	Susceptible
$E^{i,j,k}(t)$	Exposed (latent infection).
$I_A^{i,j,k}(t)$	Asymptomatic infected.
$I_P^{i,j,k}(t)$	Presymptomatic infected (infectious).
$I_{C_1}^{i,j,k}(t)$	Symptomatic infected (infectious).
$I_{C_2}^{i,j,k}(t)$	Symptomatic infected (not infectious).
$G_D^{i,j,k}(t)$	Severely diseased, leading to death (at home).
$D^{i,j,k}(t)$	Deceased (as a result of COVID-19).
$H_D^{i,j,k}(t)$	Hospitalised on general ward leading to death.
$H_R^{i,j,k}(t)$	Hospitalised on general ward leading to recovery.
$ICU_{pre}^{i,j,k}(t)$	Awaiting admission to ICU.
$ICU_D^{i,j,k}(t)$	Hospitalised in ICU, leading to death.
$ICU_{W_R}^{i,j,k}(t)$	Hospitalised in ICU, leading to recovery.
$ICU_{W_D}^{i,j,k}(t)$	Hospitalised in ICU, leading to death following step-down from ICU.
$W_R^{i,j,k}(t)$	Step-down recovery period.
$W_D^{i,j,k}(t)$	Step-down post-ICU period, leading to death.
$R^{i,j,k}(t)$	Recovered.

Table S6: Definitions of model compartments shown in Figure S1. i defines age group ($i \in \{[0,5),[5,10),\ldots,[75-80),[80+),CHW,CHR\}$), j denotes the variant ($j \in \{$ $Alpha$, $Delta$, $Alpha$➔$Delta$ $\}$ as defined in Section 4.1), k denotes vaccination strata ($k \in \{V_0,V_1,V_2,V_3\}$ as defined in Table S2). See Knock et al. (2021) [1] for further details.

4.2 Equations

4.2.1 Force of infection

We let $\chi^{i,j,k}$ be the susceptibility to variant j of a susceptible individual in group i and vaccine stratum k, relative to a non vaccinated individual (so that $\chi^{i,j,0} = 1$ for all i and j), given by

$$\chi^{i,j,k} = (1 - e_{inf}^{i,j,k}), \tag{1}$$

where $e_{inf}^{i,j,k}$ is the vaccine effectiveness against infection of variant j in vaccine strata k (Table S4), scaled across vaccine types according to the distribution presented in Figure S4.

We let $\xi^{i,j,k}$ be the infectivity of an individual in group i and vaccine stratum k infected with variant j relative to a non vaccinated individual infected with the Alpha variant (so that $\xi^{i,Alpha,0} = 1$). This infectivity captures both the vaccine effectiveness against infectiousness as presented in Table S3 and also the increased

Parameter	Definition
$\lambda^{i,j,k}(t)$	Force of infection.
γ_x	Rate of progression from compartment x.
γ_U	Rate at which unconfirmed hospital patients are confirmed as infected.
$p_C^{i,j,k}$	Probability of being symptomatic if infected.
$p_H^{i,j,k}$	Probability of admission to hospital, conditional on symptomatic infection.
$p_{G_D}^{i,j,k}$	Probability of death for severe symptomatic cases outside of hospital.
$p^*(t)$	Probability of COVID-19 diagnosis confirmed prior to admission to hospital.
$p_{ICU}^{i}(t)$	Probability of admission to ICU, conditional on hospitalisation.
$p_{H_D}^{i,j,k}(t)$	Probability of death for hospitalised cases not in ICU.
$p_{ICU_D}^{i,j,k}(t)$	Probability of death for cases in ICU.
$p_{W_D}^{i,j,k}(t)$	Probability of death for cases after discharge from ICU.
$\zeta^{i,k}(t)$	Rate of movement from vaccine strata k to $k+1$.
η	Protection from infection with *Delta* for those recovered from *Alpha* ($R^{i,Alpha,k}$), relative to those in the susceptible class ($S^{i,k}$).

Table S7: Definitions of model parameters shown in Figure S1. These parameters define the routes of transmission through model compartments defined in Table S6. i defines age group ($i \in \{[0,5),[5,10),\ldots,[75-80),[80+),CHW,CHR\}$), j denotes variant status j denotes the variant ($j \in \{$ *Alpha*, *Delta*, *Alpha*→*Delta* $\}$ as defined in Section 4.1), k denotes vaccination strata ($k \in \{V_0,V_1,V_2,V_3\}$ as defined in Table S2). Refer to Knock et al. (2021) [1] for further details of parameter fitting.

transmissibility of Delta compared to Alpha. As such $\xi^{i,j,k}$ is equal to

$$\xi^{i,j,k} = \begin{cases} (1-e_{ins}^{i,j,k}), & \text{if } j = Alpha, \\ \\ (1-e_{ins}^{i,j,k})\sigma, & \text{if } j \in \{Delta, Alpha{\to}Delta\}, \end{cases} \tag{2}$$

where $e_{ins}^{i,j,k}$ is the vaccine effectiveness against infectiousness of variant j in vaccine strata k as defined in Table S3, scaled across vaccine types according to the distribution presented in Figure S4, while σ is the region-specific transmission advantage of Delta as described in Section 2.2.

We let $\Theta_{i,j,k}(t)$ be the number of infectious individuals with variant j in group i and vaccination stratum k, weighted by infectivity, given by:

$$\Theta_{i,j,k}(t) = \xi^{i,j,k}\left(\theta_{I_A} I_A^{i,j,k}(t) + I_P^{i,j,k}(t) + I_{C_1}^{i,j,k}(t)\right). \tag{3}$$

where θ_{I_A} is the infectivity of an asymptomatic infected individual, relative to a symptomatic individual infected with the same variant, and in the same vaccination strata.

The force of infection, $\lambda^{i,j,k}(t)$, of variant $j \in \{Alpha, Delta\}$ on a susceptible individual in group $i \in \{[0,5), \ldots,[75,80),[80+), CHW, CHR\}$ and vaccination stratum $k = 0,1,\ldots,3$ is then given by

$$\lambda^{i,Alpha,k}(t) = \chi^{i,Alpha,k} \sum_{i'} m_{i,i'}(t) \sum_{k'} \Theta_{i',Alpha,k'}(t) \tag{4}$$

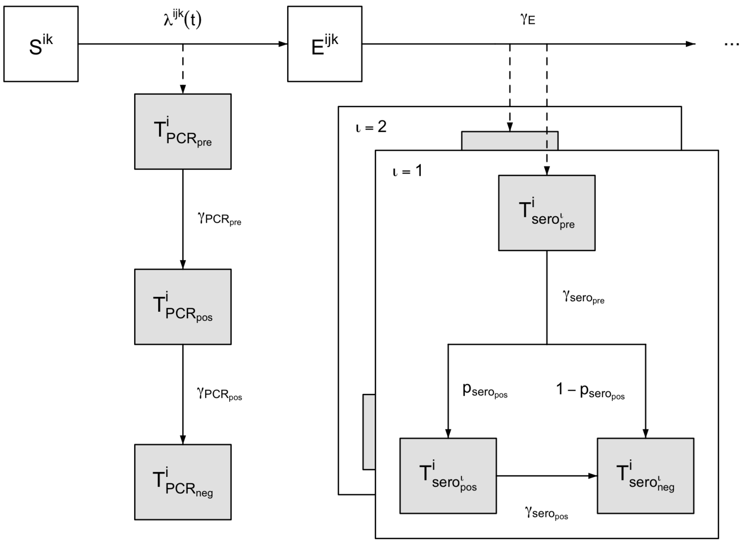

Figure S7: PCR positivity and seropositivity model structure flow diagram. Upon infection, an individual enters the pre-positive PCR compartment ($T_{PCR_{pre}}$) before moving into the PCR positivity compartment ($T_{PCR_{pos}}$) and then into the PCR negativity compartment ($T_{PCR_{neg}}$). After a seroreversion period ($T_{sero_{pre}}$), individuals can seroconvert ($T_{sero_{pos}}$) or not ($T_{sero_{neg}}$); if they do seroconvert, they eventually serorevert to $T_{sero_{pos}}$.

$$\lambda^{i,Delta,k}(t) = \chi^{i,Delta,k} \sum_{i'} m_{i,i'}(t) \sum_{k'} \left(\Theta_{i',Delta,k'}(t) + \Theta_{i',Alpha \to Delta,k'}(t)\right) \tag{5}$$

where $m_{i,i'}(t)$ is the (symmetric) time-varying person-to-person transmission rate from group i' to group i.

We let $\Lambda^{i,k}(t)$ be the total force of infection on a susceptible individual in group i and vaccination stratum k, i.e.

$$\Lambda^{i,k}(t) = \lambda^{i,Alpha,k}(t) + \lambda^{i,Delta,k}(t). \tag{6}$$

Note that there is zero force of infection of $Alpha$ on all recovered individuals. The force of infection of $Delta$ on an individual recovered from $Alpha$ in group i and vaccine stratum k is $(1-\eta)\lambda^{i,Delta,k}(t)$, where η is the cross immunity parameter Table S7. There is zero force of infection of $Delta$ on all individuals recovered from $Delta$ (or in the $Alpha \to Delta$ class).

Transmission between different age groups $(i,i') \in \{[0,5),\dots,[75,80),[80+)\}^2$ was parameterised as follows:

$$m_{i,i'}(t) = \beta(t)c_{i,i'}, \tag{7}$$

where $c_{i,i'}$ is the (symmetric) person-to-person contact rate between age group i and i', derived from pre-pandemic data from the POLYMOD survey [2] for the United Kingdom. For each region, the `socialmixr` package [36] was used to derive the contact matrix between different age groups $(i,i') \in \{[0,5),\dots,[75,80),[80+)\}^2$,

which was then scaled by the regional population demography to yield the required person-to-person daily contact rate matrix, $c_{i,i'}$.

$\beta(t)$ is the time-varying transmission rate which encompasses both changes over time in transmission efficiency (e.g. due to temperature) and temporal changes in the overall level of contacts in the population (due to changes in policy and behaviours).

We assumed $\beta(t)$ to be piecewise linear:

$$\beta(t) = \begin{cases} \beta_i, & \text{if } t \leq t_i,\ i = 1 \\ \frac{t_i - t}{t_i - t_{i-1}}\beta_{i-1} + \frac{t - t_{i-1}}{t_i - t_{i-1}}\beta_i, & \text{if } t_{i-1} < t \leq t_i,\ i \in \{2, \dots, 24\} \\ \beta_i & \text{if } t > t_i,\ i = 24 \end{cases} \tag{8}$$

with 24 change points t_i corresponding to major announcements and changes in COVID-19 related policy (Table S9).

We defined parameters representing transmission rates within care homes (between and among workers and residents), which were assumed to be constant over time. Parameter m_{CHW} represents the person-to-person transmission rate among care home workers and between care home workers and residents; m_{CHR} represents the person-to-person transmission rate among care home residents; these are defined as:

$$m_{CHW,CHW}(t) = m_{CHW,CHR}(t) = m_{CHW} \tag{9}$$

$$m_{CHR,CHR}(t) = m_{CHR} \tag{10}$$

Transmission between the general population and care home workers was assumed to be similar to that within the general population, accounting for the average age of care home workers, with, for $i \in \{[0,5), \dots, [75,80), [80+)\}$,

$$m_{i,CHW}(t) = \beta(t)c_{i,CHW}, \tag{11}$$

where $c_{i,CHW}$ is the mean of $c_{i,[25,30)}, c_{i,[30,35)}, \dots, c_{i,[60,65)}$ (i.e. of the age groups that the care home workers are drawn from).

Transmission between the general population and care home residents was assumed to be similar to that between the general population and the 80+ age group, adjusted by a reduction factor ε (which was inferred, see Table S9), such that, for $i \in \{[0,5), \dots, [75,80), [80+)\}$,

$$m_{i,CHR} = \varepsilon\beta(t)c_{i,80+}. \tag{12}$$

These represent contact between visitors from the general community and care home residents. This might involve a slightly different age profile than the age profile of the contact made by people in the 80+ age group.

4.2.2 Pathway probabilities and rates

The movement between model compartments is primarily dictated by the parameters $p_x^{i,j,k}$, defining the probability of progressing to compartment x (Table S7), as well as rate parameters γ. These parameters vary between age groups (i), variant of infection (j), and vaccine strata (k). This section outlines how these differences are formally defined and calculated.

The probability that an individual will have a symptomatic infection given that they have been infected is given by

$$p_C^{i,j,k} = p_C^i \left(1 - e^{i,j,k}_{sympt|inf}\right), \tag{13}$$

where p_C^i is given in [1].

The probability that an individual has severe disease requiring hospitalisation given that they have symptoms is given by

$$p_H^{i,j,k}(t) = h_H(t)\psi_H^i \left(1 - e^{i,j,k}_{SD|sympt}\right), \tag{14}$$

where ψ_H^i is given in [1] (with a slight amendment that now $\psi_H^{CHR} = 1$) and $h_H(t)$ has a piecewise linear form with changepoints defined as follows:

$$h_H = (t)\begin{cases} p_{H,1}^{max} & \text{on (and before) 2020-10-01,} \\ p_{H,2}^{max} & \text{on 2020-12-15,} \\ p_{H,3}^{max} & \text{on (and after) 2021-02-02.} \end{cases} \tag{15}$$

The probability that an individual dies in the community (or a care home if $i = CHR$) given they have severe disease is

$$p_{G_D}^i(t) = \begin{cases} p_{G_D} & \text{if } i \neq CHR, \\ p_{G_D}^{CHR} & \text{if } i = CHR. \end{cases} \tag{16}$$

The probability that an individual will be admitted to ICU given that they have been hospitalised is

$$p_{ICU}^i(t) = h_{ICU}(t)\psi_{ICU}^i, \tag{17}$$

where ψ_{ICU}^i is given in [1] and $h_{ICU}(t)$ has a piecewise linear form with changepoints defined as follows:

$$h_{ICU} = (t)\begin{cases} p_{ICU,1}^{max} & \text{on (and before) 2020-04-01,} \\ p_{ICU,2}^{max} & \text{on 2020-06-01.} \end{cases} \tag{18}$$

The probability that an individual will die in general beds given that they are not admitted to ICU is

$$p_{H_D}^{i,j,k}(t) = \min\left\{p_{H_D}^{max} h_D(t)\psi_{H_D}^i e^{i,j,k}_{death|SD}\omega_j, 1\right\}, \tag{19}$$

where $\psi_{H_D}^i$ is given in [1], $h_D(t)$ has a piecewise linear form with changepoints defined as follows:

$$h_D = (t)\begin{cases} 1 & \text{on (and before) 2020-04-01,} \\ \mu_{D,1}^{max} & \text{on 2020-06-01,} \\ \mu_{D,1}^{max} & \text{on 2020-10-01,} \\ \mu_{D,2}^{max} & \text{on 2020-12-15,} \\ \mu_{D,2}^{max} & \text{on 2021-01-15,} \\ \mu_{D,1}^{max} & \text{on (and after) 2021-02-01,} \end{cases} \tag{20}$$

and ω_j is a multiplier accounting for the increased severity of *Delta* (see Section 2.4):

$$\omega_j = \begin{cases} 1 & \text{if } j = Alpha, \\ 1.85 & \text{if } j = Delta. \end{cases} \tag{21}$$

The probability that an individual dies in ICU given that they are not admitted to ICU is

$$p^{i,j,k}_{ICU_D}(t) = p^{max}_{ICU_D} h_D(t) \psi^i_{ICU_D} e^{i,j,k}_{death|SD} \tag{22}$$

where $\psi^i_{ICU_D}$ is given in [1].

The probability that an individual who has been in ICU dies in stepdown beds given that they have not died in ICU is

$$p^{i,j,k}_{W_D}(t) = p^{max}_{W_D} h_D(t) \psi^i_{W_D} e^{i,j,k}_{death|SD}, \tag{23}$$

where $\psi^i_{W_D}$ is given in [1].

Finally, the probability of individuals having had a COVID-19 diagnosis confirmed prior to admission to hospital, $p^*(t)$ has a piecewise linear form with changepoints defined as follows:

$$p^*(t) = \begin{cases} 0.1 & \text{on (and before) 2020-03-15,} \\ 0.42 & \text{on 2020-07-01,} \\ 0.2 & \text{on 2020-09-09,} \\ 0.45 & \text{on (and after) 2021-06-27.} \end{cases} \tag{24}$$

These were informed by data on COVID-19 admissions and inpatient diagnoses for England from NHS England [37].

In addition, the duration rates for some hospital compartments are time-varying to account for changes in length of stay over time. We let

$$\gamma_{H_R}(t) = h_\gamma(t)\gamma_{H_R} \tag{25}$$
$$\gamma_{H_D}(t) = h_\gamma(t)\gamma_{H_D} \tag{26}$$
$$\gamma_{W_R}(t) = h_\gamma(t)\gamma_{W_R} \tag{27}$$
$$\gamma_{W_D}(t) = h_\gamma(t)\gamma_{W_D} \tag{28}$$
$$\tag{29}$$

where $h_\gamma(t)$ has a piecewise linear form with changepoints given by

$$h_\gamma(t) = \begin{cases} 1 & \text{on (and before) 2020-12-01,} \\ 1/\mu_{\gamma_H,1} & \text{on 2021-01-01,} \\ 1/\mu_{\gamma_H,2} & \text{on 2021-03-01,} \\ 1/\mu_{\gamma_H,3} & \text{on (and after) 2021-06-15.} \end{cases} \tag{30}$$

4.2.3 Compartmental model equations

To clearly illustrate the model dynamics, we describe a deterministic version of the model in differential equations (32)-(72), followed by the stochastic implementation used in the analysis. Full definitions of compartments and model parameters are set out in Tables S6 and S7. Unless otherwise specified, $\sum_j$ refers to

the sum across all combinations of variants (i.e. $j \in \{Alpha, Delta, Alpha{\to}Delta\}$), and $\sum_k$ refers to the sum across all vaccination strata ($k \in \{0,1,2,3\}$).

In the following model equations we use $\mathbb{1}_A$ as an indicator function, such that

$$\mathbb{1}_A(j) := \begin{cases} 1 & \text{if } j \in A, \\ 0 & \text{if } j \notin A. \end{cases} \tag{31}$$

Further note that we split some compartments in two distinct compartments. For example, the exposed class, $E^{i,j,k}$, is modelled via two separate compartments, $E^{i,j,k,1}$ and $E^{i,j,k,2}$ (equations (33) and (34)). This is to be able to capture a non exponentially distributed duration of stay in certain compartments; the split allows to model the duration of stay as an Erlang distribution instead (sum of independent exponential distributions) [38].

$$\frac{dS^{i,k}(t)}{dt} = \zeta^{i,k-1}(t)S^{i,k-1}(t) - \left(\zeta^{i,k}(t) + \Lambda^{i,k}(t)\right)S^{i,k}(t) + \gamma_R \sum_j R^{i,j,k}(t) \tag{32}$$

$$\begin{aligned}\frac{dE^{i,j,k,1}(t)}{dt} &= \mathbb{1}_{\{Alpha,Delta\}}(j)\lambda^{i,j,k}(t)S^{i,k}(t) + \mathbb{1}_{\{Alpha\to Delta\}}(j)\,(1-\eta)\,\lambda^{i,Delta,k}(t)R^{i,Alpha,k}(t) \\ &\quad + \zeta^{i,k-1}(t)E^{i,j,k-1,1}(t) - \left(\gamma_E + \zeta^{i,k}(t)\right)E^{i,j,k,1}(t)\end{aligned} \tag{33}$$

$$\frac{dE^{i,j,k,2}(t)}{dt} = \gamma_E E^{i,j,k,1}(t) + \zeta^{i,k-1}(t)E^{i,j,k-1,2}(t) - \left(\gamma_E + \zeta^{i,k}(t)\right)E^{i,j,k,2}(t) \tag{34}$$

$$\frac{dI_A^{i,j,k}(t)}{dt} = \left(1 - p_C^{i,j,k}\right)\gamma_E E^{i,j,k,2}(t) + \zeta^{i,k-1}(t)I_A^{i,j,k-1}(t) - \left(\gamma_A + \zeta^{i,k}(t)\right)I_A^{i,j,k}(t) \tag{35}$$

$$\frac{dI_P^{i,j,k}(t)}{dt} = p_C^{i,j,k}\gamma_E E^{i,j,k,2}(t) + \zeta^{i,k-1}(t)I_P^{i,j,k-1}(t) - \left(\gamma_P + \zeta^{i,k}(t)\right)I_P^{i,j,k}(t) \tag{36}$$

$$\frac{dI_{C_1}^{i,j,k}(t)}{dt} = \gamma_P I_P^{i,j,k}(t) - \gamma_{C_1} I_{C_1}^{i,j,k}(t) \tag{37}$$

$$\frac{dI_{C_2}^{i,j,k}(t)}{dt} = \gamma_{C_1} I_{C_1}^{i,j,k}(t) - \gamma_{C_2} I_{C_2}^{i,j,k}(t) \tag{38}$$

$$\frac{dG_D^{i,j,k,1}(t)}{dt} = p_H^{i,j,k}(t)p_{G_D}^{i,j,k}\gamma_{C_2} I_{C_2}^{i,j,k}(t) - \gamma_{G_D} G_D^{i,j,k,1}(t) \tag{39}$$

$$\frac{dG_D^{i,j,k,2}(t)}{dt} = \gamma_{G_D} G_D^{i,j,k,1}(t) - \gamma_{G_D} G_D^{i,j,k,2}(t) \tag{40}$$

$$\begin{aligned}\frac{dICU_{pre}^{i,j,k}(t)}{dt} &= p_H^{i,j,k}(t)\left(1 - p_{G_D}^{i,j,k}\right)(1 - p^*(t))\,p_{ICU}^{i}(t)\gamma_{C_2} I_{C_2}^{i,j,k}(t) \\ &\quad - \left(\gamma_{ICU_{pre}} + \gamma_U\right)ICU_{pre}^{i,j,k}(t)\end{aligned} \tag{41}$$

$$\begin{aligned}\frac{dICU_{pre^*}^{i,j,k}(t)}{dt} &= p_H^{i,j,k}(t)\left(1 - p_{G_D}^{i,j,k}\right)p^*(t)p_{ICU}^{i}(t)\gamma_{C_2} I_{C_2}^{i,j,k}(t) - \gamma_{ICU_{pre}} ICU_{pre^*}^{i,j,k}(t) \\ &\quad + \gamma_U ICU_{pre}^{i,j,k}(t)\end{aligned} \tag{42}$$

$$\begin{aligned}\frac{dICU_{W_R}^{i,j,k}(t)}{dt} &= \left(1 - p_{ICU_D}^{i,j,k}(t)\right)\left(1 - p_{W_D}^{i,j,k}(t)\right)\gamma_{ICU_{pre}} ICU_{pre}^{i,j,k}(t) \\ &\quad - \left(\gamma_{ICU_{W_R}} + \gamma_U\right)ICU_{W_R}^{i,j,k}(t)\end{aligned} \tag{43}$$

$$\frac{dICU_{W_{R^*}}^{i,j,k}(t)}{dt} = \left(1-p_{ICU_D}^{i,j,k}(t)\right)\left(1-p_{W_D}^{i,j,k}(t)\right)\gamma_{ICU_{pre}}ICU_{pre^*}^{i,j,k}(t) - \gamma_{ICU_{W_R}}ICU_{W_{R^*}}^{i,j,k}(t) + \gamma_U ICU_{W_R}^{i,j,k}(t) \tag{44}$$

$$\frac{dICU_{W_D}^{i,j,k}(t)}{dt} = \left(1-p_{ICU_D}^{i,j,k}(t)\right)p_{W_D}^{i,j,k}(t)\gamma_{ICU_{pre}}ICU_{pre}^{i,j,k}(t) - \left(\gamma_{ICU_{W_D}}+\gamma_U\right)ICU_{W_D}^{i,j,k}(t) \tag{45}$$

$$\frac{dICU_{W_{D^*}}^{i,j,k}(t)}{dt} = \left(1-p_{ICU_D}^{i,j,k}(t)\right)p_{W_D}^{i,j,k}(t)\gamma_{ICU_{pre}}ICU_{pre^*}^{i,j,k}(t) - \gamma_{ICU_{W_D}}ICU_{W_{D^*}}^{i,j,k}(t) + \gamma_U ICU_{W_D}^{i,j,k}(t) \tag{46}$$

$$\frac{dICU_D^{i,j,k,1}(t)}{dt} = p_{ICU_D}^{i,j,k}(t)\gamma_{ICU_{pre}}ICU_{pre}^{i,j,k}(t) - \left(\gamma_{ICU_D}+\gamma_U\right)ICU_D^{i,j,k,1}(t) \tag{47}$$

$$\frac{dICU_D^{i,j,k,2}(t)}{dt} = \gamma_{ICU_D}ICU_D^{i,j,k,1}(t) - \left(\gamma_{ICU_D}+\gamma_U\right)ICU_D^{i,j,k,2}(t) \tag{48}$$

$$\frac{dICU_{D^*}^{i,j,k,1}(t)}{dt} = p_{ICU_D}^{i,j,k}(t)\gamma_{ICU_{pre}}ICU_{pre^*}^{i,j,k}(t) - \gamma_{ICU_D}ICU_{D^*}^{i,j,k,1}(t) + \gamma_U ICU_D^{i,j,k,1}(t) \tag{49}$$

$$\frac{dICU_{D^*}^{i,j,k,2}(t)}{dt} = \gamma_{ICU_D}ICU_{D^*}^{i,j,k,1}(t) - \gamma_{ICU_D}ICU_{D^*}^{i,j,k,2}(t) + \gamma_U ICU_D^{i,j,k,2}(t) \tag{50}$$

$$\frac{dW_R^{i,j,k,1}(t)}{dt} = \gamma_{ICU_{W_R}}ICU_{W_R}^{i,j,k}(t) - \left(\gamma_{W_R}(t)+\gamma_U\right)W_R^{i,j,k,1}(t) \tag{51}$$

$$\frac{dW_R^{i,j,k,2}(t)}{dt} = \gamma_{W_R}(t)W_R^{i,j,k,1}(t) - \left(\gamma_{W_R}(t)+\gamma_U\right)W_R^{i,j,k,2}(t) \tag{52}$$

$$\frac{dW_{R^*}^{i,j,k,1}(t)}{dt} = \gamma_{ICU_{W_R}}ICU_{W_{R^*}}^{i,j,k}(t) - \gamma_{W_R}(t)W_{R^*}^{i,j,k,1}(t) + \gamma_U W_R^{i,j,k,1}(t) \tag{53}$$

$$\frac{dW_{R^*}^{i,j,k,2}(t)}{dt} = \gamma_{W_R}(t)W_{R^*}^{i,j,k,1}(t) - \gamma_{W_R}(t)W_{R^*}^{i,j,k,2}(t) + \gamma_U W_R^{i,j,k,2}(t) \tag{54}$$

$$\frac{dW_D^{i,j,k}(t)}{dt} = \gamma_{ICU_{W_D}}ICU_{W_D}^{i,j,k}(t) - \left(\gamma_{W_D}(t)+\gamma_U\right)W_D^{i,j,k}(t) \tag{55}$$

$$\frac{dW_{D^*}^{i,j,k}(t)}{dt} = \gamma_{ICU_{W_D}}ICU_{W_{D^*}}^{i,j,k}(t) - \gamma_{W_D}(t)W_{D^*}^{i,j,k}(t) + \gamma_U W_D^{i,j,k}(t) \tag{56}$$

$$\frac{dH_R^{i,j,k}(t)}{dt} = p_H^{i,j,k}(t)\left(1-p_{G_D}^{i,j,k}\right)\left(1-p^*(t)\right)\left(1-p_{ICU}^i(t)\right)\left(1-p_{H_D}^{i,j,k}(t)\right)\gamma_{C_2}I_{C_2}^{i,j,k}(t) - \left(\gamma_{H_R}(t)+\gamma_U\right)H_R^{i,j,k}(t) \tag{57}$$

$$\frac{dH_{R^*}^{i,j,k}(t)}{dt} = p_H^{i,j,k}(t)\left(1-p_{G_D}^{i,j,k}\right)p^*(t)\left(1-p_{ICU}^i(t)\right)\left(1-p_{H_D}^{i,j,k}(t)\right)\gamma_{C_2}I_{C_2}^{i,j,k}(t) + \gamma_U H_R^{i,j,k}(t) - \gamma_{H_R}(t)H_{R^*}^{i,j,k}(t) \tag{58}$$

$$\frac{dH_D^{i,j,k,1}(t)}{dt} = p_H^{i,j,k}(t)\left(1-p_{G_D}^{i,j,k}\right)\left(1-p^*(t)\right)\left(1-p_{ICU}^i(t)\right)p_{H_D}^{i,j,k}(t)\gamma_{C_2}I_{C_2}^{i,j,k}(t) - \left(\gamma_{H_D}(t)+\gamma_U\right)H_D^{i,j,k,1}(t) \tag{59}$$

$$\frac{dH_D^{i,j,k,2}(t)}{dt} = \gamma_{H_D}(t)H_D^{i,j,k,1}(t) - \left(\gamma_{H_D}(t)+\gamma_U\right)H_D^{i,j,k,2}(t) \tag{60}$$

$$\frac{dH_{D^*}^{i,j,k,1}(t)}{dt} = p_H^{i,j,k}(t)\left(1-p_{G_D}^{i,j,k}\right)p^*(t)\left(1-p_{ICU}^i(t)\right)\,p_{H_D}^{i,j,k}(t)\gamma_{C_2}I_{C_2}^{i,j,k}(t)+\gamma_U H_D^{i,j,k,1}(t) - \gamma_{H_D}(t)H_{D^*}^{i,j,k,1}(t) \tag{61}$$

$$\frac{dH_{D^*}^{i,j,k,2}(t)}{dt} = \gamma_{H_D}(t)H_{D^*}^{i,j,k,1}(t)-\gamma_{H_D}(t)H_{D^*}^{i,j,k,2}(t)+\gamma_U H_D^{i,j,k,2}(t) \tag{62}$$

$$\begin{aligned}\frac{dR^{i,j,k}(t)}{dt} &= \gamma_A I_A^{i,j,k}(t)+\left(1-p_H^{i,j,k}(t)\right)\gamma_{C_2}I_{C_2}^{i,j,k}(t)+\gamma_{H_R}(t)\left(H_R^{i,j,k}(t)+H_{R^*}^{i,j,k}(t)\right)\\ &+\gamma_{W_R}(t)\left(W_R^{i,j,k,2}(t)+W_{R^*}^{i,j,k,2}(t)\right)+\zeta^{i,k-1}(t)R^{i,j,k-1}(t)-\left(\gamma_R+\zeta^{i,k}(t)\right)R^{i,j,k}(t)\\ &-\mathbb{1}_{\{Alpha\}}(j)\,(1-\eta)\,\lambda^{i,Delta,k}(t)R^{i,j,k}(t)\end{aligned} \tag{63}$$

$$\frac{dT^i_{sero^1_{pre}}(t)}{dt} = -\gamma_{sero_{pre}}T^i_{sero^1_{pre}}(t)+\sum_j\sum_k\gamma_E E^{i,j,k,2}(t) \tag{64}$$

$$\frac{dT^i_{sero^1_{pos}}(t)}{dt} = p_{sero_{pos}}\gamma_{sero_{pre}}T^i_{sero^1_{pre}}(t)-\gamma_{sero^1_{pos}}T^i_{sero^1_{pos}}(t) \tag{65}$$

$$\frac{dT^i_{sero^1_{neg}}(t)}{dt} = \left(1-p_{sero_{pos}}\right)\gamma_{sero_{pre}}T^i_{sero^1_{pre}}(t)+\gamma_{sero^1_{pos}}T^i_{sero^1_{pos}}(t) \tag{66}$$

$$\frac{dT^i_{sero^2_{pre}}(t)}{dt} = -\gamma_{sero_{pre}}T^i_{sero^2_{pre}}(t)+\sum_j\sum_k\gamma_E E^{i,j,k,2}(t) \tag{67}$$

$$\frac{dT^i_{sero^2_{pos}}(t)}{dt} = p_{sero_{pos}}\gamma_{sero_{pre}}T^i_{sero^2_{pre}}(t)-\gamma_{sero^2_{pos}}T^i_{sero^2_{pos}}(t) \tag{68}$$

$$\frac{dT^i_{sero^2_{neg}}(t)}{dt} = \left(1-p_{sero_{pos}}\right)\gamma_{sero_{pre}}T^i_{sero^2_{pre}}(t)+\gamma_{sero^2_{pos}}T^i_{sero^2_{pos}}(t) \tag{69}$$

$$\frac{dT^i_{PCR_{pre}}(t)}{dt} = -\gamma_{PCR_{pre}}T^i_{PCR_{pre}}(t)+\sum_k\left(\lambda^{i,Alpha,k}(t)+\lambda^{i,Delta,k}(t)\right)S^{i,k}(t) \tag{70}$$

$$\frac{dT^i_{PCR_{pos}}(t)}{dt} = \gamma_{PCR_{pre}}T^i_{PCR_{pre}}(t)-\gamma_{PCR_{pos}}T^i_{PCR_{pos}}(t) \tag{71}$$

$$\frac{dT^i_{PCR_{neg}}(t)}{dt} = \gamma_{PCR_{pos}}T^i_{PCR_{pos}}(t). \tag{72}$$

We used the tau-leap method [39] to create a stochastic, time-discretised version of the model described in equations (75) - (206), taking four update steps per day ($dt = 0.25$ days).

For each time step, the model iterated through the procedure described below. In the following, we introduce a small abuse of notation: for transitions involving multiple onward compartments (e.g. transition from compartment E to compartments I_A or I_P or to the next vaccination strata within E), for conciseness, we write

$$\left(d_{E,I_A}^{i,j,k},\, d_{E,I_P}^{i,j,k},\, d_{E,v}^{i,j,k}\right)\sim \text{Multinom}\left(E^{i,j,k,2}(t),\, q_{E,I_A}^{i,j,k},\, q_{E,I_P}^{i,j,k},\, q_{E,v}^{i,j,k}\right) \tag{73}$$

instead of

$$\left(d_{E,I_A}^{i,j,k},\, d_{E,I_P}^{i,j,k},\, d_{E,v}^{i,j,k},\, d_{nomove}^{i,j,k}\right)\sim \text{Multinom}\left(E^{i,j,k,2}(t),\, q_{E,I_A}^{i,j,k},\, q_{E,I_P}^{i,j,k},\, q_{E,v}^{i,j,k},\, 1-\sum_{x\in I_A,I_P,v}q_{E,x}^{i,j,k}\right) \tag{74}$$

where $d_{nomove}^{i,j,k}$ is a dummy variable counting the number of individuals remaining in compartment $E^{i,j,k,2}$. We also omit the time dependency i.e. we use $d_{E,I_A}^{i,j,k}$ or $q_{E,I_A}^{i,j,k}$ instead of $d_{E,I_A}^{i,j,k}(t)$ or $q_{E,I_A}^{i,j,k}(t)$.

Using this convention, transition variables are drawn from the following distributions, with probabilities defined below:

$$q_{S,E}^{i,Alpha,k} = \left(1 - e^{-\Lambda^{i,k}(t)dt}\right) \frac{\lambda^{i,Alpha,k}(t)}{\Lambda^{i,k}(t)} \tag{75}$$

$$q_{S,E}^{i,Delta,k} = \left(1 - e^{-\Lambda^{i,k}(t)dt}\right) \frac{\lambda^{i,Delta,k}(t)}{\Lambda^{i,k}(t)} \tag{76}$$

$$q_{S,v}^{i,k} = e^{-\Lambda^{i,k}(t)dt}\left(1 - e^{-\zeta^{i,k}(t)dt}\right) \tag{77}$$

$$\left(d_{S,E}^{i,Alpha,k},\, d_{S,E}^{i,Delta,k},\, d_{S,v}^{i,k}\right) \sim \text{Multinom}\left(S^{i,k}(t),\, q_{S,E}^{i,Alpha,k},\, q_{S,E}^{i,Delta,k},\, q_{S,v}^{i,k}\right) \tag{78}$$

$$\left(q_{E,E}^{i,j,k},\, q_{E,v}^{i,j,k,1}\right) = \left(1 - e^{-\gamma_E dt},\, e^{-\gamma_E dt}\left(1 - e^{-\zeta^{i,k}(t)dt}\right)\right) \tag{79}$$

$$\left(d_{E,E}^{i,j,k},\, d_{E,v}^{i,j,k,1}\right) \sim \text{Multinom}\left(E^{i,j,k,1}(t),\, q_{E,E}^{i,j,k},\, q_{E,v}^{i,j,k,1}\right) \tag{80}$$

$$q_{E,I_A}^{i,j,k} = \left(1 - p_C^{i,j,k}\right)\left(1 - e^{-\gamma_E dt}\right) \tag{81}$$

$$q_{E,I_P}^{i,j,k} = p_C^{i,j,k}\left(1 - e^{-\gamma_E dt}\right) \tag{82}$$

$$q_{E,v}^{i,j,k,2} = e^{-\gamma_E dt}\left(1 - e^{-\zeta^{i,k}(t)dt}\right) \tag{83}$$

$$\left(d_{E,I_A}^{i},\, d_{E,I_P}^{i},\, d_{E,v}^{i,j,k,2}\right) \sim \text{Multinom}\left(E^{i,j,k,2}(t),\, q_{E,I_A}^{i,j,k},\, q_{E,I_P}^{i,j,k},\, q_{E,v}^{i,j,k,2}\right) \tag{84}$$

$$\left(q_{I_A,R}^{i,j,k},\, q_{I_A,v}^{i,j,k}\right) = \left(1 - e^{-\gamma_A dt},\, e^{-\gamma_A dt}\left(1 - e^{-\zeta^{i,k}(t)dt}\right)\right) \tag{85}$$

$$\left(d_{I_A,R}^{i,j,k},\, d_{I_A,v}^{i,j,k}\right) \sim \text{Multinom}\left(I_A^i(t),\, q_{I_A,R}^{i,j,k},\, q_{I_A,v}^{i,j,k}\right) \tag{86}$$

$$\left(q_{I_P,I_{C_1}}^{i,j,k},\, q_{I_P,v}^{i,j,k}\right) = \left(1 - e^{-\gamma_P dt},\, e^{-\gamma_P dt}\left(1 - e^{-\zeta^{i,k}(t)dt}\right)\right) \tag{87}$$

$$\left(d_{I_P,I_{C_1}}^{i,j,k},\, d_{I_P,v}^{i,j,k}\right) \sim \text{Multinom}\left(I_P^i(t),\, q_{I_P,I_{C_1}}^{i,j,k},\, q_{I_P,v}^{i,j,k}\right) \tag{88}$$

$$d_{I_{C_1},I_{C_2}}^{i,j,k} \sim \text{Binom}\left(I_{C_1}^{i,j,k}(t),\, 1 - e^{-\gamma_{C_1} dt}\right) \tag{89}$$

$$q_{I_{C_2},G_D}^{i,j,k} = p_H^{i,j,k}(t) p_{G_D}^{i,j,k}\left(1 - e^{-\gamma_{C_2} dt}\right) \tag{90}$$

$$q_{I_{C_2},R}^{i,j,k} = \left(1 - p_H^{i,j,k}(t)\right)\left(1 - e^{-\gamma_{C_2} dt}\right) \tag{91}$$

$$q_{I_{C_2},ICU_{pre}}^{i,j,k} = p_H^{i,j,k}(t)\left(1 - p_{G_D}^{i,j,k}\right)\left(1 - p^*(t)\right) p_{ICU}^i(t)\left(1 - e^{-\gamma_{C_2} dt}\right) \tag{92}$$

$$q_{I_{C_2},ICU_{pre*}}^{i,j,k} = p_H^{i,j,k}(t)\left(1 - p_{G_D}^{i,j,k}\right) p^*(t) p_{ICU}^i(t)\left(1 - e^{-\gamma_{C_2} dt}\right) \tag{93}$$

$$q_{I_{C_2},H_R}^{i,j,k} = p_H^{i,j,k}(t)\left(1 - p_{G_D}^{i,j,k}\right)\left(1 - p^*(t)\right)\left(1 - p_{ICU}^i(t)\right)\left(1 - p_{H_D}^{i,j,k}(t)\right)\left(1 - e^{-\gamma_{C_2} dt}\right) \tag{94}$$

$$q_{I_{C_2},H_{R*}}^{i,j,k} = p_H^{i,j,k}(t)\left(1 - p_{G_D}^{i,j,k}\right) p^*(t)\left(1 - p_{ICU}^i(t)\right)\left(1 - p_{H_D}^{i,j,k}(t)\right)\left(1 - e^{-\gamma_{C_2} dt}\right) \tag{95}$$

$$q^{i,j,k}_{I_{C_2},H_D} = p^{i,j,k}_{H}(t)\left(1-p^{i,j,k}_{G_D}\right)(1-p^*(t))\left(1-p^{i}_{ICU}(t)\right)p^{i,j,k}_{H_D}(t)\left(1-e^{-\gamma_{C_2}dt}\right) \tag{96}$$

$$q^{i,j,k}_{I_{C_2},H_{D^*}} = p^{i,j,k}_{H}(t)\left(1-p^{i,j,k}_{G_D}\right)p^*(t)\left(1-p^{i}_{ICU}(t)\right)p^{i,j,k}_{H_D}(t)\left(1-e^{-\gamma_{C_2}dt}\right) \tag{97}$$

$$\left(d^{i,j,k}_{I_{C_2},G_D},\dots,d^{i,j,k}_{I_{C_2},H_{D^*}}\right) \sim \text{Multinom}\left(I^{i,j,k}_{C_2}(t),\ q^{i,j,k}_{I_{C_2},G_D},\dots,q^{i,j,k}_{I_{C_2},H_{D^*}}\right) \tag{98}$$

$$d^{i,j,k}_{G_D,G_D} \sim \text{Binom}\left(G^{i,j,k,1}_{D}(t),\ 1-e^{-\gamma_{G_D}dt}\right) \tag{99}$$

$$d^{i,j,k}_{G_D,D} \sim \text{Binom}\left(G^{i,j,k,2}_{D}(t),\ 1-e^{-\gamma_{G_D}dt}\right) \tag{100}$$

$$q^{i,j,k}_{ICU_{pre},ICU_{W_R}} = \left(1-p^{i,j,k}_{ICU_D}(t)\right)\left(1-p^{i,j,k}_{W_D}(t)\right)\left(1-e^{-\gamma_{ICU_{pre}}dt}\right)e^{-\gamma_U dt} \tag{101}$$

$$q^{i,j,k}_{ICU_{pre},ICU_{W_{R^*}}} = \left(1-p^{i,j,k}_{ICU_D}(t)\right)\left(1-p^{i,j,k}_{W_D}(t)\right)\left(1-e^{-\gamma_{ICU_{pre}}dt}\right)\left(1-e^{-\gamma_U dt}\right) \tag{102}$$

$$q^{i,j,k}_{ICU_{pre},ICU_{W_D}} = \left(1-p^{i,j,k}_{ICU_D}(t)\right)p^{i,j,k}_{W_D}(t)\left(1-e^{-\gamma_{ICU_{pre}}dt}\right)e^{-\gamma_U dt} \tag{103}$$

$$q^{i,j,k}_{ICU_{pre},ICU_{W_{D^*}}} = \left(1-p^{i,j,k}_{ICU_D}(t)\right)p^{i,j,k}_{W_D}(t)\left(1-e^{-\gamma_{ICU_{pre}}dt}\right)\left(1-e^{-\gamma_U dt}\right) \tag{104}$$

$$q^{i,j,k}_{ICU_{pre},ICU_D} = p^{i,j,k}_{ICU_D}(t)\left(1-e^{-\gamma_{ICU_{pre}}dt}\right)e^{-\gamma_U dt} \tag{105}$$

$$q^{i,j,k}_{ICU_{pre},ICU_{D^*}} = p^{i,j,k}_{ICU_D}(t)\left(1-e^{-\gamma_{ICU_{pre}}dt}\right)\left(1-e^{-\gamma_U dt}\right) \tag{106}$$

$$q^{i,j,k}_{ICU_{pre},ICU_{pre^*}} = e^{-\gamma_{ICU_{pre}}dt}\left(1-e^{-\gamma_U dt}\right) \tag{107}$$

$$\begin{aligned}&\left(d^{i,j,k}_{ICU_{pre},ICU_{W_R}},\ \dots,d^{i,j,k}_{ICU_{pre},ICU_{pre^*}}\right)\\ &\quad\sim \text{Multinom}\left(ICU^{i,j,k}_{pre}(t),\ q^{i,j,k}_{ICU_{pre},ICU_{W_R}},\dots,q^{i,j,k}_{ICU_{pre},ICU_{pre^*}}\right)\end{aligned} \tag{108}$$

$$q^{i,j,k}_{ICU_{pre^*},ICU_{W_{R^*}}} = \left(1-p^{i,j,k}_{ICU_D}(t)\right)\left(1-p^{i,j,k}_{W_D}(t)\right)\left(1-e^{-\gamma_{ICU_{pre}}dt}\right) \tag{109}$$

$$q^{i,j,k}_{ICU_{pre^*},ICU_{W_{D^*}}} = \left(1-p^{i,j,k}_{ICU_D}(t)\right)p^{i,j,k}_{W_D}(t)\left(1-e^{-\gamma_{ICU_{pre}}dt}\right) \tag{110}$$

$$q^{i,j,k}_{ICU_{pre^*},ICU_{D^*}} = p^{i,j,k}_{ICU_D}(t)\left(1-e^{-\gamma_{ICU_{pre}}dt}\right) \tag{111}$$

$$\begin{aligned}&\left(d^{i,j,k}_{ICU_{pre^*},ICU_{W_{R^*}}},\ \dots,\ d^{i,j,k}_{ICU_{pre^*},ICU_{D^*}}\right)\\ &\quad\sim \text{Multinom}\left(ICU^{i,j,k}_{pre^*}(t),\ q^{i,j,k}_{ICU_{pre^*},ICU_{W_{R^*}}},\dots,q^{i,j,k}_{ICU_{pre^*},ICU_{D^*}}\right)\end{aligned} \tag{112}$$

$$q^{i,j,k}_{H_D,H_D} = \left(1-e^{-\gamma_{H_D}(t)dt}\right)e^{-\gamma_U dt} \tag{113}$$

$$q^{i,j,k,1,1}_{H_D,H_{D^*}} = e^{-\gamma_{H_D}(t)dt}\left(1-e^{-\gamma_U dt}\right) \tag{114}$$

$$q^{i,j,k,1,2}_{H_D,H_{D^*}} = \left(1-e^{-\gamma_{H_D}(t)dt}\right)\left(1-e^{-\gamma_U dt}\right) \tag{115}$$

$$\begin{aligned}&\left(d^{i,j,k}_{H_D,H_D},\ \ d^{i,j,k,1,1}_{H_D,H_{D^*}},\ d^{i,j,k,1,2}_{H_D,H_{D^*}}\right)\\ &\quad\sim \text{Multinom}\left(H^{i,j,k,1}_{D}(t),\ q^{i,j,k}_{H_D,H_D},\ q^{i,j,k,1,1}_{H_D,H_{D^*}},\ q^{i,j,k,1,2}_{H_D,H_{D^*}}\right)\end{aligned} \tag{116}$$

$$d^{i,j,k}_{H_{D^*},H_{D^*}} \sim \text{Binom}\left(H^{i,j,k,1}_{D^*}(t),\ 1-e^{-\gamma_{H_D}(t)dt}\right) \tag{117}$$

$$\left(d^{i,j,k}_{H_D,D},\ d^{i,j,k,2,2}_{H_D,H_{D^*}}\right) \sim \text{Multinom}\left(H^{i,j,k,2}_{D}(t),\ 1-e^{-\gamma_{H_D}(t)dt},\ e^{-\gamma_{H_D}(t)dt}\left(1-e^{-\gamma_U dt}\right)\right) \tag{118}$$

$$d^{i,j,k}_{H_{D^*},D} \sim \text{Binom}\left(H^{i,j,k,2}_{D^*}(t),\ 1-e^{-\gamma_{H_D}(t)dt}\right) \tag{119}$$

$$\left(d^{i,j,k}_{H_R,R},\ d^{i,j,k}_{H_R,H_{R^*}}\right) \sim \text{Multinom}\left(H^{i,j,k}_R(t),\ 1-e^{-\gamma_{H_R}(t)dt},\ e^{-\gamma_{H_R}(t)dt}\left(1-e^{-\gamma_U dt}\right)\right) \tag{120}$$

$$d^{i,j,k}_{H_{R^*},R} \sim \text{Binom}\left(H^{i,j,k}_{R^*}(t),\ 1-e^{-\gamma_{H_R}(t)dt}\right) \tag{121}$$

$$q^{i,j,k}_{ICU_{W_R},W_R} = \left(1-e^{-\gamma_{ICU_{W_R}}dt}\right)e^{-\gamma_U dt} \tag{122}$$

$$q^{i,j,k}_{ICU_{W_R},ICU_{W_{R^*}}} = e^{-\gamma_{ICU_{W_R}}dt}\left(1-e^{-\gamma_U dt}\right) \tag{123}$$

$$q^{i,j,k}_{ICU_{W_R},W_{R^*}} = \left(1-e^{-\gamma_{ICU_{W_R}}dt}\right)\left(1-e^{-\gamma_U dt}\right) \tag{124}$$

$$\begin{aligned}\left(d^{i,j,k}_{ICU_{W_R},W_R},\ \ldots, d^{i,j,k}_{ICU_{W_R},W_{R^*}}\right)\\ \sim \text{Multinom}\left(ICU^{i,j,k}_{W_R}(t),\ q^{i,j,k}_{ICU_{W_R},W_R},\ldots,q^{i,j,k}_{ICU_{W_R},W_{R^*}}\right)\end{aligned} \tag{125}$$

$$d^{i,j,k}_{ICU_{W_{R^*}},W_{R^*}} \sim \text{Binom}\left(ICU^{i,j,k}_{W_{R^*}}(t),\ 1-e^{-\gamma_{ICU_{W_R}}dt}\right) \tag{126}$$

$$q^{i,j,k}_{ICU_{W_D},W_D} = \left(1-e^{-\gamma_{ICU_{W_D}}dt}\right)e^{-\gamma_U dt} \tag{127}$$

$$q^{i,j,k}_{ICU_{W_D},ICU_{W_{D^*}}} = e^{-\gamma_{ICU_{W_D}}dt}\left(1-e^{-\gamma_U dt}\right) \tag{128}$$

$$q^{i,j,k}_{ICU_{W_D},W_{D^*}} = \left(1-e^{-\gamma_{ICU_{W_D}}dt}\right)\left(1-e^{-\gamma_U dt}\right) \tag{129}$$

$$\begin{aligned}\left(d^{i,j,k}_{ICU_{W_D},W_D},\ \ldots, d^{i,j,k}_{ICU_{W_D},W_{D^*}}\right)\\ \sim \text{Multinom}\left(ICU^{i,j,k}_{W_D}(t),\ q^{i,j,k}_{ICU_{W_D},W_D},\ldots,q^{i,j,k}_{ICU_{W_D},W_{D^*}}\right)\end{aligned} \tag{130}$$

$$d^{i,j,k}_{ICU_{W_{D^*}},W_{D^*}} \sim \text{Binom}\left(ICU^{i,j,k}_{W_{D^*}}(t),\ 1-e^{-\gamma_{ICU_{W_D}}dt}\right) \tag{131}$$

$$q^{i,j,k}_{ICU_D,ICU_D} = \left(1-e^{-\gamma_{ICU_D}dt}\right)e^{-\gamma_U dt} \tag{132}$$

$$q^{i,j,k,1,1}_{ICU_D,ICU_{D^*}} = e^{-\gamma_{ICU_D}dt}\left(1-e^{-\gamma_U dt}\right) \tag{133}$$

$$q^{i,j,k,1,2}_{ICU_D,ICU_{D^*}} = \left(1-e^{-\gamma_{ICU_D}dt}\right)\left(1-e^{-\gamma_U dt}\right) \tag{134}$$

$$\begin{aligned}\left(d^{i,j,k}_{ICU_D,ICU_D},\ d^{i,j,k,1,1}_{ICU_D,ICU_{D^*}},\ d^{i,j,k,1,2}_{ICU_D,ICU_{D^*}}\right)\\ \sim \text{Multinom}\left(ICU^{i,j,k,1}_D(t),\ q^{i,j,k}_{ICU_D,ICU_D},\ q^{i,j,k,1,1}_{ICU_D,ICU_{D^*}},\ q^{i,j,k,1,2}_{ICU_D,ICU_{D^*}}\right)\end{aligned} \tag{135}$$

$$d^{i,j,k}_{ICU_{D^*},ICU_{D^*}} \sim \text{Binom}\left(ICU^{i,j,k,1}_{D^*}(t),\ 1-e^{-\gamma_{ICU_D}dt}\right) \tag{136}$$

$$\left(q^{i,j,k}_{ICU_D,D},\ q^{i,j,k,2,2}_{ICU_D,ICU_{D^*}}\right) = \left(\ 1-e^{-\gamma_{ICU_D}dt},\ e^{-\gamma_{ICU_D}dt}\left(1-e^{-\gamma_U dt}\right)\right) \tag{137}$$

$$\left(d^{i,j,k}_{ICU_D,D},\ d^{i,j,k,2,2}_{ICU_D,ICU_{D^*}}\right) \sim \text{Multinom}\left(ICU^{i,j,k,2}_D(t),\ q^{i,j,k}_{ICU_D,D},\ q^{i,j,k,2,2}_{ICU_D,ICU_{D^*}}\right) \tag{138}$$

$$d^{i,j,k}_{ICU_{D^*},D} \sim \text{Binom}\left(ICU^{i,j,k,2}_{D^*}(t),\ 1-e^{-\gamma_{ICU_D}dt}\right) \tag{139}$$

$$q^{i,j,k}_{W_R,W_R} = \left(1-e^{-\gamma_{W_R}(t)dt}\right)e^{-\gamma_U dt} \tag{140}$$

$$q^{i,j,k,1,1}_{W_R,W_{R^*}} = e^{-\gamma_{W_R}(t)dt}\left(1-e^{-\gamma_U dt}\right) \tag{141}$$

$$q_{W_R,W_{R^*}}^{i,j,k,1,2} = \left(1 - e^{-\gamma_{W_R}(t)dt}\right)\left(1 - e^{-\gamma_U dt}\right) \tag{142}$$

$$\begin{aligned}&\left(d_{W_R,W_R}^{i,j,k},\ d_{W_R,W_{R^*}}^{i,j,k,1,1},\ d_{W_R,W_{R^*}}^{i,j,k,1,2}\right) \\ &\qquad \sim \text{Multinom}\left(W_R^{i,j,k,1}(t),\ q_{W_R,W_R}^{i,j,k},\ q_{W_R,W_{R^*}}^{i,j,k,1,1},\ q_{W_R,W_{R^*}}^{i,j,k,1,2}\right)\end{aligned} \tag{143}$$

$$d_{W_{R^*},W_{R^*}}^{i,j,k} \sim \text{Binom}\left(W_{R^*}^{i,j,k,1}(t),\ 1 - e^{-\gamma_{W_R}(t)dt}\right) \tag{144}$$

$$\left(q_{W_R,R}^{i,j,k},\ q_{W_R,W_{R^*}}^{i,j,k,2,2}\right) = \left(1 - e^{-\gamma_{W_R}(t)dt},\ e^{-\gamma_{W_R}(t)dt}\left(1 - e^{-\gamma_U\ dt}\right)\right) \tag{145}$$

$$\left(d_{W_R,R}^{i,j,k},\ d_{W_R,W_{R^*}}^{i,j,k,2,2}\right) \sim \text{Multinom}\left(W_R^{i,j,k,2}(t),\ q_{W_R,R}^{i,j,k},\ q_{W_R,W_{R^*}}^{i,j,k,2,2}\right) \tag{146}$$

$$d_{W_{R^*},R}^{i,j,k} \sim \text{Binom}\left(W_{R^*}^{i,j,k,2}(t),\ 1 - e^{-\gamma_{W_R}(t)dt}\right) \tag{147}$$

$$\left(q_{W_D,D}^{i,j,k},\ q_{W_D,W_{D^*}}^{i,j,k}\right) = \left(1 - e^{-\gamma_{W_D}(t)dt},\ e^{-\gamma_{W_D}(t)dt}\left(1 - e^{-\gamma_U dt}\right)\right) \tag{148}$$

$$\left(d_{W_D,D}^{i,j,k},\ d_{W_D,W_{D^*}}^{i,j,k}\right) \sim \text{Multinom}\left(W_D^{i,j,k}(t),\ q_{W_D,D}^{i,j,k},\ q_{W_D,W_{D^*}}^{i,j,k}\right) \tag{149}$$

$$d_{W_{D^*},D}^{i,j,k} \sim \text{Binom}\left(W_{D^*}^{i,j,k}(t),\ 1 - e^{-\gamma_{W_D}(t)dt}\right) \tag{150}$$

$$\gamma_{R,E}^{i,j,k} = \mathbb{1}_{\{Alpha\}}(j)(1-\eta)\lambda^{i,Delta,k}(t) \tag{151}$$

$$q_{R,S}^{i,j,k} = \left(1 - e^{-\left(\gamma_R + \gamma_{R,E}^{i,j,k}\right)dt}\right)\frac{\gamma_R}{\gamma_R + \gamma_{R,E}^{i,j,k}} \tag{152}$$

$$q_{R,E}^{i,j,k} = \left(1 - e^{-\left(\gamma_R + \gamma_{R,E}^{i,j,k}\right)dt}\right)\frac{\gamma_{R,E}^{i,j,k}}{\gamma_R + \gamma_{R,E}^{i,j,k}} \tag{153}$$

$$q_{R,v}^{i,j,k} = e^{-\left(\gamma_R + \gamma_{R,E}^{i,j,k}\right)dt}\left(1 - e^{-\zeta^{i,k}(t)dt}\right) \tag{154}$$

$$\left(d_{R,S}^{i,j,k},\ d_{R,E}^{i,j,k},\ d_{R,v}^{i,j,k}\right) \sim \text{Multinom}\left(R^{i,j,k}(t),\ q_{R,S}^{i,j,k},\ q_{R,E}^{i,j,k},\ q_{R,v}^{i,j,k}\right) \tag{155}$$

$$q^i_{T_{sero^1_{pre}},T_{sero^1_{pos}}} = p_{sero_{pos}}\left(1 - e^{-\gamma_{sero_{pre}}dt}\right) \tag{156}$$

$$q^i_{T_{sero^1_{pre}},T_{sero^1_{neg}}} = \left(1 - p_{sero_{pos}}\right)\left(1 - e^{-\gamma_{sero_{pre}}dt}\right) \tag{157}$$

$$\begin{aligned}&\left(d^i_{T_{sero^1_{pre}},T_{sero^1_{pos}}},\ d^i_{T_{sero^1_{pre}},T_{sero^1_{neg}}}\right) \\ &\qquad \sim \text{Multinom}\left(T^i_{sero^1_{pre}}(t),\ q^i_{T_{sero^1_{pre}},T_{sero^1_{pos}}},\ q^i_{T_{sero^1_{pre}},T_{sero^1_{neg}}}\right)\end{aligned} \tag{158}$$

$$d^i_{T_{sero^1_{pos}},T_{sero^1_{neg}}} \sim \text{Binom}\left(T^i_{sero^1_{pos}}(t),\ 1 - e^{-\gamma_{sero^1_{pos}}dt}\right) \tag{159}$$

$$q^i_{T_{sero^2_{pre}},T_{sero^2_{pos}}} = p_{sero_{pos}}\left(1 - e^{-\gamma_{sero_{pre}}dt}\right) \tag{160}$$

$$q^i_{T_{sero^2_{pre}},T_{sero^2_{neg}}} = \left(1 - p_{sero_{pos}}\right)\left(1 - e^{-\gamma_{sero_{pre}}dt}\right) \tag{161}$$

$$\begin{aligned}&\left(d^i_{T_{sero^2_{pre}},T_{sero^2_{pos}}},\ d^i_{T_{sero^2_{pre}},T_{sero^2_{neg}}}\right) \\ &\qquad \sim \text{Multinom}\left(T^i_{sero^2_{pre}}(t),\ q^i_{T_{sero^2_{pre}},T_{sero^2_{pos}}},\ q^i_{T_{sero^2_{pre}},T_{sero^2_{neg}}}\right)\end{aligned} \tag{162}$$

$$d^i_{T_{sero^2_{pos}},T_{sero^2_{neg}}} \sim \text{Binom}\left(T^i_{sero^2_{pos}}(t),\ 1-e^{-\gamma_{sero^2_{pos}}dt}\right) \tag{163}$$

$$d^i_{T_{PCR_{pre}},T_{PCR_{pos}}} \sim \text{Binom}\left(T^i_{PCR_{pre}}(t),\ 1-e^{-\gamma_{PCR_{pre}}dt}\right) \tag{164}$$

$$d^i_{T_{PCR_{pos}},T_{PCR_{neg}}} \sim \text{Binom}\left(T^i_{PCR_{pos}}(t),\ 1-e^{-\gamma_{PCR_{pos}}dt}\right) \tag{165}$$

Model compartments were then updated as follows (Note that $d^{i,Alpha\to Delta,k}_{S,E} = 0$):

$$S^{i,k}(t+dt) := S^{i,k}(t) - d^{i,Alpha,k}_{S,E} - d^{i,Delta,k}_{S,E} + d^{i,j,k}_{R,S} + d^{i,k-1}_{S,v} - d^{i,k}_{S,v} \tag{166}$$

$$E^{i,j,k,1}(t+dt) := E^{i,j,k,1}(t) + d^{i,j,k}_{S,E} + \mathbb{1}_{\{Alpha\to Delta\}}(j)d^{i,Alpha,k}_{R,E} - d^{i,j,k}_{E,E} + d^{i,j,k-1,1}_{E,v} - d^{i,j,k,1}_{E,v} \tag{167}$$

$$E^{i,j,k,2}(t+dt) := E^{i,j,k,2}(t) + d^{i,j,k}_{E,E} - d^{i,j,k}_{E,I_A} - d^{i,j,k}_{E,I_P} + d^{i,j,k-1,2}_{E,v} - d^{i,j,k,2}_{E,v} \tag{168}$$

$$I^{i,j,k}_A(t+dt) := I^{i,j,k}_A(t) + d^{i,j,k}_{E,I_A} - d^{i,j,k}_{I_A,R} + d^{i,j,k-1}_{I_A,v} - d^{i,j,k}_{I_A,v} \tag{169}$$

$$I^{i,j,k}_P(t+dt) := I^{i,j,k}_P(t) + d^{i,j,k}_{E,I_P} - d^{i,j,k}_{I_P,I_{C_1}} + d^{i,j,k-1}_{I_P,v} - d^{i,j,k}_{I_P,v} \tag{170}$$

$$I^{i,j,k}_{C_1}(t+dt) := I^{i,j,k}_{C_1}(t) + d^{i,j,k}_{I_P,I_{C_1}} - d^{i,j,k}_{I_{C_1},I_{C_2}} \tag{171}$$

$$\begin{aligned} I^{i,j,k}_{C_2}(t+dt) := {} & I^{i,j,k}_{C_2}(t) + d^{i,j,k}_{I_{C_1},I_{C_2}} - d^{i,j,k}_{I_{C_2},G_D} - d^{i,j,k}_{I_{C_2},R} - d^{i,j,k}_{I_{C_2},ICU_{pre}} \\ & - d^{i,j,k}_{I_{C_2},ICU_{pre^*}} - d^{i,j,k}_{I_{C_2},H_R} - d^{i,j,k}_{I_{C_2},H_{R^*}} - d^{i,j,k}_{I_{C_2},H_D} - d^{i,j,k}_{I_{C_2},H_{D^*}} \end{aligned} \tag{172}$$

$$G^{i,j,k,1}_D(t+dt) := G^{i,j,k,1}_D(t) + d^{i,j,k}_{I_{C_2},G_D} - d^{i,j,k}_{G_D,G_D} \tag{173}$$

$$G^{i,j,k,2}_D(t+dt) := G^{i,j,k,2}_D(t) + d^{i,j,k}_{G_D,G_D} - d^{i,j,k}_{G_D,D} \tag{174}$$

$$\begin{aligned} ICU^{i,j,k}_{pre}(t+dt) := {} & ICU^{i,j,k}_{pre}(t) + d^{i,j,k}_{I_{C_2},ICU_{pre}} - d^{i,j,k}_{ICU_{pre},ICU_{W_R}} - d^{i,j,k}_{ICU_{pre},ICU_{W_D}} \\ & - d^{i,j,k}_{ICU_{pre},ICU_D} - d^{i,j,k}_{ICU_{pre},ICU_{pre^*}} - d^{i,j,k}_{ICU_{pre},ICU_{W_{R^*}}} - d^{i,j,k}_{ICU_{pre},ICU_{W_{D^*}}} \\ & - d^{i,j,k}_{ICU_{pre},ICU_{D^*}} \end{aligned} \tag{175}$$

$$\begin{aligned} ICU^{i,j,k}_{pre^*}(t+dt) := {} & ICU^{i,j,k}_{pre^*}(t) + d^{i,j,k}_{I_{C_2},ICU_{pre^*}} - d^{i,j,k}_{ICU_{pre},ICU_{W_{D^*}}} - d^{i,j,k}_{ICU_{pre^*},ICU_{W_{R^*}}} \\ & - d^{i,j,k}_{ICU_{pre^*},ICU_{D^*}} \end{aligned} \tag{176}$$

$$\begin{aligned} ICU^{i,j,k}_{W_R}(t+dt) := {} & ICU^{i,j,k}_{W_R}(t) + d^{i,j,k}_{ICU_{pre},ICU_{W_R}} - d^{i,j,k}_{ICU_{W_R},W_R} - d^{i,j,k}_{ICU_{W_R},ICU_{W_{R^*}}} \\ & - d^{i,j,k}_{ICU_{W_R},W_{R^*}} \end{aligned} \tag{177}$$

$$\begin{aligned} ICU^{i,j,k}_{W_{R^*}}(t+dt) := {} & ICU^{i,j,k}_{W_{R^*}}(t) + d^{i,j,k}_{ICU_{pre^*},ICU_{W_{R^*}}} + d^{i,j,k}_{ICU_{W_R},ICU_{W_{R^*}}} + d^{i,j,k}_{ICU_{pre},ICU_{W_{R^*}}} \\ & - d^{i,j,k}_{ICU_{W_{R^*}},W_{R^*}} \end{aligned} \tag{178}$$

$$\begin{aligned} ICU^{i,j,k}_{W_D}(t+dt) := {} & ICU^{i,j,k}_{W_D}(t) + d^{i,j,k}_{ICU_{pre},ICU_{W_D}} - d^{i,j,k}_{ICU_{W_D},W_D} - d^{i,j,k}_{ICU_{W_D},ICU_{W_{D^*}}} \\ & - d^{i,j,k}_{ICU_{W_D},W_{D^*}} \end{aligned} \tag{179}$$

$$ICU_{W_{D^*}}^{i,j,k}(t+dt) := ICU_{W_{D^*}}^{i,j,k}(t) + d_{ICU_{pre^*},ICU_{W_{D^*}}}^{i,j,k} + d_{ICU_{W_D},ICU_{W_{D^*}}}^{i,j,k} + d_{ICU_{pre},ICU_{W_{D^*}}}^{i,j,k} - d_{ICU_{W_{D^*}},W_{D^*}}^{i,j,k} \tag{180}$$

$$ICU_D^{i,j,k,1}(t+dt) := ICU_D^{i,j,k,1}(t) + d_{ICU_{pre},ICU_D}^{i,j,k} - d_{ICU_D,ICU_D}^{i,j,k} - d_{ICU_D,ICU_{D^*}}^{i,j,k,1,1} - d_{ICU_D,ICU_{D^*}}^{i,j,k,1,2} \tag{181}$$

$$ICU_D^{i,j,k,2}(t+dt) := ICU_D^{i,j,k,2}(t) + d_{ICU_D,ICU_D}^{i,j,k} - d_{ICU_D,D}^{i,j,k} - d_{ICU_D,ICU_{D^*}}^{i,j,k,2,2} \tag{182}$$

$$ICU_{D^*}^{i,j,k,1}(t+dt) := ICU_{D^*}^{i,j,k,1}(t) + d_{ICU_{pre^*},ICU_{D^*}}^{i,j,k} + d_{ICU_D,ICU_{D^*}}^{i,j,k,1,1} + d_{ICU_{pre},ICU_{D^*}}^{i,j,k} - d_{ICU_{D^*},ICU_{D^*}}^{i,j,k} \tag{183}$$

$$ICU_{D^*}^{i,j,k,2}(t+dt) := ICU_{D^*}^{i,j,k,2}(t) + d_{ICU_{D^*},ICU_{D^*}}^{i,j,k} + d_{ICU_D,ICU_{D^*}}^{i,j,k,1,2} + d_{ICU_D,ICU_{D^*}}^{i,j,k,2,2} - d_{ICU_{D^*},D}^{i,j,k} \tag{184}$$

$$W_R^{i,j,k,1}(t+dt) := W_R^{i,j,k,1}(t) + d_{ICU_{W_R},W_R}^{i,j,k} - d_{W_R,W_R}^{i,j,k} - d_{W_R,W_{R^*}}^{i,j,k,1,1} - d_{W_R,W_{R^*}}^{i,j,k,1,2} \tag{185}$$

$$W_R^{i,j,k,2}(t+dt) := W_R^{i,j,k,2}(t) + d_{W_R,W_R}^{i,j,k} - d_{W_R,R}^{i,j,k} - d_{W_R,W_{R^*}}^{i,j,k,2,2} \tag{186}$$

$$W_{R^*}^{i,j,k,1}(t+dt) := W_{R^*}^{i,j,k,1}(t) + d_{ICU_{W_{R^*}},W_{R^*}}^{i,j,k} + d_{W_R,W_{R^*}}^{i,j,k,1,1} + d_{ICU_{W_R},W_{R^*}}^{i,j,k} - d_{W_{R^*},W_{R^*}}^{i,j,k} \tag{187}$$

$$W_{R^*}^{i,j,k,2}(t+dt) := W_{R^*}^{i,j,k,2}(t) + d_{W_{R^*},W_{R^*}}^{i,j,k} + d_{W_R,W_{R^*}}^{i,j,k,2,2} + d_{W_R,W_{R^*}}^{i,j,k,1,2} - d_{W_{R^*},R}^{i,j,k} \tag{188}$$

$$W_D^{i,j,k}(t+dt) := W_D^{i,j,k}(t) + d_{ICU_{W_D},W_D}^{i,j,k} - d_{W_D,D}^{i,j,k} - d_{W_D,W_{D^*}}^{i,j,k} \tag{189}$$

$$W_{D^*}^{i,j,k}(t+dt) := W_{D^*}^{i,j,k}(t) + d_{ICU_{W_{D^*}},W_{D^*}}^{i,j,k} + d_{W_D,W_{D^*}}^{i,j,k} + d_{ICU_{W_D},W_{D^*}}^{i,j,k} - d_{W_{D^*},D}^{i,j,k} \tag{190}$$

$$H_D^{i,j,k,1}(t+dt) := H_D^{i,j,k,1}(t) + d_{I_{C_2},H_D}^{i,j,k} - d_{H_D,H_D}^{i,j,k} - d_{H_D,H_{D^*}}^{i,j,k,1,1} - d_{H_D,H_{D^*}}^{i,j,k,1,2} \tag{191}$$

$$H_D^{i,j,k,2}(t+dt) := H_D^{i,j,k,2}(t) + d_{H_D,H_D}^{i,j,k} - d_{H_D,D}^{i,j,k} - d_{H_D,H_{D^*}}^{i,j,k,2,2} \tag{192}$$

$$H_{D^*}^{i,j,k,1}(t+dt) := H_{D^*}^{i,j,k,1}(t) + d_{I_{C_2},H_{D^*}}^{i,j,k} + d_{H_D,H_{D^*}}^{i,j,k,1,1} - d_{H_{D^*},H_{D^*}}^{i,j,k} \tag{193}$$

$$H_{D^*}^{i,j,k,2}(t+dt) := H_{D^*}^{i,j,k,2}(t) + d_{H_{D^*},H_{D^*}}^{i,j,k} + d_{H_D,H_{D^*}}^{i,j,k,2,2} + d_{H_D,H_{D^*}}^{i,j,k,1,2} - d_{H_{D^*},D}^{i,j,k} \tag{194}$$

$$H_R^{i,j,k}(t+dt) := H_R^{i,j,k}(t) + d_{I_{C_2},H_R}^{i,j,k} - d_{H_R,R}^{i,j,k} - d_{H_R,H_{R^*}}^{i,j,k} \tag{195}$$

$$H_{R^*}^{i}(t+dt) := H_{R^*}^{i,j,k}(t) + d_{I_{C_2},H_{R^*}}^{i,j,k} + d_{H_R,H_{R^*}}^{i,j,k} - d_{H_{R^*},R}^{i,j,k} \tag{196}$$

$$R^{i,j,k}(t+dt) := R^{i,j,k}(t) + d_{I_A,R}^{i,j,k} + d_{I_{C_2},R}^{i,j,k} + d_{H_R,R}^{i,j,k} + d_{H_{R^*},R}^{i,j,k} + d_{W_R,R}^{i,j,k} + d_{W_{R^*},R}^{i,j,k} - d_{R,S}^{i,j,k} - \mathbb{1}_{\{Alpha\}} d_{R,E}^{i,Alpha,k} + d_{R,v}^{i,j,k-1} - d_{R,v}^{i,j,k} \tag{197}$$

$$T_{sero_{pre}^1}^{i}(t+dt) := T_{sero_{pre}^1}^{i}(t) - d_{T_{sero_{pre}^1},T_{sero_{pos}^1}}^{i} - d_{T_{sero_{pre}^1},T_{sero_{neg}^1}}^{i} + \sum_j \sum_k d_{E,I_A}^{i,j,k} + d_{E,I_P}^{i,j,k} \tag{198}$$

$$T_{sero_{pos}^1}^{i}(t+dt) := T_{sero_{pos}^1}^{i}(t) + d_{T_{sero_{pre}^1},T_{sero_{pos}^1}}^{i} - d_{T_{sero_{pos}^1},T_{sero_{neg}^1}}^{i} \tag{199}$$

$$T_{sero_{neg}^1}^{i}(t+dt) := T_{sero_{neg}^1}^{i}(t) + d_{T_{sero_{pre}^1},T_{sero_{neg}^1}}^{i} + d_{T_{sero_{pos}^1},T_{sero_{neg}^1}}^{i} \tag{200}$$

$$T_{sero_{pre}^2}^{i}(t+dt) := T_{sero_{pre}^2}^{i}(t) - d_{T_{sero_{pre}^2},T_{sero_{pos}^2}}^{i} - d_{T_{sero_{pre}^2},T_{sero_{neg}^2}}^{i} + \sum_j \sum_k d_{E,I_A}^{i,j,k} + d_{E,I_P}^{i,j,k} \tag{201}$$

$$T_{sero_{pos}^2}^{i}(t+dt) := T_{sero_{pos}^2}^{i}(t) + d_{T_{sero_{pre}^2},T_{sero_{pos}^2}}^{i} - d_{T_{sero_{pos}^2},T_{sero_{neg}^2}}^{i} \tag{202}$$

$$T^i_{sero^2_{neg}}(t+dt) := T^i_{sero^2_{neg}}(t) + d^i_{T_{sero^2_{pre}}, T_{sero^2_{neg}}} + d^i_{T_{sero^2_{pos}}, T_{sero^2_{neg}}} \tag{203}$$

$$T^i_{PCR_{pre}}(t+dt) := T^i_{PCR_{pre}}(t) - d^i_{T_{PCR_{pre}}, T_{PCR_{pos}}} + \sum_j \sum_k d^{i,j,k}_{S,E} \tag{204}$$

$$T^i_{PCR_{pos}}(t+dt) := T^i_{PCR_{pos}}(t) + d^i_{T_{PCR_{pre}}, T_{PCR_{pos}}} - d^i_{T_{PCR_{pos}}, T_{PCR_{neg}}} \tag{205}$$

$$T^i_{PCR_{neg}}(t+dt) := T^i_{PCR_{neg}}(t) + d^i_{T_{PCR_{pos}}, T_{PCR_{neg}}} \tag{206}$$

$$\tag{207}$$

4.3 Observation process

To describe the epidemic in each NHS region, we fitted our model to time series data on hospital admissions, hospital ward occupancy (both in general beds and in ICU beds), deaths in hospitals, deaths in care homes, population serological surveys, PCR testing data and Variant and Mutation (VAM) data (see Table S1).

4.3.1 Notation for distributions used in this section

If $X \sim \text{Binom}(n, p)$, then X follows a binomial distribution with mean np and variance $np(1-p)$, such that

$$P(X = x) = P_{\text{Binom}}(x|n,\ p) = \binom{n}{x} p^x (1-p)^{(n-x)}. \tag{208}$$

If $Y \sim \text{NegBinom}(m, \kappa)$, then Y follows a negative binomial distribution with mean m and shape κ, such that

$$P(Y = y) = P_{\text{NegBinom}}(y|m, \kappa) = \frac{\Gamma(\kappa + y)}{y!\Gamma(\kappa)} \left(\frac{\kappa}{\kappa + m}\right)^{\kappa} \left(\frac{m}{\kappa + m}\right)^{y}, \tag{209}$$

where $\Gamma(x)$ is the gamma function. The variance of Y is $m + m^2/\kappa$.

If $Z \sim \text{BetaBinom}(n, \omega, \rho)$, then Z follows a beta-binomial distribution with size n, mean probability ω and overdispersion parameter ρ, such that

$$P(Z = z) = P_{\text{BetaBinom}}(z|n, \omega, \rho) = \binom{n}{z} \frac{B(z + a, n - z + b)}{B(a, b)}, \tag{210}$$

where $a = \omega \left(\frac{1-\rho}{\rho}\right)$, $b = (1-\omega)\left(\frac{1-\rho}{\rho}\right)$ and $B(a,b)$ is the beta function. The mean of Z is $n\omega$ and the variance is $n\omega(1-\omega)[1 + (n-1)\rho]$.

In the following, we use t to represent a day with observations. Note that different data streams had different sets of days with observations.

4.3.2 Hospital admissions and new diagnoses in hospital

We represented the daily number of confirmed COVID-19 hospital admissions and new diagnoses for existing hospitalised cases, $Y_{adm}(t)$, as the observed realisations of an underlying hidden Markov process, $X_{adm}(t)$, defined as:

$$
\begin{aligned}
X_{adm}\,(t) := \sum_i \sum_j \sum_k \Big(& d^{i,j,k}_{I_C,H_{R^*}} + d^{i,j,k}_{I_C,H_{D^*}} + d^{i,j,k}_{I_C,ICU_{pre^*}} + d^{i,j,k}_{H_R,H_{R^*}} + d^{i,j,k}_{ICU_{pre},ICU_{pre^*}} + d^{i,j,k}_{ICU_{W_R},ICU_{W_{R^*}}} \\
& + d^{i,j,k}_{ICU_{W_D},ICU_{W_D^*}} + d^{i,j,k}_{W_D,W_{D^*}} + d^{i,j,k,1,1}_{H_D,H_{D^*}} + d^{i,j,k,1,2}_{H_D,H_{D^*}} + d^{i,j,k,2,2}_{H_D,H_{D^*}} + d^{i,j,k,1,1}_{ICU_D,ICU_{D^*}} \\
& + d^{i,j,k,1,2}_{ICU_D,ICU_{D^*}} + d^{i,j,k,2,2}_{ICU_D,ICU_{D^*}} + d^{i,j,k,1,1}_{W_R,W_{R^*}} + d^{i,j,k,1,2}_{W_R,W_{R^*}} + d^{i,j,k,2,2}_{W_R,W_{R^*}} + d^{i,j,k}_{ICU_{pre},ICU_{W_{R^*}}} \\
& + d^{i,j,k}_{ICU_{pre},ICU_{W_{D^*}}} + d^{i,j,k}_{ICU_{pre},ICU_{D^*}} + d^{i,j,k}_{ICU_{W_{D^*}},W_{D^*}} + d^{i,j,k}_{ICU_{W_{R^*}},W_{R^*}} \Big)
\end{aligned}
\tag{211}
$$

which was related to the data via a reporting distribution:

$$
Y_{adm}(t) \sim \text{NegBinom}\left(X_{adm}(t), \kappa_H\right) \tag{212}
$$

We allow for overdispersion in the observation process to account for noise in the underlying data streams, for example due to day-of-week effects on data collection. We fit the overdispersion parameter $\alpha_H = \frac{1}{\kappa_H}$, which we use for all hospital data streams (including general hospital bed occupancy, ICU bed occupancy, and hospital death data – all of which we describe below) so that they contribute equal weight to the overall likelihood.

The contribution to the likelihood of the data on hospital admissions and new diagnoses in hospital was therefore:

$$
\mathscr{L}_{adm} = \prod_t P_{\text{NegBinom}}\left(Y_{adm}(t)\,|X_{adm}\,(t),\ \kappa_H\,\right) \tag{213}
$$

4.3.3 Hospital bed occupancy by confirmed COVID-19 cases

The model predicted general hospital bed occupancy by confirmed COVID-19 cases, $X_{hosp}(t)$ as:

$$
X_{hosp}(t) := \sum_i \sum_j \sum_k \left(H^{i,j,k}_{R^*}(t) + H^{i,j,k,1}_{D^*}(t) + H^{i,j,k,2}_{D^*}(t) + ICU^{i,j,k}_{pre^*}(t) + W^{i,j,k}_{D^*}(t) + W^{i,j,k,1}_{R^*}(t) + W^{i,j,k,2}_{R^*}(t) \right), \tag{214}
$$

which was related to the observed daily general bed-occupancy via a reporting distribution:

$$
Y_{hosp}\ (t) \sim \text{NegBinom}\left(X_{hosp}(t), \kappa_H\right). \tag{215}
$$

Similarly, the model predicted ICU bed occupancy by confirmed COVID-19 cases, $X_{ICU}(t)$ as:

$$
X_{ICU}(t) := \sum_i \sum_j \sum_k \left(ICU^{i,j,k}_{W_{R^*}}(t) + ICU^{i,j,k}_{W_{D^*}}(t) + ICU^{i,j,k,1}_{D^*}(t) + ICU^{i,j,k,2}_{D^*}(t) \right), \tag{216}
$$

which was related to the observed daily ICU bed-occupancy via a reporting distribution:

$$
Y_{ICU}(t) \sim \text{NegBinom}\left(X_{ICU}(t), \kappa_H\right). \tag{217}
$$

The overall contribution to the likelihood of the data on general bed and ICU bed occupancy was:

$$\begin{aligned}\mathscr{L}_{beds} = &\prod_t P_{\text{NegBinom}}\left(Y_{hosp}(t)\,|X_{hosp}(t),\kappa_H\right)\\ &\times\prod_t P_{\text{NegBinom}}\left(Y_{ICU}(t)\,|X_{ICU}(t),\kappa_H\right).\end{aligned} \tag{218}$$

4.3.4 Hospital, community and care homes COVID-19 deaths

The reported number of daily COVID-19 deaths in hospitals, $Y_{hosp_D}(t)$ was considered as the observed realisation of an underlying hidden Markov process, $X_{hosp_D}(t)$, defined as:

$$X_{hosp_D}(t) := \sum_i\sum_j\sum_k\left(d^{i,j,k}_{H_D,D} + d^{i,j,k}_{H_{D^*},D} + d^{i,j,k}_{ICU_D,D} + d^{i,j,k}_{ICU_{D^*},D} + d^{i,j,k}_{W_D,D} + d^{i,j,k}_{W_{D^*},D}\right), \tag{219}$$

which was related to the data via a reporting distribution:

$$Y_{hosp_D}(t) \sim \text{NegBinom}\left(X_{hosp_D}(t),\kappa_H\right). \tag{220}$$

Similarly, we represented the reported number of daily COVID-19 deaths in the community, $Y_{comm_D}(t)$, as the observed realisations of an underlying hidden Markov process, $X_{comm_D}(t)$, defined as:

$$X_{comm_D}(t) := \sum_{i\neq CHR}\sum_j\sum_k d^{i,j,k}_{G_D,D}, \tag{221}$$

which was related to the data via a reporting distribution:

$$Y_{comm_D}(t) \sim \text{NegBinom}\left(X_{comm_D}(t),\kappa_D\right). \tag{222}$$

Finally, we represented the reported number of daily COVID-19 deaths in care homes, $Y_{CH_D}(t)$, as the observed realisations of an underlying hidden Markov process, $X_{CH_D}(t)$, defined as:

$$X_{CH_D}(t) := d^{CHR}_{G_D,D}, \tag{223}$$

which was related to the data via a reporting distribution:

$$Y_{CH_D}(t) \sim \text{NegBinom}\left(X_{CH_D}(t),\kappa_D\right). \tag{224}$$

We fit the overdispersion parameter $\alpha_D = \frac{1}{\kappa_D}$, for the community and care home death data streams (note that we use the same overdispersion for hospital deaths as we do for other hospital data streams).

The overall contribution to the likelihood of the data on COVID-19 deaths in hospital, in the community and in care homes was:

$$\begin{aligned}\mathscr{L}_{deaths} = &\prod_t P_{\text{NegBinom}}\left(Y_{hosp_D}(t)\,|X_{hosp_D}(t),\kappa_H\right)\\ &\times\prod_t P_{\text{NegBinom}}\left(Y_{comm_D}(t)\,|X_{comm_D}(t),\kappa_D\right)\\ &\times\prod_t P_{\text{NegBinom}}\left(Y_{CH_D}(t)\,|X_{CH_D}(t),\kappa_D\right).\end{aligned} \tag{225}$$

4.3.5 Serosurveys

We model serological testing of all individuals aged 15-64 inclusive, and define the resulting number of seropositive and seronegative individuals (were all individuals aged 15-65 to be tested) from serology flow j (where $j = 1$ corresponds to EuroImmun and $j = 2$ to Roche N), as:

$$X_{sero^j_{pos}}(t) := \sum_{i=[15,20)}^{[60,65)} T^i_{sero^j_{pos}}(t) \tag{226}$$

$$X_{sero^j_{neg}}(t) := \left(\sum_{i=[15,20)}^{[60,65)} N^i \right) - X_{sero^j_{pos}}(t). \tag{227}$$

We compared the observed number of seropositive results, $Y_{sero^j_{pos}}(t)$, with that predicted by our model, allowing for i) the sample size of each serological survey, $Y_{sero^j_{test}}(t)$ and ii) imperfect sensitivity ($p_{sero_{sens}}$) and specificity ($p_{sero_{spec}}$) of the serological assay:

$$Y_{sero^j_{pos}}(t) \sim \text{Binom}\left(Y_{sero^j_{test}}(t), \omega_{sero_{pos}}(t)\right) \tag{228}$$

where:

$$\omega_{sero^j_{pos}}(t) := \frac{p_{sero_{sens}} X_{sero^j_{pos}}(t) + \left(1 - p_{sero_{spec}}\right) X_{sero^j_{neg}}(t)}{X_{sero^j_{pos}}(t) + X_{sero^j_{neg}}(t)}. \tag{229}$$

The contribution to the likelihood of the serosurvey data was:

$$\mathscr{L}_{sero} = \prod_t \prod_{j=1,2} P_{\text{Binom}}\left(Y_{sero^j_{pos}}(t) \middle| X_{sero^j_{test}}(t), \omega_{sero^j_{pos}}(t)\right) \tag{230}$$

4.3.6 PCR testing

As described in the data section (section 4), we fitted the model to PCR testing data from two separate sources:

- Pillar 2 testing: the government testing programme, which recommends that individuals with COVID-19 symptoms are tested [6].
- the REACT-1 study, which aims to quantify the prevalence of SARS-CoV-2 in a random sample of the England population on an ongoing basis [7].

We only use Pillar 2 PCR test results for individuals aged 25 and over (we assume this includes all care home workers and residents). We assume that individuals who get tested through Pillar 2 PCR testing are either newly symptomatic SARS-CoV-2 cases (who will test positive):

$$X_{P2_{pos}}(t) := \sum_{i=[25,30)}^{CHW} \sum_j \sum_k d^{i,j,k}_{I_P,I_{C_1}} \tag{231}$$

or non-SARS-CoV-2 cases who have symptoms consistent with COVID-19 (who will test negative):

$$X_{P2_{neg}}(t) := g(t) \left(\left(\sum_{i=[25,30)}^{CHW} N^i \right) - X_{P2_{pos}}(t) \right), \tag{232}$$

where

$$g(t)=\begin{cases} p_{NC} & \text{if } t \text{ is a weekday} \\ p_{NC}^{weekend} & \text{if } t \text{ is a weekend day} \end{cases} \tag{233}$$

is the probability of non SARS-CoV-2 cases having symptoms consistent with COVID-19 that might lead them to get a PCR test.

We compared the observed number of positive PCR tests, $Y_{P2_{pos}}(t)$ with that predicted by our model, accounting for the number of PCR tests conducted each day under pillar 2, $Y_{P2_{test}}(t)$, by calculating the probability of a positive PCR result (assuming perfect sensitivity and specificity of the PCR test):

$$\omega_{P2_{pos}}(t) := \frac{X_{P2_{pos}}(t)}{X_{P2_{pos}}(t)+X_{P2_{neg}}(t)} \tag{234}$$

People may seek PCR tests for many reasons and thus the pillar 2 data are subject to competing biases. We therefore allowed for an over-dispersion parameter $\rho_{P2_{test}}$, which we fitted separately for each region in the modelling framework:

$$Y_{P2_{pos}}(t) \sim \text{BetaBinom}\left(Y_{P2_{test}}(t), \omega_{P2_{pos}}(t), \rho_{P2_{test}}\right). \tag{235}$$

We incorporated the REACT-1 PCR testing data into the likelihood analogously to the serology data, by considering the model-predicted number of PCR-positives, $X_{R1_{pos}}(t)$, and PCR-negatives, $X_{R1_{neg}}(t)$, were all individuals aged over five and not resident in a care home to be tested:

$$X_{R1_{pos}}(t) := \sum_{i\neq[0,4),CHR} T^{i}_{PCR_{pos}}(t), \tag{236}$$

$$X_{R1_{neg}}(t) := \left(\sum_{i\neq[0,4),CHR} N^{i}\right) - X_{R1_{pos}}(t). \tag{237}$$

We compared the daily number of positive results observed in REACT-1, $Y_{R1_{pos}}(t)$, given the number of people tested on that day, $Y_{R1_{test}}(t)$, to our model predictions, by calculating the probability of a positive result, assuming perfect sensitivity and specificity of the REACT-1 assay:

$$\omega_{R1_{pos}}(t) := \frac{X_{R1_{pos}}(t)}{X_{R1_{pos}}(t)+X_{R1_{neg}}(t)} \tag{238}$$

so

$$Y_{R1_{pos}}(t) \sim \text{Binom}\left(Y_{R1_{test}}(t), \omega_{R1_{pos}}(t)\right). \tag{239}$$

The contribution to the likelihood of the PCR testing data was:

$$\begin{aligned} \mathscr{L}_{PCR} = \prod_t P_{\text{BetaBinom}}\left(Y_{P2_{pos}}(t)\,\middle|\,Y_{P2_{test}}(t), \omega_{P2_{pos}}(t), \rho_{P2_{test}}\right) \\ \times \prod_t P_{\text{Binom}}\left(Y_{R1_{pos}}(t)\,\middle|\,Y_{R1_{test}}(t), \omega_{R1_{pos}}(t)\right) \end{aligned} \tag{240}$$

4.3.7 Variant and Mutation data

To inform the replacement of the Alpha variant by the Delta variant, we fitted to Variant and Mutation (VAM) data. We assume that samples tested for VAM are newly symptomatic cases (across all groups), with the number for $Alpha$ and for $Delta$ given by

$$X_{VAM_{Alpha}}(t) := \sum_i \sum_k d^{i,Alpha,k}_{I_P,I_{C_1}} \tag{241}$$

$$X_{VAM_{Delta}}(t) := \sum_i \sum_k d_{I_P,I_{C_1}}^{i,Delta,k} + d_{I_P,I_{C_1}}^{i,Alpha+Delta,k} \tag{242}$$

We compared the observed number of Delta VAM tests, $Y_{VAM_{Delta}}(t)$ with that predicted by our model, accounting for the total number of VAM tests conducted each day, $Y_{VAM_{test}}(t)$, by calculating the probability of a Delta VAM test result:

$$\omega_{VAM_{Delta}}(t) := \frac{X_{VAM_{Delta}}(t)}{X_{VAM_{Alpha}}(t) + X_{VAM_{Delta}}(t)} \tag{243}$$

so

$$Y_{VAM_{Delta}}(t) \sim \text{Binom}\left(Y_{VAM_{test}}(t), \omega_{VAM_{Delta}}(t)\right). \tag{244}$$

The contribution to the likelihood of the VAM data was:

$$\mathscr{L}_{VAM} = \prod_t P_{\text{Binom}}\left(Y_{VAM_{Delta}}(t) \,|Y_{VAM_{test}}(t), \omega_{VAM_{Delta}}(t)\right) \tag{245}$$

4.3.8 Full likelihood

The overall likelihood was then calculated as the product of the likelihoods of the individual observations, i.e.:

$$\mathscr{L} = \mathscr{L}_{adm} \times \mathscr{L}_{beds} \times \mathscr{L}_{deaths} \times \mathscr{L}_{sero} \times \mathscr{L}_{PCR} \times \mathscr{L}_{VAM}. \tag{246}$$

4.4 Reproduction number

Both $R^j(t)$ and $R_e^j(t)$ are calculated using next generation matrix (NGM) methods [40], and only consider mixing in the general population, i.e. we do not consider the CHW and CHR age categories.

Note that in this calculation only, we make a simplifying assumption that individuals cannot change vaccine strata between initial infection and the end of their infectious period (or death).

To compute the next generation matrix, we calculated the mean duration of infectiousness weighted by infectivity (asymptomatic individuals are less infectious than symptomatic individuals by factor θ_{I_A}) for an individual in group i and vaccine stage k, $\Delta_I^{i,k}$:

$$\Delta_I^{i,k} = \theta_{I_A}\left(1 - p_C^{i,j,k}\right)\mathbb{E}\left[\tau_{I_A}\right] + p_C^{i,j,k}\left(\mathbb{E}\left[\tau_{I_P}\right] + \mathbb{E}\left[\tau_{I_{C_1}}\right]\right). \tag{247}$$

Note that $\Delta_I^{i,k}$ does not depend on j, as we assume the same duration spent in compartments and probability of being symptomatic between variants. The next generation matrices for the variants were calculated as,

$$NGM_{i,i'}^{Alpha}(t) = m_{i,i'}(t)\Delta_I^{i,0}N^{i'}, \tag{248}$$

$$NGM_{i,i'}^{Delta}(t) = m_{i,i'}(t)\xi^{i,Delta,0}\Delta_I^{i,0}N^{i'}, \tag{249}$$

where ξ is the infectivity of an individual (fully defined in eq. (2)), N^i is the total population of age group i, and with $R^j(t)$ taken as the dominant eigenvalue of the 17 by 17 matrix $NGM^j(t)$. The element $NGM_{i,i'}^j(t)$ is therefore defined as the average number of secondary cases that an individual in age group i' infected with variant j at time t would generate among age group i.

The effective next generation matrices for the variants were calculated as

$$NGM^{Alpha,e}_{D(i,k),D(i',k')}(t) = m_{i,i'}(t)\chi^{i,Alpha,k}\xi^{i,Alpha,k'}\Delta_I^{i,k}S^{i',k'}(t), \tag{250}$$

$$NGM^{Delta,e}_{D(i,k),D(i',k')}(t) = m_{i,i'}(t)\chi^{i,Delta,k}\xi^{i,Delta,k'}\Delta_I^{i,k}\left(S^{i',k'}(t) + (1-\eta)R^{i',Alpha,k'}(t)\right), \tag{251}$$

where $D : \{[0-4],[5-9],\ldots,[75-79],80+\} \times \{0,1,2,3\} \to \{1,2,\ldots,68\}$ is a one-to-one mapping. Then $R_e^j(t)$ is taken as the dominant eigenvalue of the 68 by 68 matrix $NGM^{j,e}(t)$.

We calculate the reproduction numbers weighted by the two variants as

$$R(t) = \frac{w_{Alpha}(t)R^{Alpha}(t) + w_{Delta}(t)R^{Delta}(t)}{w_{Alpha}(t) + w_{Delta}(t)} \tag{252}$$

$$R_e(t) = \frac{w_{Alpha}(t)R_e^{Alpha}(t) + w_{Delta}(t)R_e^{Delta}(t)}{w_{Alpha}(t) + w_{Delta}(t)}, \tag{253}$$

where the weightings $w_j(t)$ are weightings based on the infectious prevalence of each variant (accounting for the baseline relative infectivity of each compartment), such that for $j = Alpha, Delta$,

$$w_j(t) = \sum_i \sum_k \left(\theta_{I_A} I_A^{i,j,k}(t) + I_P^{i,j,k}(t) + I_{C_1}^{i,j,k}(t)\right). \tag{254}$$

4.5 Fixed parameters

We used parameter values calibrated to data from 19 July 2021.

Parameter	Definition	Value	Source
$1/\gamma_U$	Mean time to confirmation of SARS-CoV-2 diagnosis within hospital.	2 days	[41]
$1/\gamma_R$	Mean duration of natural immunity following infection.	3, 6, ∞ years	[42], (sensitivity)
$p_{sero_{pos}}$	Probability of seroconversion following infection.	0.85	[43]
$1/\gamma_{sero_{pre}}$	Mean time to seroconversion from onset of infectiousness.	13 days	[44]
$1/\gamma_{sero^1_{pos}}$	Mean duration of seropositivity (Euroimmun assay).	200 days	[43, 45, 46]
$1/\gamma_{sero^2_{pos}}$	Mean duration of seropositivity (Roche N).	400 days	[43, 45, 46]
$p_{sero_{spec}}$	Specificity of serology test.	0.99	[43]
$p_{sero_{sens}}$	Sensitivity of serology test.	1	Assumed
η	Proportion of cross-immunity to $Delta$ following infection with $Alpha$.	0.75,0.85,1	[47], (sensitivity)
θ_{I_A}	Infectivity of an asymptomatic individual, relative to a symptomatic individual.	0.223	[1]

Table S8: Fixed model parameter notations, values, and evidence-base.

Among recently infected individuals who remain antibody-negative by serological tests, it is not possible to distinguish how many do not mount an antibody response sufficient to be detected versus how many mount an antibody response that a specific test does not detect (what we refer to as the test's lack of sensitivity). From the inference point of view, we are interested in the proportion of recently infected individuals who develop a detectable antibody response i.e. the product of i) the probability of (detectable) seroconversion following infection; and ii) the sensitivity of the serology test. Here we have set the probability of seroconversion (or proportion of individuals who develop an antibody response) to 0.85 as per [43] and the test sensitivity to 1. We could have equally chosen any pair of values in $[0,1]^2$ whose product equals 0.85. In practice this assumption does not affect the inference framework.

We also assume that the performance of the tests (PCR and serological assays) are the same for all variants.

4.6 Prior distributions

Prior distributions are described in table S9. Informative prior distributions for the single strain model are the same as prior distributions in the model given in [1]. In the absence of evidence from the literature (or because existing evidence has been derived from the same datasets we use in our study), uninformative or weakly informative prior distributions have been chosen for the two-strain model; the prior for t_{Delta} covers a wide period of time spanning over more than two months and the assumption of σ, and the prior for the Delta transmission advantage is assumed uniform between [0,3]."

Table S9: Inferred model parameter notations and prior distributions. Region codes: NW = North West, NEY = North East and Yorkshire, MID = Midlands, EE = East of England, LON = London, SW = South West, SE = South East

	Description	Region	Prior distribution
t_0	Start date of regional outbreak (2020-mm-dd)	All	$U[01-01, 03-15]$
t_{Delta}	Delta seeding date (2021-mm-dd)	All	$U[03-10, 05-31]$
σ	Delta transmission advantage	All	$U(0,3)$
$\beta(t)$	Transmission rate (pp) at $t = dd/mm/yy$		
β_1	16/03/20: PM advises WFH and essential travel only	All	$\Gamma(136, 0.0008)$
β_2	23/03/20: PM announces lockdown 1	All	$\Gamma(3.73, 0.0154)$
β_3	25/03/20: Lockdown 1 into full effect	All	$\Gamma(4.25, 0.0120)$
β_4	11/05/20: Initial easing of lockdown 1	All	$\Gamma(4.25, 0.0120)$
β_5	15/06/20: Non-essential shops re-open	All	$\Gamma(4.25, 0.0120)$
β_6	04/07/20: Hospitality re-opens	All	$\Gamma(4.25, 0.0120)$
β_7	01/08/20: "Eat out to help out" scheme starts	All	$\Gamma(4.25, 0.0120)$
β_8	01/09/20: Schools and universities re-open	All	$\Gamma(4.25, 0.0120)$
β_9	14/09/20: "Rule of six" introduced	All	$\Gamma(4.25, 0.0120)$
β_{10}	14/10/20: Tiered system introduced	All	$\Gamma(4.25, 0.0120)$
β_{11}	31/10/20: Lockdown 2 announced	All	$\Gamma(4.25, 0.0120)$
β_{12}	05/11/20: Lockdown 2 starts	All	$\Gamma(4.25, 0.0120)$
β_{13}	02/12/20: Lockdown 2 ends	All	$\Gamma(4.25, 0.0120)$
β_{14}	18/12/20: School holidays start	All	$\Gamma(4.25, 0.0120)$
β_{15}	25/12/20: Last day of holiday season relaxation	All	$\Gamma(4.25, 0.0120)$
β_{16}	05/01/21: Lockdown 3 starts	All	$\Gamma(4.25, 0.0120)$
β_{17}	08/03/21: Roadmap step one - schools reopen	All	$\Gamma(4.25, 0.0120)$
β_{18}	01/04/21: School holidays	All	$\Gamma(4.25, 0.0120)$
β_{19}	19/04/21: Roadmap step two - outdoor rule of 6 (12/04) and schools re-open (19/04)	All	$\Gamma(2.72, 0.0292)$
β_{20}	17/05/21: Roadmap step three - Indoor hospitality opens	All	$\Gamma(2.72, 0.0292)$
β_{21}	21/06/21: Wedding and care home restrictions eased	All	$\Gamma(2.72, 0.0292)$
β_{22}	03/07/21: Euro 2020 quarter finals (cited as significant influence [48])	All	$\Gamma(2.72, 0.0292)$
β_{23}	11/07/21: End of Euros football tournament	All	$\Gamma(2.72, 0.0292)$
β_{24}	19/07/21: Full lift of NPIs	All	$\Gamma(2.72, 0.0292)$

Continued on next page

**Table S9 – continued from previous page**			
	Description	Region	Prior distribution
ε	Relative reduction in contacts between CHR and the general population	All	$B(1,1)$
m_{CHW}	Transmission rate between CHR and CHW	NW	$\Gamma(5, 4.3 \times 10^{-7})$
		NEY	$\Gamma(5, 3.7 \times 10^{-7})$
		MID	$\Gamma(5, 2.9 \times 10^{-7})$
		EE	$\Gamma(5, 5.2 \times 10^{-7})$
		LON	$\Gamma(5, 7.6 \times 10^{-7})$
		SW	$\Gamma(5, 4.9 \times 10^{-7})$
		SE	$\Gamma(5, 3.1 \times 10^{-7})$
m_{CHR}	Transmission rate among CHR	NW	$\Gamma(5, 4.3 \times 10^{-7})$
		NEY	$\Gamma(5, 3.7 \times 10^{-7})$
		MID	$\Gamma(5, 2.9 \times 10^{-7})$
		EE	$\Gamma(5, 5.2 \times 10^{-7})$
		LON	$\Gamma(5, 7.6 \times 10^{-7})$
		SW	$\Gamma(5, 4.9 \times 10^{-7})$
		SE	$\Gamma(5, 3.1 \times 10^{-7})$
$p_{H,1}^{max}, p_{H,2}^{max}, p_{H,3}^{max}$	The probability of symptomatic individuals developing serious disease requiring hospitalisation, for the group with the largest probability at different timepoints (see Section 4.2.2)	All	$B(15.8, 5.28)$
$p_{G_D}^{max}$	Probability of death in the community given disease severe enough for hospitalisation	All	$B(1,1)$
$p_{G_D}^{CHR}$	Probability of death in CHR given disease severe enough for hospitalisation	All	$B(1,1)$
$p_{ICU,1}^{max}, p_{ICU,2}^{max}$	Probability of triage to ICU for new hospital admissions, for the group with the largest probability at different timepoints (see Section 4.2.2)	All	$B(13.9, 43.9)$
$p_{H_D}^{max}$	Initial probability of death for general inpatients	All	$B(42.1, 50.1)$
$p_{ICU_D}^{max}$	Initial probability of death for ICU inpatients	All	$B(60.2, 29.3)$
$p_{W_D}^{max}$	Initial probability of death for stepdown inpatients	All	$B(28.7, 52.1)$
$\mu_{D,1}, \mu_{D,2}$	Hospital mortality multipliers due to changes in clinical care at different timepoints (see Section 4.2.2)	All	$B(1,1)$
$\mu_{\gamma_H,1}, \mu_{\gamma_H,2}, \mu_{\gamma_H,3}$	Mean duration multipliers for non-ICU hospital compartments at different timepoints (see Section 4.2.2)	All	$B(1,1)$
p_{NC}	Prevalence of non-COVID symptomatic illness that could lead to getting a PCR test	All	$B(1,1)$
$p_{NC}^{weekend}$	Prevalence of non-COVID symptomatic illness that could lead to getting a PCR test on a weekend	All	$B(1,1)$
$\rho_{P2_{test}}$	Overdispersion of PCR positivity	All	$B(1,1)$
α_H	Overdispersion for hospital data streams	All	$B(1,1)$
α_D	Overdispersion for death data streams	All	$B(1,1)$

4.7 Running the model

The model is run three times to cover central, optimistic, and pessimistic scenarios. These are reflected in the fixed parameters (Table S9) and VE (Table S3) tables. Specifically these scenarios are given by setting all fixed parameters above with:

Assumptions	Cross-immunity, η	Mean duration natural immunity, $1/\gamma_R$	Vaccine effectiveness vs Delta
Optimistic	1	∞	optimistic (Table S3)
Central	0.85	6 years	central (Table S3)
Pessimistic	0.75	3 years	pessimistic (Table S3)

Table S10: Combinations of parameters that are run in the model in three distinct scenarios. See Table S9 for details of the parameters and Table S3 for the vaccine effectiveness scenarios.

5 Forward Projections

The previous sections provided methodological details of our model structure and how we fit to existing epidemiological data. This section outlines information and assumptions made for simulating forward projections from the fitted model. Simulated scenarios depend primarily on assumptions regarding future transmissibility and vaccine rollout, which we discuss in sections 5.1 and 5.2 respectively. The range of simulated scenarios that we run, including counterfactual runs, are detailed in Section 5.3.

The simulated model is run three times, corresponding to the three model fits from the central/optimistic/pessimistic assumptions (Section 4.7). These assumptions are carried forward into the simulated model, e.g. optimistic assumptions are made in the simulation model when using the optimistic model fits (same for central and pessimistic). As sensitivity analyses we vary these assumptions in the simulations for each of the three model fits (Section 5.4).

5.1 Transmissibility of SARS-CoV-2

There is considerable uncertainty on the level of transmissibility of SARS-CoV-2 following the final stage of non-pharmaceutical intervention (NPI) lifting, 'step four'. The overall reproduction number will depend on the relative frequency of each SARS-CoV-2 variant in circulation, the ability of the virus to be transmitted upon contact between two individuals (which may depend on environmental factors such as temperature therefore displaying seasonal effects), the level of physical mixing between individuals (which could be low if individuals do not immediately return to their pre-pandemic behaviours, or could be high if individuals suddenly compensate for months of low mixing), and the level of susceptibility in the population.

5.1.1 NPI lifting: the new baseline

The final level of transmissibility once all NPIs are lifted is determined by the assumptions made in the given simulation scenario. To estimate the value of $R^{Alpha}(t)$ in Step 4 we took the average $R^{Alpha}(t)$ over Step 3 in June and July then multiplied this value by 1.4, 1.7, or 2 to correspond with an optimistic, central, or pessimistic contact mixing assumption. Table S11 summarises the estimated average $R^{Alpha}(t)$ values in Step 3 and assumed values in Step 4 for each of the three contact mixing assumptions, as well as for the three model assumptions (Section 4.7). By averaging over all of June and July, the same values for Step 4 can be utilised in all scenarios (independent of the full lift date).

The estimated level of transmissibility depends on the assumptions around VE, immunity and cross protection. In the optimistic scenario (Table S11 row 1), where VE and cross-protection are high and immunity is long-lasting, a high level of transmission is required to explain the level of observed cases. Conversely, in the pessimistic scenario (Table S11 row 3), a lower level of transmission can explain the observed number of cases, due to low VE and cross-protection, as well as immunity waning after 3 years.

Model assumption	Average $R_{S3}^{Alpha}(t)$	Assumed $R_{S4}^{Alpha}(t)$	Average $R_{S3}^{Delta}(t)$	Assumed $R_{S4}^{Delta}(t)$
Optimistic	1.74	{2.44, 2.96, 3.49}	3.43	{4.81, 5.84, 6.87}
Central	1.59	{2.23, 2.70, 3.18}	2.82	{3.95, 4.80, 5.65}
Pessimistic	1.45	{2.02, 2.46, 2.89}	2.41	{3.37, 4.09, 4.81}

Table S11: Forward projection scenarios with $R^j(t)$ values based on: model assumptions (column 1) (Section 4.7), average $R^{Alpha}(t)$ in Step 3 between 2021-06-01 and 2021-07-31 (column 2) and same for $R^{Delta}(t)$ (column 4), $R^{Alpha}(t)$ in Step 4 (column 3) and $R^{Delta}(t)$ in Step 4 (column 5) under different contact mixing assumptions (1.4x, 1.7x, 2x Step 3).

$R^j(t)$ is assumed to vary through time in our forward projections, in accordance with school term dates and seasonal variation. To capture this variation we assumed $R^j(t)$ under each level of restrictions was distributed lognormally with means as given in Table S11, and assume a standard deviation of 0.2. For each NPI lifting scenario, we sampled from the relevant distributions of $R^j(t)$ and generated sampled trajectories of $R^j(t)$ over time by matching the ranked values obtained for each step, where distinct steps are defined by the opening and closing of schools as defined in Table S12 below. This constraint was added to ensure that $R^j(t)$ could only increase over time except for decreases due to school closures and seasonality.

5.1.2 School closures

School closures can break the chains of transmission between households and reduces contacts between children, and children and adults. This can therefore reduce the level of transmission of respiratory pathogens such as SARS-CoV-2. We accounted for this by assuming that during school holidays $R^j(t)$ decreases by 0.25 compared to during term-time. We applied this multiplier (and seasonality assumptions detailed below in Section 5.1.3) at each associated term start/end date (Table S12). The magnitude of the difference in $R^j(t)$ is based on the consensus value from SPI-M accounting for the increase in transmission due to the emergence of the Alpha variant, and is consistent with the impact of school closures estimated during step two from our model fits [49]. School closure dates were taken from the average term dates agreed by councils in England, and presented in Table S12.

Start/End Dates	Definition	Open/Closed
2021-07-24 - 2021-08-31	Summer holidays	Closed
2021-09-01 - 2021-10-22	Autumn term	Open
2021-10-23 - 2021-10-31	Half term	Closed
2021-11-01 - 2021-12-17	Autumn term	Open
2021-12-18 - 2022-01-03	Winter holidays	Closed
2022-01-04 - 2022-02-11	Spring term	Open
2022-02-12 - 2022-02-20	Half term	Closed
2022-02-21 - 2022-04-01	Spring term	Open
2022-04-02 - 2022-04-18	Easter holidays	Closed
2022-04-19 - 2022-05-27	Summer term	Open
2022-05-28 - 2022-06-05	Half term	Closed
2022-06-06 - 2022-07-22	Summer term	Open

Table S12: School term dates for 2021/2022 academic year, based on the average across England.

5.1.3 Seasonality

In our main analyses we assumed a slight seasonal trend in SARS-CoV-2 transmissibility throughout the year in England with 20% relative peak to trough variation. Clear evidence has yet to be demonstrated for seasonality of SARS-CoV-2 transmission, however our 20% variation assumption is considered reasonable based on theoretical modelling approaches and comparisons to pre-existing coronaviruses [50, 51]. We computed a daily multiplier for transmissibility which was:

- Maximal at 1.1 in mid-February of each year (10% relative increase compared to the mean transmissibility)
- Minimal at 0.9 on in mid-August (day 228) of each year (10% relative decrease compared to the mean transmissibility)

We applied this seasonal multiplier (Figure S8) to the value of $R^j(t)$ at each term start/end date.

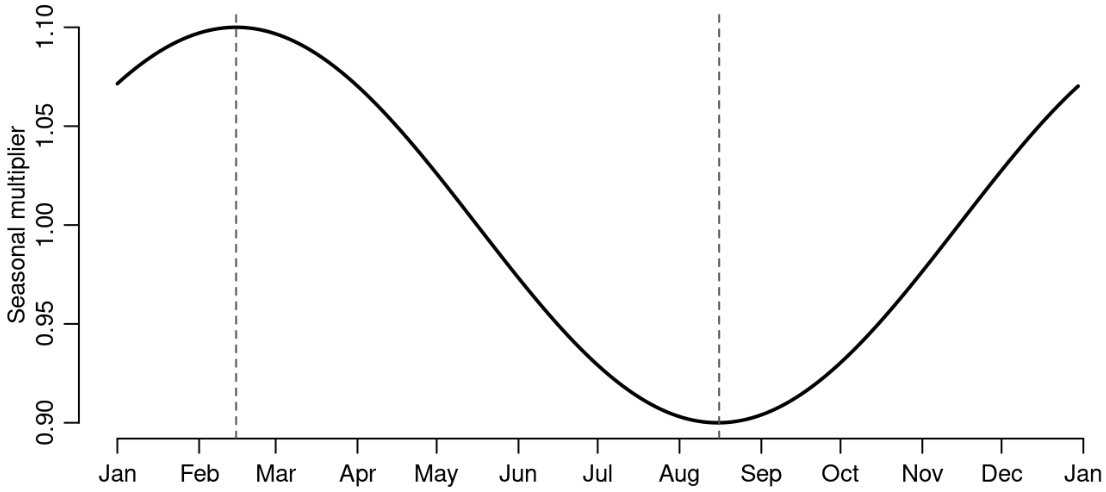

Figure S8: Seasonal daily multiplier for transmissibility ($R^j(t)$) applied at future change steps.

As specified above, we assumed $R^j(t)$ under each level of restrictions was distributed lognormally. For each NPI lifting scenario, we sampled from the relevant distributions of $R^j(t)$ and generated sampled trajectories of $R^j(t)$ over time by matching the ranked values obtained for each step.

5.2 Vaccine roll-out

Projections of future vaccine roll-out are assumed to be 1.9 million doses per week on average [52]. For individuals aged over 50 years, we assumed that PF and AZ vaccines continue to be distributed in the proportions observed up to 31 July 2021 in each age group (Figure S4). We assumed a 60% AZ and 40% PF/Mod mix for 40-49 year olds, and assumed all individuals under 40 would receive PF or Mod. We further assume a maximum uptake for each group as summarised in Table S13. All other vaccine assumptions are the same as detailed as in Section 3.

5.3 Counterfactual simulations

As well as simulating the roadmap on the planned dates, we also simulated four counterfactual projections (Table S14):

1. Truth: Step four of the UK roadmap happened on 19 July 2021 and Delta was present. We assumed that social mixing increases gradually so that $R^j(t)$ linearly increases to the assumed step 4 $R^j(t)$ values (Table S11) over 11 weeks (Table S15).

Group	Reported NHS first dose uptake data up to 19 July 2021^	Maximum uptake in the simulation
Care home residents (CHR)	-	95%
Care home workers (CHW)	-	86%
80+ years*	97.6%	95%
75-79 years*	100%+	99%
70-74 years*	98%	99%
65-69 years*	95.4%	97%
60-64 years*	99.9%	99%
55-59 years*	97.7%	98%
50-54 years*	92.1%	95%
45-49 years*	86.1%	90%
40-44 years*	89%	90%
35-39 years*	80.7%	80%
30-34 years*	75.9%	80%
25-29 years*	66.2%	80%
18-24 years*	62%	80%

* not care home resident/worker.
^ NIMS data, as announced 16 July 2021 [53].
+ Signifies first doses exceeds the latest estimate of the population from ONS for this group.

Table S13: Onward vaccine uptake assumptions by group or age.

Name	Step four date	VOC	$R^{Alpha}(t)$
JulyGradual	19 July 2021	Alpha and Delta	Gradual over 11 weeks to Table S11*
Jul-19	19 July 2021	Alpha and Delta	Table S11
Jun-21	21 June 2021	Alpha and Delta	Table S11 rows 4-6
JuneGradual	21 June 2021	Alpha and Delta	Gradual over 11 weeks to Table S11*
AlphaOnly	21 June 2021	Alpha Only	Table S11

Table S14: Projected scenarios including the name of the scenario used in plots in the manuscript, the assumed date of step four, whether we seed a variant of concern (Delta), and the assumed $R^j(t)$ for Alpha. *For the 'Gradual' scenarios, $R^{Alpha}(t)$ gradually increases according to the schedules in Tables S15 and S16.

2. Counterfactual (CF): Step four of the UK roadmap happened on 19 July 2021 and Delta was present. We assumed a rapid increase in $R^j(t)$ to the values stated in Table S11.

3. CF: Step four happened on 21 June 2021 and Delta was present. We assumed a rapid increase in $R^j(t)$ to the values stated in Table S11.

4. CF: Step four happened on 21 June 2021 and Delta was present. We assumed that social mixing increases gradually so that $R^j(t)$ linearly increases to the assumed step 4 $R^j(t)$ values (Table S11) over 11 weeks (Table S16).

5. CF: Step four happened on 21 June 2021 but with no seeding of Delta. In this case we do not switch to a two-variant model but instead simulate from 8 March 2021 without seeding Delta. On the date of

step four we assume the same values as in Table S11. For simulated days between 8 March 2021 and 20 June 2021 we use the $R^{Alpha}(t)$ values estimated in the two-variant model.

Date	$R^{Alpha}(t)$	$R^{Delta}(t)$
End Step 3	1.17	2.06
2021-07-19	{1.27, 1.31, 1.36}	{2.23, 2.31, 2.38}
2021-07-26	{1.37, 1.45, 1.54}	{2.40, 2.56, 2.71}
2021-08-03	{1.46, 1.59, 1.72}	{2.57, 2.80, 3.04}
2021-08-10	{1.56, 1.73, 1.90}	{2.75, 3.05, 3.36}
2021-08-18	{1.65, 1.87, 2.09}	{2.92, 3.30, 3.69}
2021-08-25	{1.75, 2.01, 2.27}	{3.09, 3.55, 4.02}
2021-09-01	{1.84, 2.15, 2.45}	{3.26, 3.80, 4.34}
2021-09-09	{1.94, 2.29, 2.63}	{3.44, 4.05, 4.67}
2021-09-16	{2.04, 2.43, 2.82}	{3.61, 4.30, 5.00}
2021-09-24	{2.13, 2.56, 3.00}	{3.78, 4.55, 5.32}
2021-10-01	{2.23, 2.70, 3.18}	{3.95, 4.80, 5.65}

Table S15: Gradual July scenario $R^{Alpha}(t)$ schedule under central assumptions. The value of $R^{Alpha}(t)$ estimated at 2021-07-18 linearly increases over 11 weeks to the assumed Step 4 $R^{Alpha}(t)$ value (Table S11). The three $R^{Alpha}(t)$ values correspond to optimistic/central/pessimistic mixing assumptions.

Date	$R^{Alpha}(t)$	$R^{Delta}(t)$
End Step 3	1.56	2.81
2021-06-21	{1.62, 1.66, 1.71}	{2.91, 2.99, 3.07}
2021-06-28	{1.68, 1.77, 1.85}	{3.02, 3.17, 3.33}
2021-07-05	{1.74, 1.87, 2.00}	{3.12, 3.35, 3.58}
2021-07-13	{1.80, 1.98, 2.15}	{3.22, 3.53, 3.84}
2021-07-20	{1.86, 2.08, 2.30}	{3.33, 3.71, 4.10}
2021-07-27	{1.92, 2.18, 2.44}	{3.43, 3.90, 4.36}
2021-08-03	{1.98, 2.29, 2.59}	{3.54, 4.08, 4.62}
2021-08-10	{2.04, 2.39, 2.74}	{3.64, 4.26, 4.87}
2021-08-18	{2.11, 2.50, 2.89}	{3.75, 4.44, 5.13}
2021-08-25	{2.17, 2.60, 3.03}	{3.85, 4.62, 5.39}
2021-09-01	{2.23, 2.70, 3.18}	{3.95, 4.80, 5.65}

Table S16: Gradual June scenario $R^{Alpha}(t)$ schedule under central assumptions. The value of $R^{Alpha}(t)$ estimated at 2021-06-20 linearly increases over 11 weeks to the assumed Step 4 $R^{Alpha}(t)$ value (Table S11). The three $R^{Alpha}(t)$ values correspond to optimistic/central/pessimistic mixing assumptions.

5.4 Sensitivity analysis

Finally as a sensitivity analysis we project forward the two July scenarios (Table S14, rows 1-2) under different combinations of:

1. Vaccine effectiveness vs Delta - Table S3.
2. Cross immunity vs Delta - {1, 0.85, 0.75} (Table S8).

3. Waning immunity - {None, 6 years, 3 years} (Table S8).
4. Contact mixing - {Optimistic, central, pessimistic} (Table S11).

6 Software and Implementation

Implementation of the model described above is fully described in FitzJohn et al. [54]. The primary interface to the model is coded in R [55] with functions written in packages `sircovid` and `spimalot`. The model is written in `odin` and run with `dust`, the pMCMC functions are written in `mcstate`. For this paper we used `sircovid` v0.11.30, `spimalot` v0.2.52, `dust` v0.9.11, and `mcstate` v0.6.5. The above packages are publicly available in the *mrc-ide* GitHub organisation (https://github.com/mrc-ide/). The code and scripts used to create the results in this paper are available in https://github.com/mrc-ide/sarscov2-roadmap-england.

7 Supplementary Results

7.1 Model fit to data

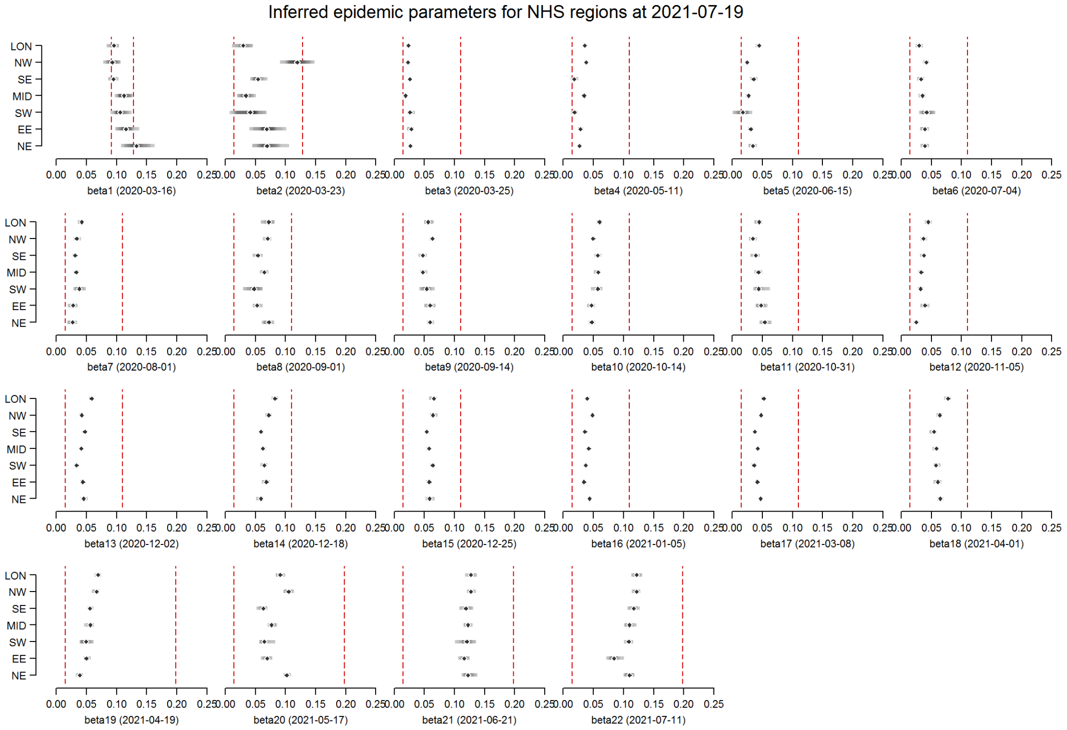

Figure S9: Posterior distributions for β_i for initial variant-agnostic model fits. Red bars indicate prior distribution range, black bars indicate the 95% CrI. As explained in Section 2.1, the parameters corresponding to after the two-variant model is introduced (β_{17} - β_{22}) are later re-calculated after model restarts, using the posterior distributions presented here as prior distributions. Final posterior distributions for β_{17} - β_{24} are presented below in Figure S10.

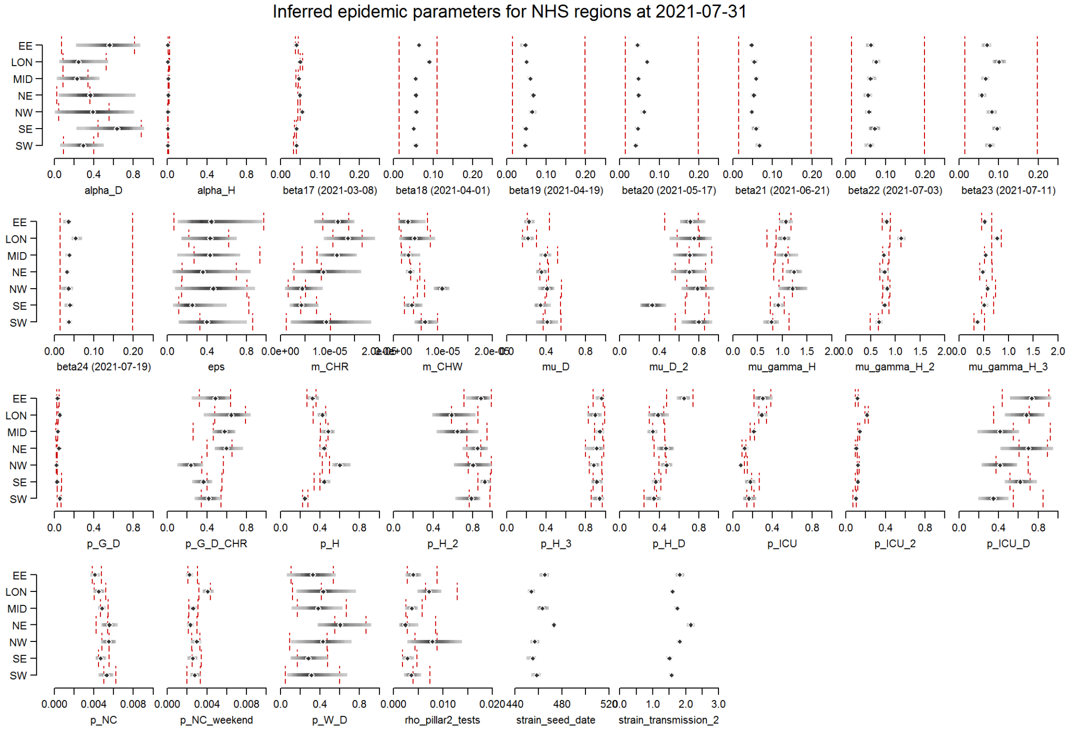

Figure S10: Final model parameter posterior distributions. Red bars indicate prior distribution range, black bars indicate the 95% CrI.

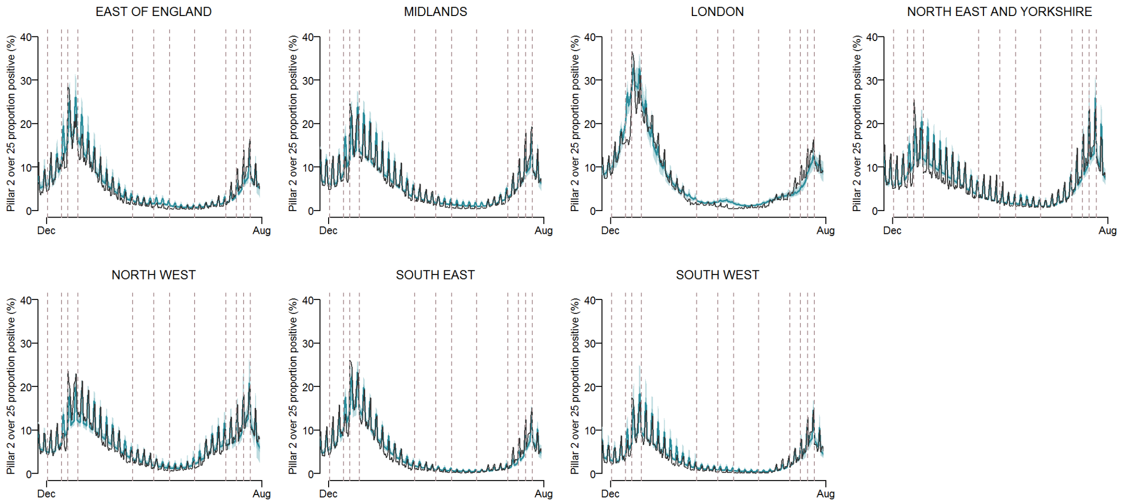

Figure S11: Model fits by region to PCR positivity from Pillar 2 for individuals aged > 25 years. The solid blue line shows the median model fit and the shaded area the 95% CrI. The black line depicts the recorded data.

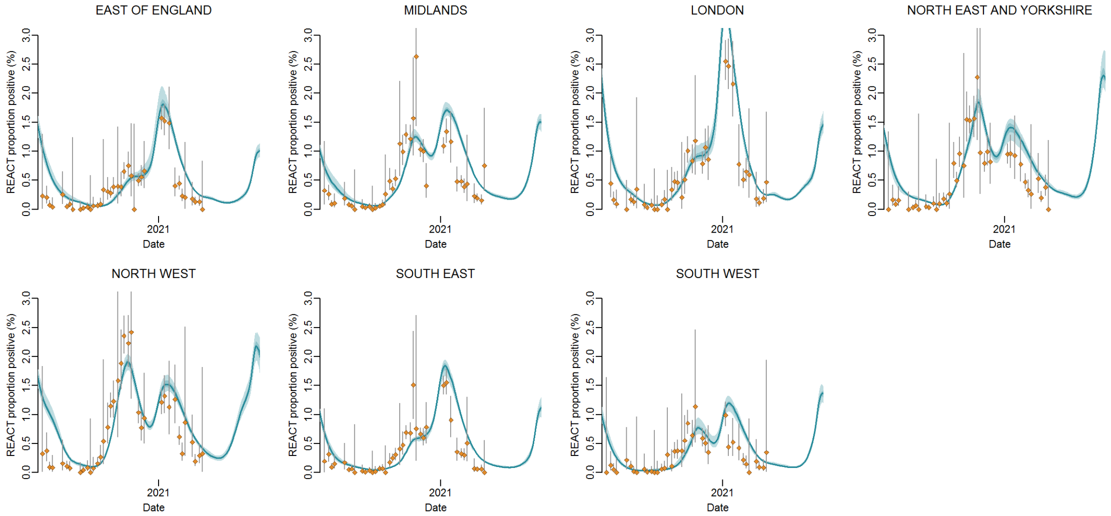

Figure S12: Model fits by region to PCR positivity from the REACT-1 study . The points show the data and bars the 95% CI. The solid blue line shows the median model fit and the shaded area the 95% CrI.

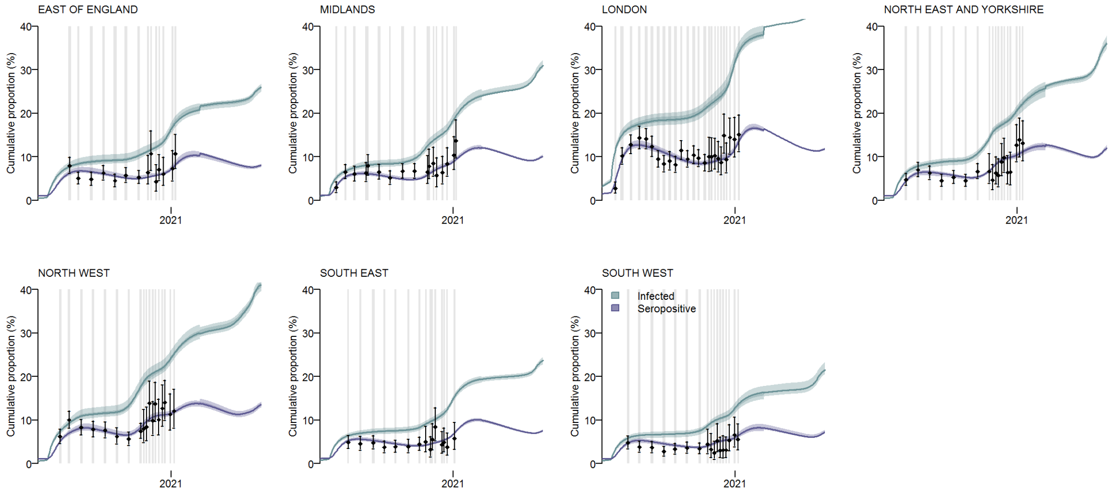

Figure S13: Model fits by region to EuroImmun serology assay data (Table S1). The points show the data and bars the 95% CI. The solid purple line shows the median model fit, the solid blue line shows the median cumulative infections, the shaded areas the respective 95% CrI.

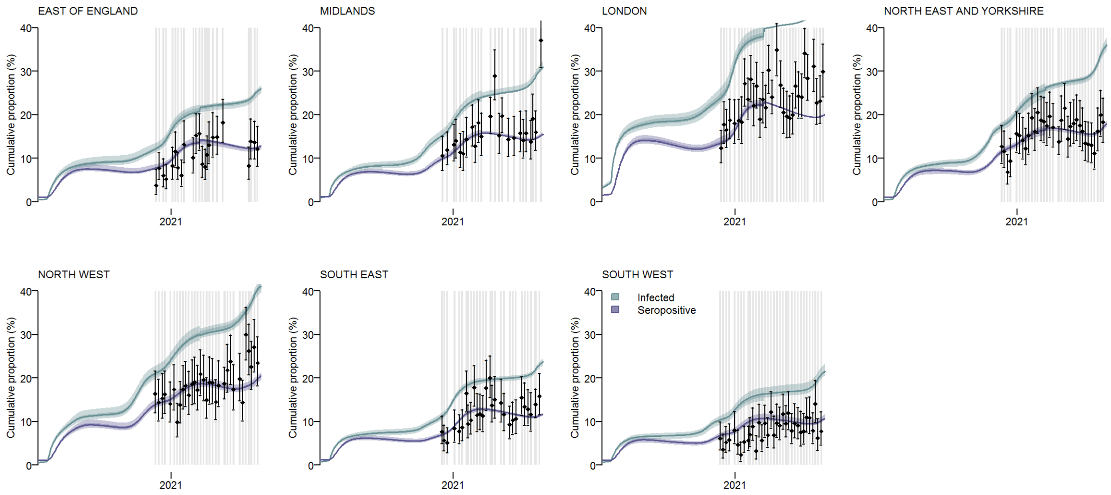

Figure S14: Model fits by region to Roche serology assay data (Table S1). The points show the data and bars the 95% CI. The solid purple line shows the median model fit, the solid blue line shows the median cumulative infections, the shaded areas the respective 95% CrI.

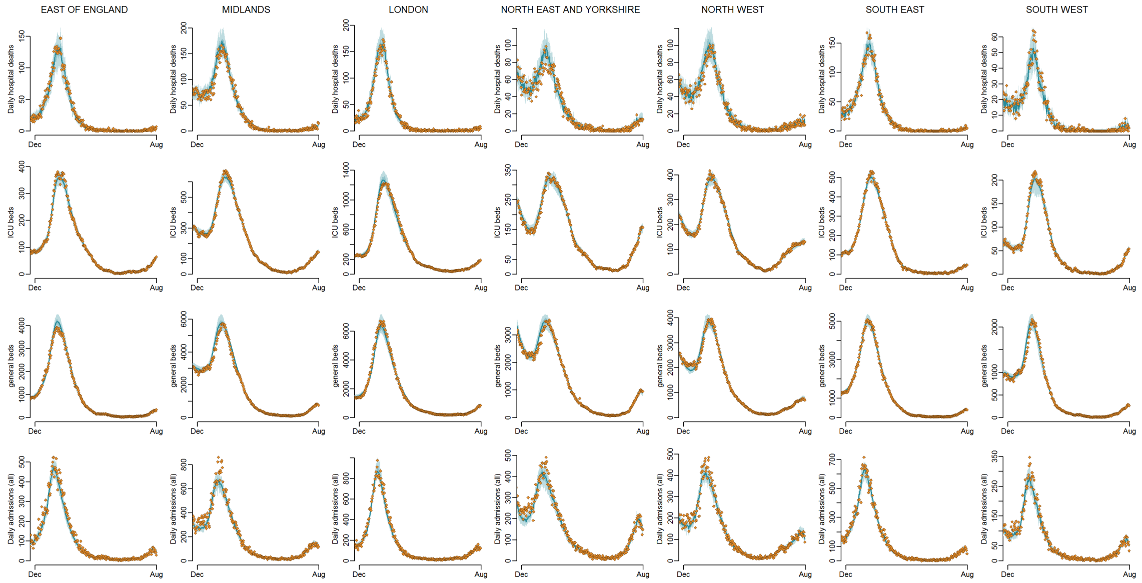

Figure S15: Model fits to England NHS regions: daily hospital deaths (top row), all hospital beds occupancy (second row), and all daily admissions (bottom row) by England NHS region (columns). The points show the data, the solid line the median model fit and the shaded area the 95% CrI.

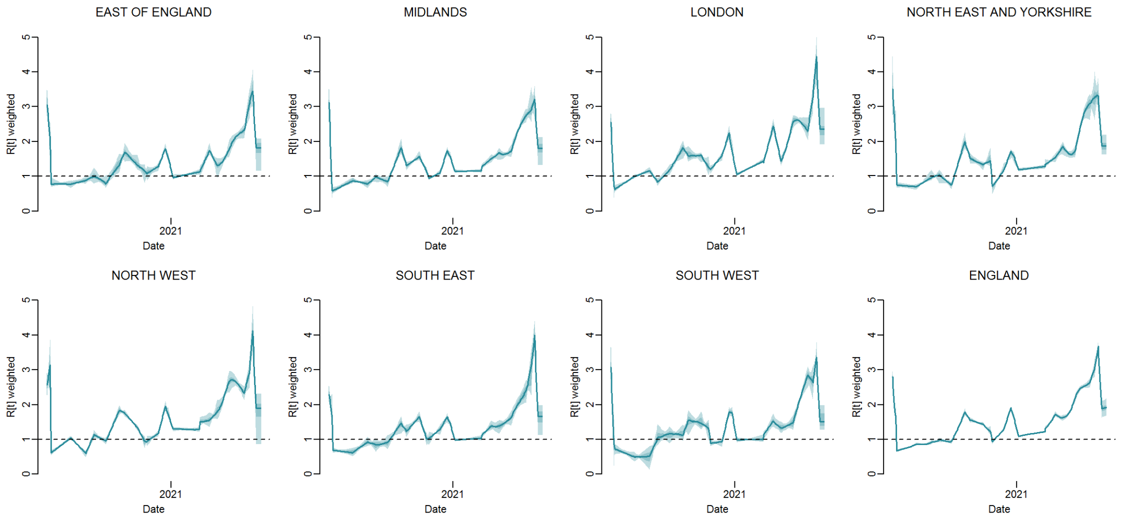

Figure S16: Inferred value of $R^j(t)$ by region over time. The solid line shows the median model fit and the shaded area shows the 95% CrI.

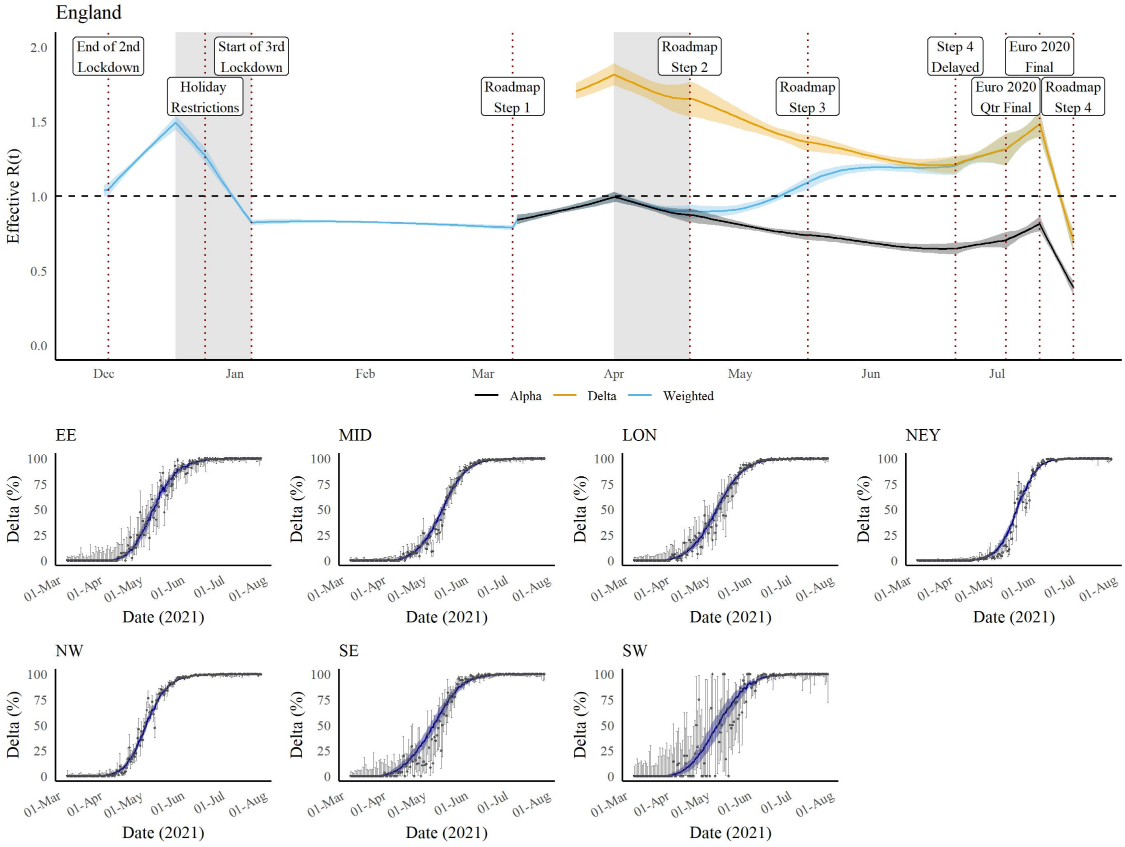

Figure S17: Trajectory of the COVID-19 epidemic in England and the emergence of the Delta variant under central assumptions. (A) Prevalence weighted (blue) and variant-specific (Alpha = black, Delta = orange) effective reproduction number over time from the end of the second national lockdown to 19 July 2021. The solid line shows the median and the shaded area the 95% CrI. The vertical dashed lines show key dates of the roadmap steps and the shaded area the school holidays. (B-H) Model fit to the proportion of Delta cases (variant and mutation data, VAM) over time from 8 March to 19 July. The points show the data, the bar the 95% CI, the blue line the model fit, and the shaded area the 95% CrI. LON = London; SE = South East; NW = North West; SW = South West; MID = Midlands; EE = East of England; NEY = North East and Yorkshire.

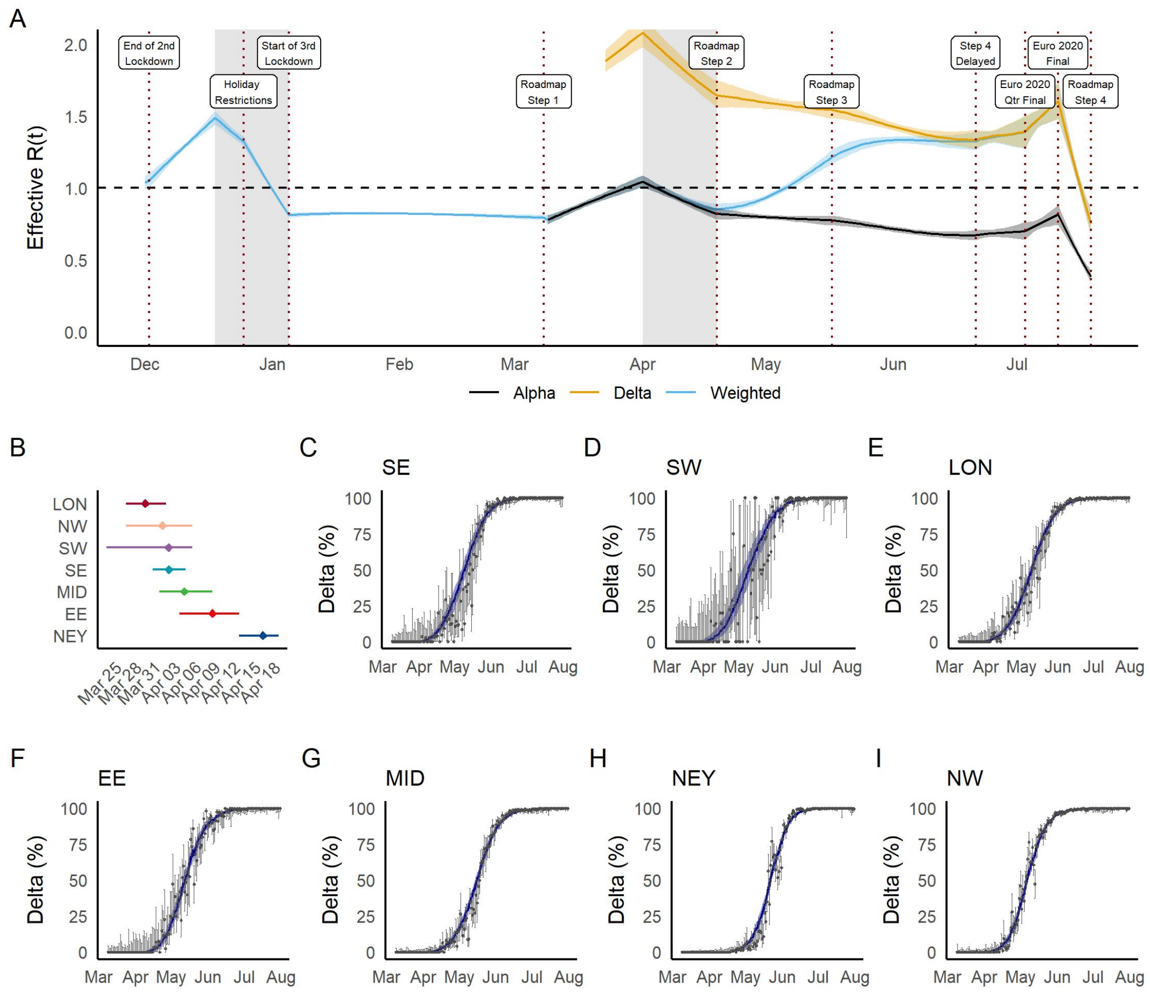

Figure S18: Trajectory of the COVID-19 epidemic in England and the emergence of the Delta variant under optimistic assumptions. (A) Prevalence weighted (blue) and variant-specific (Alpha = black, Delta = orange) effective reproduction number over time from the end of the second national lockdown to 19 July 2021. The solid line shows the median and the shaded area the 95% CrI. The vertical dashed lines show key dates of the roadmap steps and the shaded area the school holidays. (B) Estimated Delta seeding date by NHS region. (C-I) Model fit to the proportion of Delta cases (variant and mutation data, VAM) over time from 8 March to 19 July. The points show the data, the bar the 95% CI, the blue line the model fit, and the shaded area the 95% CrI. LON = London; SE = South East; NW = North West; SW = South West; MID = Midlands; EE = East of England; NEY = North East and Yorkshire.

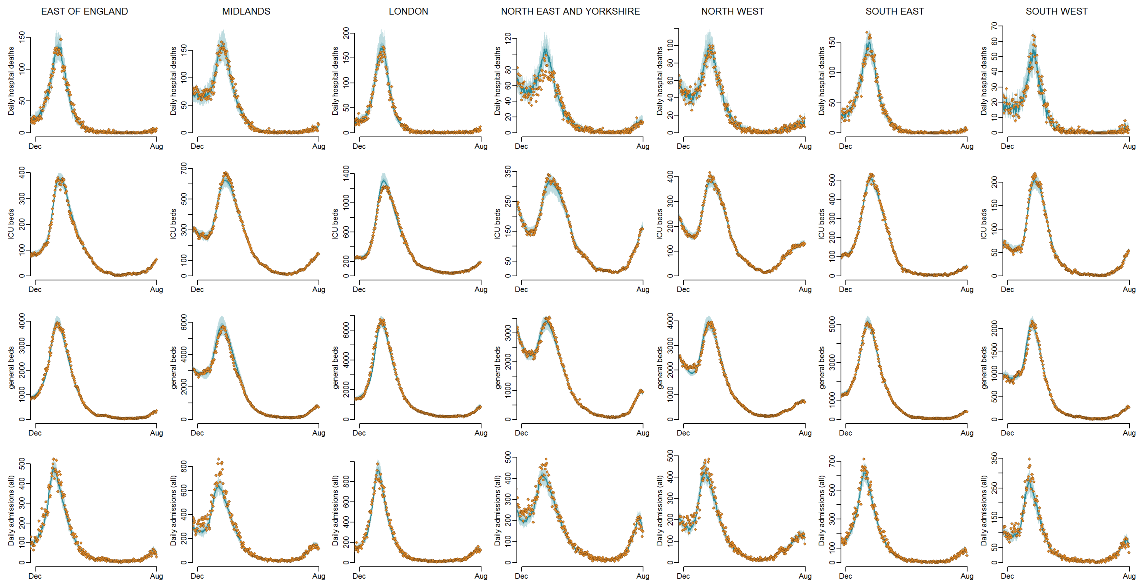

Figure S19: Model fits to England NHS regions: daily hospital deaths (top row), all hospital beds occupancy (second row), and all daily admissions (bottom row) by England NHS region (columns) for the optimistic assumptions. The points show the data, the solid line the median model fit and the shaded area the 95% CrI. Green points indicate data streams that the model does not fit to.

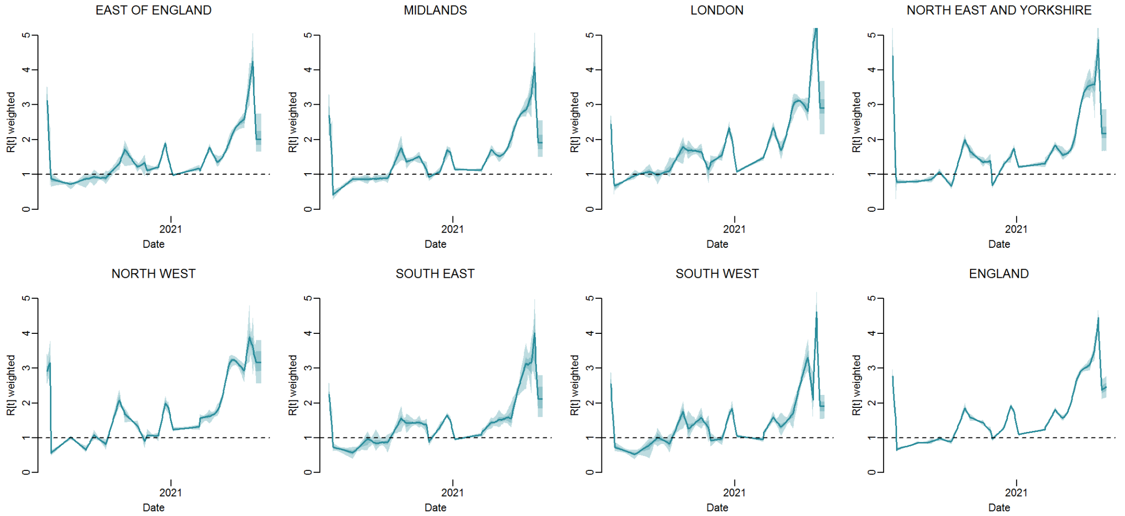

Figure S20: Inferred value of $R^j(t)$ by region over time for the optimistic assumptions. The solid line shows the median model fit and the shaded area shows the 95% CrI.

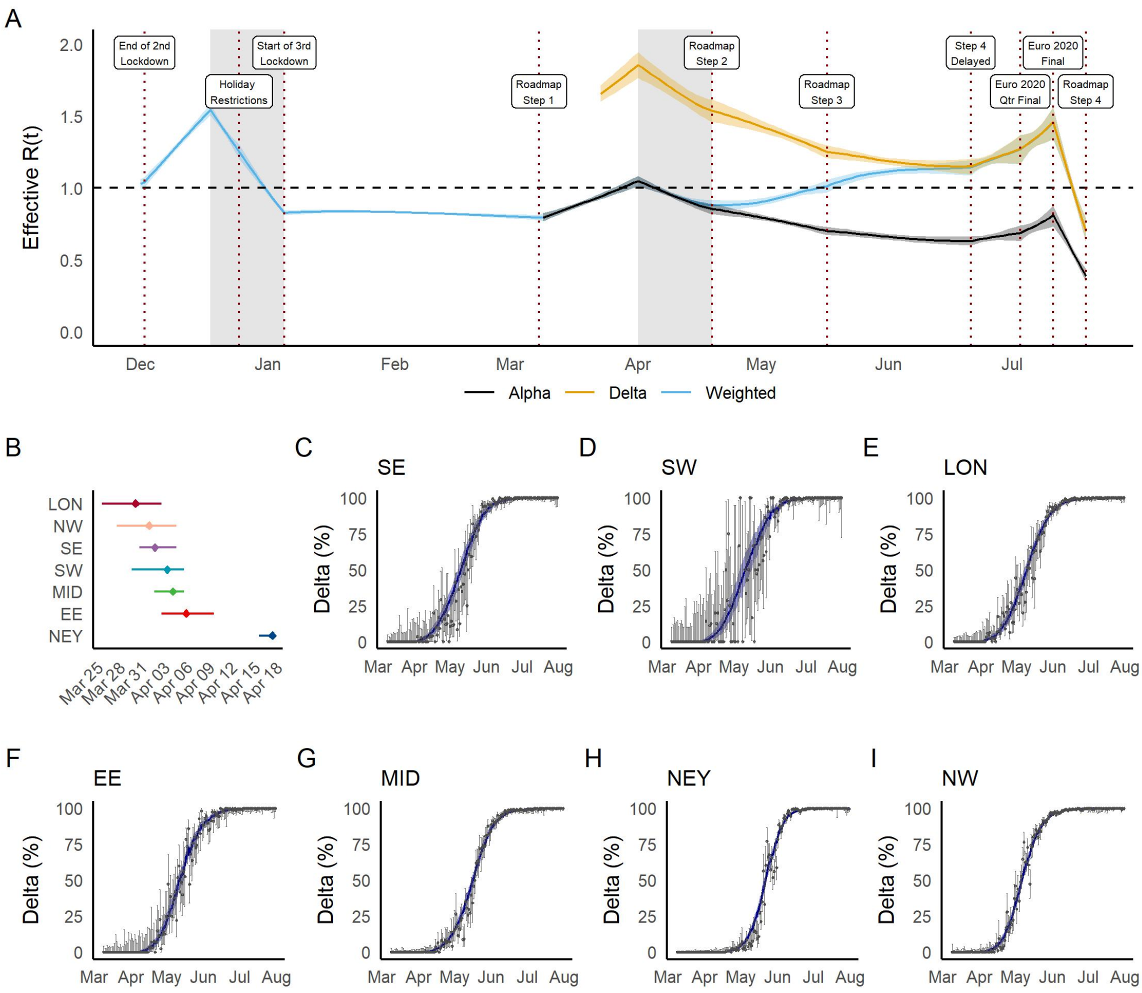

Figure S21: Trajectory of the COVID-19 epidemic in England and the emergence of the Delta variant under pessimistic assumptions. (A) Prevalence weighted (blue) and variant-specific (Alpha = black, Delta = orange) effective reproduction number over time from the end of the second national lockdown to 19 July 2021. The solid line shows the median and the shaded area the 95% CrI. The vertical dashed lines show key dates of the roadmap steps and the shaded area the school holidays. (B) Estimated Delta seeding date by NHS region. (C-I) Model fit to the proportion of Delta cases (variant and mutation data, VAM) over time from 8 March to 19 July. The points show the data, the bar the 95% CI, the blue line the model fit, and the shaded area the 95% CrI. LON = London; SE = South East; NW = North West; SW = South West; MID = Midlands; EE = East of England; NEY = North East and Yorkshire.

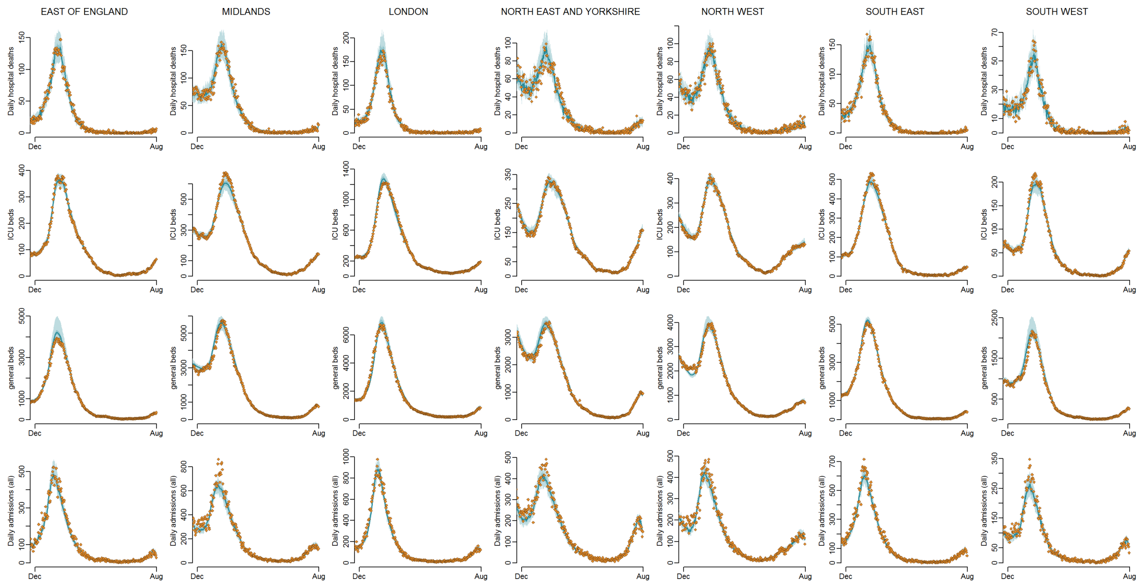

Figure S22: Model fits to England NHS regions: daily hospital deaths (top row), all hospital beds occupancy (second row), and all daily admissions (bottom row) by England NHS region (columns) for the pessimistic assumptions. The points show the data, the solid line the median model fit and the shaded area the 95% CrI. Green points indicate data streams that the model does not fit to.

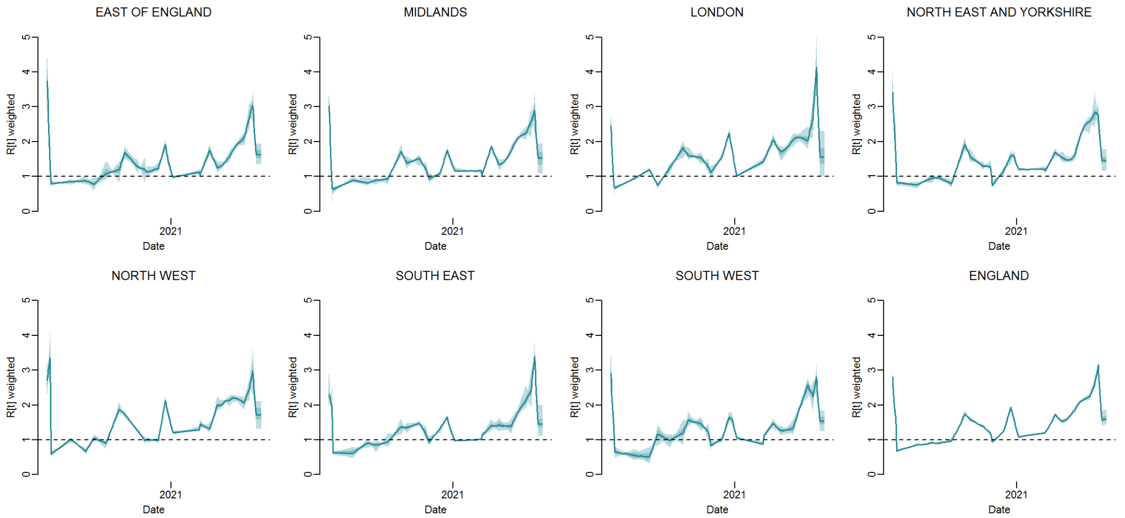

Figure S23: Inferred value of $R^j(t)$ by region over time for the pessimistic assumptions. The solid line shows the median model fit and the shaded area shows the 95% CrI.

7.2 Simulation results

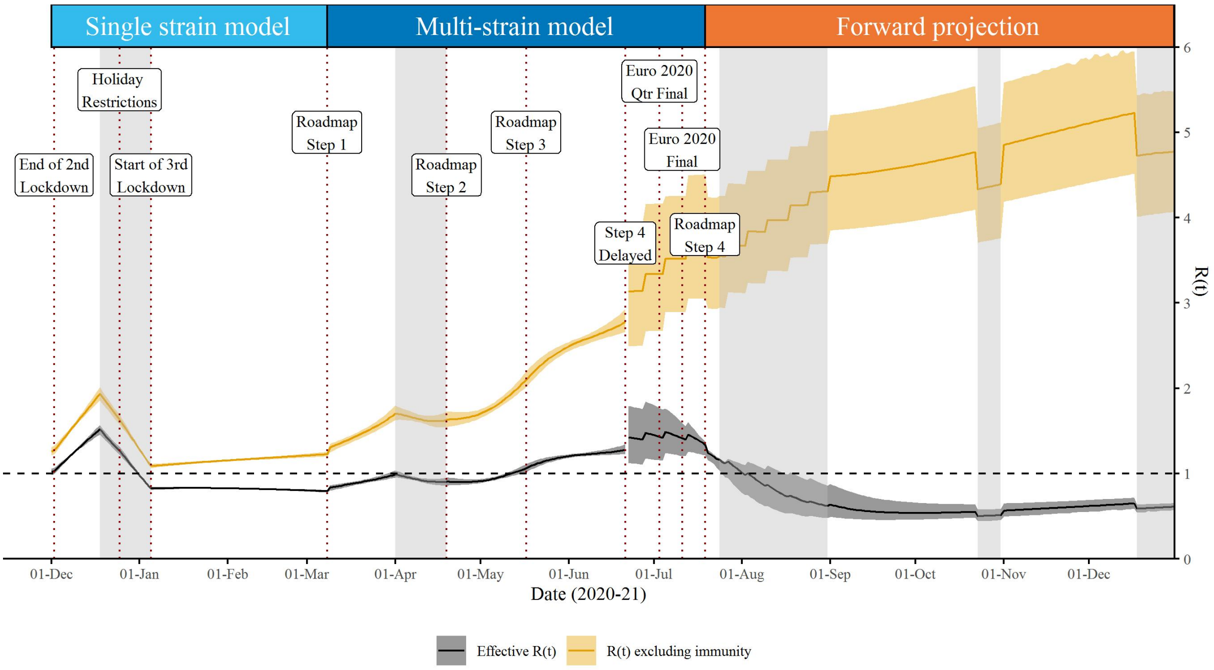

Figure S24: Prevalence weighted (black) effective reproduction number and the reproduction number excluding infection- or vaccine-induced immunity ($R_{\mathrm{excl\_immunity}}$, orange) over time: estimated values from the end of the second national lockdown up to (counterfactual) 21 June 2021 ("roadmap step 4" vertical line) and assumed values thereafter. The solid line shows the R(t) median and the shaded area the 95% CrI. The vertical dashed lines show key dates of the roadmap steps and the shaded area the school holidays. The 'forward projection' section of the figure corresponds to the central immunity scenario.

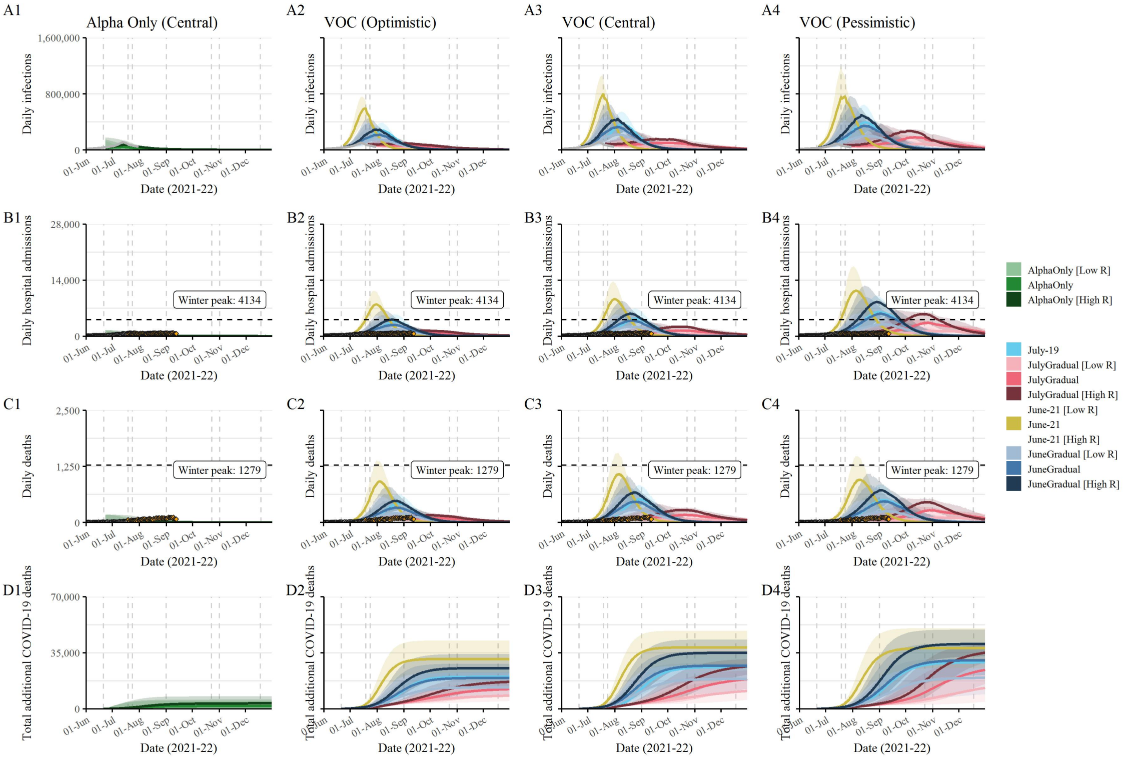

Figure S25: Forward projections for states under different assumptions and scenarios. (A1-D1) counterfactual without seeding of Delta. (A2-D2) scenarios with Delta under optimistic immunity assumptions. (A3-D3) scenarios with Delta under central immunity assumptions. (A4-D4) scenarios with Delta under pessimistic immunity assumptions. (A1-A4) projected daily infections. (B1-B4) projected daily hospital admissions. (C1-C4) projected daily deaths. (D1-D4) projected total COVID-19 deaths up to 11 months after full lift.

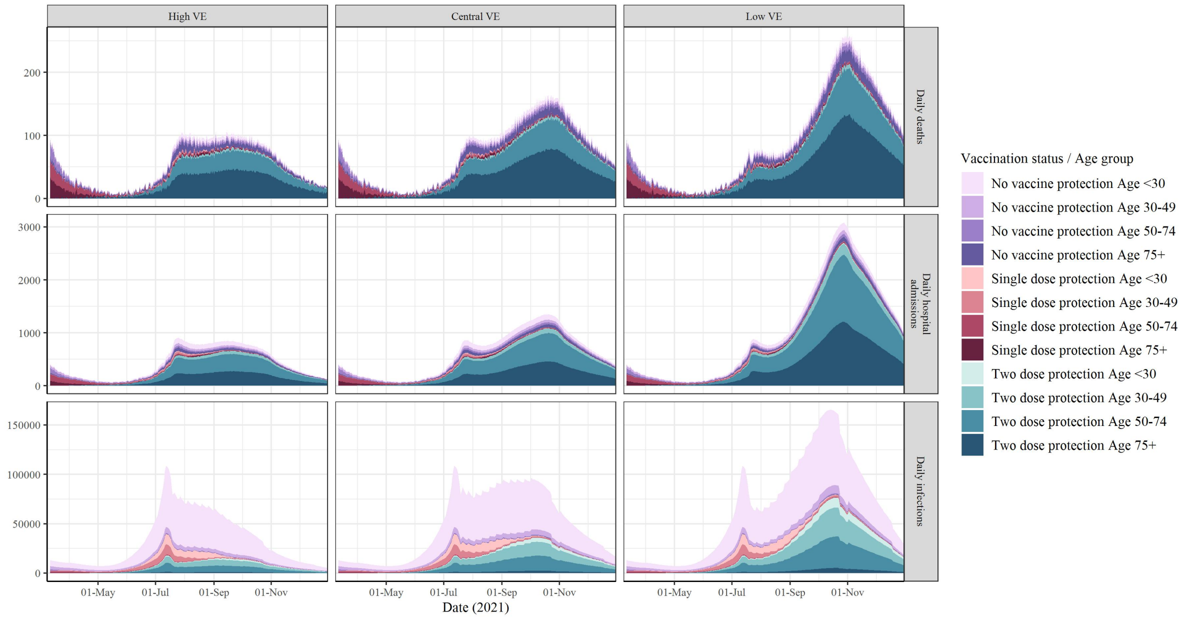

Figure S26: Simulations by age and vaccination status in the 'July-19' scenario. COVID-19 daily deaths (top), hospital admissions (middle), infections (bottom) by age group and vaccination status with optimistic (left), central (middle), and pessimistic (right) assumptions.

Table S17: Peak hospital admissions and deaths, and cumulative infections and deaths (with 95% confidence intervals) in forward projections (to 01 June 2022) across all analyses (column 1), scenarios (column 2), and outcomes (column 3).

Analysis	Scenario	Outcome	Value
AlphaOnly	VOC Central VE	Daily deaths	0 (0, 100)
AlphaOnly	VOC Central VE	Daily hospital admissions	300 (0, 600)
AlphaOnly	VOC Central VE	Daily hospital deaths	0 (0, 100)
AlphaOnly	VOC Central VE	Daily infections	38500 (600, 87300)
AlphaOnly	VOC Central VE	Total additional COVID-19 deaths	1800 (100, 5800)
AlphaOnly	VOC Central VE	Total additional COVID-19 infections	2040700 (83700, 3821200)
AlphaOnly	VOC High VE	Daily deaths	100 (0, 100)
AlphaOnly	VOC High VE	Daily hospital admissions	500 (0, 1000)
AlphaOnly	VOC High VE	Daily hospital deaths	0 (0, 100)
AlphaOnly	VOC High VE	Daily infections	64400 (1700, 130200)
AlphaOnly	VOC High VE	Total additional COVID-19 deaths	2800 (200, 7200)
AlphaOnly	VOC High VE	Total additional COVID-19 infections	2758200 (304400, 3954000)
AlphaOnly	VOC Low VE	Daily deaths	0 (0, 100)
AlphaOnly	VOC Low VE	Daily hospital admissions	100 (0, 500)
AlphaOnly	VOC Low VE	Daily hospital deaths	0 (0, 0)
AlphaOnly	VOC Low VE	Daily infections	15300 (200, 63200)
AlphaOnly	VOC Low VE	Total additional COVID-19 deaths	900 (0, 4600)
AlphaOnly	VOC Low VE	Total additional COVID-19 infections	1192900 (8000, 3279400)
AlphaOnly [High R]	VOC Central VE	Daily deaths	100 (0, 100)
AlphaOnly [High R]	VOC Central VE	Daily hospital admissions	500 (0, 1000)
AlphaOnly [High R]	VOC Central VE	Daily hospital deaths	0 (0, 100)
AlphaOnly [High R]	VOC Central VE	Daily infections	73900 (2000, 123500)
AlphaOnly [High R]	VOC Central VE	Total additional COVID-19 deaths	3600 (1700, 7800)
AlphaOnly [High R]	VOC Central VE	Total additional COVID-19 infections	4214900 (2304700, 5791400)
AlphaOnly [High R]	VOC High VE	Daily deaths	100 (0, 200)
AlphaOnly [High R]	VOC High VE	Daily hospital admissions	800 (0, 1200)
AlphaOnly [High R]	VOC High VE	Daily hospital deaths	100 (0, 100)
AlphaOnly [High R]	VOC High VE	Daily infections	1e+05 (3800, 153900)

Continued on next page

Table S17 – continued from previous page

Analysis	Scenario	Outcome	Value
AlphaOnly [High R]	VOC High VE	Total additional COVID-19 deaths	4800 (1700, 8800)
AlphaOnly [High R]	VOC High VE	Total additional COVID-19 infections	4660300 (2359000, 5787500)
AlphaOnly [High R]	VOC Low VE	Daily deaths	0 (0, 100)
AlphaOnly [High R]	VOC Low VE	Daily deaths	0 (0, 100)
AlphaOnly [High R]	VOC Low VE	Daily hospital admissions	200 (0, 700)
AlphaOnly [High R]	VOC Low VE	Daily hospital deaths	0 (0, 100)
AlphaOnly [High R]	VOC Low VE	Daily infections	33800 (500, 111400)
AlphaOnly [High R]	VOC Low VE	Total additional COVID-19 deaths	2900 (800, 6000)
AlphaOnly [High R]	VOC Low VE	Total additional COVID-19 infections	4000600 (1260100, 5306400)
AlphaOnly [Low R]	VOC Central VE	Daily deaths	0 (0, 200)
AlphaOnly [Low R]	VOC Central VE	Daily hospital admissions	100 (0, 1100)
AlphaOnly [Low R]	VOC Central VE	Daily hospital deaths	0 (0, 100)
AlphaOnly [Low R]	VOC Central VE	Daily infections	22300 (300, 116000)
AlphaOnly [Low R]	VOC Central VE	Total additional COVID-19 deaths	800 (0, 4900)
AlphaOnly [Low R]	VOC Central VE	Total additional COVID-19 infections	880900 (10300, 2465900)
AlphaOnly [Low R]	VOC High VE	Daily deaths	0 (0, 200)
AlphaOnly [Low R]	VOC High VE	Daily hospital admissions	300 (0, 1600)
AlphaOnly [Low R]	VOC High VE	Daily hospital deaths	0 (0, 100)
AlphaOnly [Low R]	VOC High VE	Daily infections	45600 (1100, 139300)
AlphaOnly [Low R]	VOC High VE	Total additional COVID-19 deaths	1600 (0, 6100)
AlphaOnly [Low R]	VOC High VE	Total additional COVID-19 infections	1502400 (38100, 2649800)
AlphaOnly [Low R]	VOC Low VE	Daily deaths	0 (0, 100)
AlphaOnly [Low R]	VOC Low VE	Daily hospital admissions	100 (0, 1000)
AlphaOnly [Low R]	VOC Low VE	Daily hospital deaths	0 (0, 100)
AlphaOnly [Low R]	VOC Low VE	Daily infections	8200 (100, 90800)
AlphaOnly [Low R]	VOC Low VE	Total additional COVID-19 deaths	300 (0, 3600)
AlphaOnly [Low R]	VOC Low VE	Total additional COVID-19 infections	334200 (3000, 2027000)
July-19	VOC Central VE	Daily deaths	500 (300, 800)
July-19	VOC Central VE	Daily hospital admissions	4400 (2700, 6900)
July-19	VOC Central VE	Daily hospital deaths	300 (200, 500)
July-19	VOC Central VE	Daily infections	367900 (240400, 545200)

Continued on next page

Table S17 – continued from previous page

Analysis	Scenario	Outcome	Value
July-19	VOC Central VE	Total additional COVID-19 deaths	27100 (19300, 36500)
July-19	VOC Central VE	Total additional COVID-19 infections	14841600 (11414700, 18783500)
July-19	VOC High VE	Daily deaths	400 (300, 600)
July-19	VOC High VE	Daily hospital admissions	3700 (2400, 5400)
July-19	VOC High VE	Daily hospital deaths	300 (200, 400)
July-19	VOC High VE	Daily infections	276000 (194900, 388000)
July-19	VOC High VE	Total additional COVID-19 deaths	21000 (15500, 26800)
July-19	VOC High VE	Total additional COVID-19 infections	9981500 (7826100, 12273300)
July-19	VOC Low VE	Daily deaths	500 (300, 800)
July-19	VOC Low VE	Daily hospital admissions	6100 (2800, 10100)
July-19	VOC Low VE	Daily hospital deaths	300 (200, 600)
July-19	VOC Low VE	Daily infections	392800 (204500, 634500)
July-19	VOC Low VE	Total additional COVID-19 deaths	30100 (22200, 40400)
July-19	VOC Low VE	Total additional COVID-19 infections	18787800 (13883900, 24222000)
July-19 [High R]	VOC Central VE	Daily deaths	900 (600, 1200)
July-19 [High R]	VOC Central VE	Daily hospital admissions	7600 (5300, 10000)
July-19 [High R]	VOC Central VE	Daily hospital deaths	600 (400, 800)
July-19 [High R]	VOC Central VE	Daily infections	576800 (431600, 733600)
July-19 [High R]	VOC Central VE	Total additional COVID-19 deaths	38600 (29600, 47100)
July-19 [High R]	VOC Central VE	Total additional COVID-19 infections	19316700 (16357000, 22338200)
July-19 [High R]	VOC High VE	Daily deaths	700 (500, 900)
July-19 [High R]	VOC High VE	Daily hospital admissions	6200 (4400, 8000)
July-19 [High R]	VOC High VE	Daily hospital deaths	400 (300, 600)
July-19 [High R]	VOC High VE	Daily infections	416800 (317500, 523900)
July-19 [High R]	VOC High VE	Total additional COVID-19 deaths	29400 (22900, 36000)
July-19 [High R]	VOC High VE	Total additional COVID-19 infections	12839700 (10788300, 14657100)
July-19 [High R]	VOC Low VE	Daily deaths	900 (600, 1200)
July-19 [High R]	VOC Low VE	Daily hospital admissions	10900 (7000, 15200)
July-19 [High R]	VOC Low VE	Daily hospital deaths	600 (400, 800)
July-19 [High R]	VOC Low VE	Daily infections	645700 (427300, 897100)
July-19 [High R]	VOC Low VE	Total additional COVID-19 deaths	42300 (34300, 52000)

Continued on next page

Table S17 – continued from previous page			
Analysis	Scenario	Outcome	Value
July-19 [High R]	VOC Low VE	Total additional COVID-19 infections	24165900 (19939500, 28600200)
July-19 [Low R]	VOC Central VE	Daily deaths	200 (100, 500)
July-19 [Low R]	VOC Central VE	Daily hospital admissions	2100 (1100, 4000)
July-19 [Low R]	VOC Central VE	Daily hospital deaths	200 (100, 300)
July-19 [Low R]	VOC Central VE	Daily infections	198800 (111800, 349900)
July-19 [Low R]	VOC Central VE	Total additional COVID-19 deaths	17000 (10800, 25800)
July-19 [Low R]	VOC Central VE	Total additional COVID-19 infections	10037800 (6549500, 14457500)
July-19 [Low R]	VOC High VE	Daily deaths	200 (100, 400)
July-19 [Low R]	VOC High VE	Daily hospital admissions	1800 (1100, 3300)
July-19 [Low R]	VOC High VE	Daily hospital deaths	100 (100, 200)
July-19 [Low R]	VOC High VE	Daily infections	158300 (100700, 258700)
July-19 [Low R]	VOC High VE	Total additional COVID-19 deaths	13500 (9100, 18900)
July-19 [Low R]	VOC High VE	Total additional COVID-19 infections	6830700 (4597400, 9489500)
July-19 [Low R]	VOC Low VE	Daily deaths	200 (100, 500)
July-19 [Low R]	VOC Low VE	Daily hospital admissions	2400 (900, 5500)
July-19 [Low R]	VOC Low VE	Daily hospital deaths	100 (0, 300)
July-19 [Low R]	VOC Low VE	Daily infections	178300 (75900, 384000)
July-19 [Low R]	VOC Low VE	Total additional COVID-19 deaths	18500 (11700, 28300)
July-19 [Low R]	VOC Low VE	Total additional COVID-19 infections	12514100 (7416400, 18777200)
JulyGradual	VOC Central VE	Daily deaths	200 (100, 200)
JulyGradual	VOC Central VE	Daily hospital admissions	1400 (700, 1700)
JulyGradual	VOC Central VE	Daily hospital deaths	100 (0, 100)
JulyGradual	VOC Central VE	Daily infections	101600 (47000, 117500)
JulyGradual	VOC Central VE	Total additional COVID-19 deaths	21900 (17400, 26100)
JulyGradual	VOC Central VE	Total additional COVID-19 infections	12139300 (10464900, 14231700)
JulyGradual	VOC High VE	Daily deaths	100 (100, 100)
JulyGradual	VOC High VE	Daily hospital admissions	900 (800, 1000)
JulyGradual	VOC High VE	Daily hospital deaths	100 (0, 100)
JulyGradual	VOC High VE	Daily hospital deaths	100 (0, 100)
JulyGradual	VOC High VE	Daily hospital deaths	100 (0, 100)
JulyGradual	VOC High VE	Daily infections	79800 (63900, 106700)

Continued on next page

Table S17 – continued from previous page

Analysis	Scenario	Outcome	Value
JulyGradual	VOC High VE	Total additional COVID-19 deaths	14200 (11300, 17400)
JulyGradual	VOC High VE	Total additional COVID-19 infections	6936200 (5539700, 8463000)
JulyGradual	VOC Low VE	Daily deaths	300 (100, 300)
JulyGradual	VOC Low VE	Daily hospital admissions	3300 (1200, 3800)
JulyGradual	VOC Low VE	Daily hospital deaths	200 (100, 200)
JulyGradual	VOC Low VE	Daily infections	179100 (69600, 199800)
JulyGradual	VOC Low VE	Total additional COVID-19 deaths	28900 (24300, 34400)
JulyGradual	VOC Low VE	Total additional COVID-19 infections	17499900 (14618200, 20863100)
JulyGradual [High R]	VOC Central VE	Daily deaths	300 (200, 300)
JulyGradual [High R]	VOC Central VE	Daily hospital admissions	2400 (1700, 2600)
JulyGradual [High R]	VOC Central VE	Daily hospital deaths	200 (100, 200)
JulyGradual [High R]	VOC Central VE	Daily infections	157800 (83900, 179800)
JulyGradual [High R]	VOC Central VE	Total additional COVID-19 deaths	29200 (25000, 33100)
JulyGradual [High R]	VOC Central VE	Total additional COVID-19 infections	15047300 (13735100, 16839400)
JulyGradual [High R]	VOC High VE	Daily deaths	200 (100, 200)
JulyGradual [High R]	VOC High VE	Daily hospital admissions	1400 (700, 1700)
JulyGradual [High R]	VOC High VE	Daily hospital deaths	100 (0, 100)
JulyGradual [High R]	VOC High VE	Daily infections	96400 (45800, 132800)
JulyGradual [High R]	VOC High VE	Total additional COVID-19 deaths	18700 (16200, 21800)
JulyGradual [High R]	VOC High VE	Total additional COVID-19 infections	8729700 (7670000, 9957000)
JulyGradual [High R]	VOC Low VE	Daily deaths	500 (300, 500)
JulyGradual [High R]	VOC Low VE	Daily hospital admissions	5500 (3100, 6100)
JulyGradual [High R]	VOC Low VE	Daily hospital deaths	300 (200, 400)
JulyGradual [High R]	VOC Low VE	Daily infections	271300 (142800, 292900)
JulyGradual [High R]	VOC Low VE	Total additional COVID-19 deaths	38300 (33600, 42600)
JulyGradual [High R]	VOC Low VE	Total additional COVID-19 infections	21477500 (19068200, 24371600)
JulyGradual [Low R]	VOC Central VE	Daily deaths	100 (100, 100)
JulyGradual [Low R]	VOC Central VE	Daily hospital admissions	900 (800, 1000)
JulyGradual [Low R]	VOC Central VE	Daily hospital deaths	100 (0, 100)
JulyGradual [Low R]	VOC Central VE	Daily infections	81700 (60200, 116000)
JulyGradual [Low R]	VOC Central VE	Total additional COVID-19 deaths	14900 (10000, 19500)

Continued on next page

Table S17 – continued from previous page

Analysis	Scenario	Outcome	Value
JulyGradual [Low R]	VOC Central VE	Total additional COVID-19 infections	8848800 (6021500, 11440500)
JulyGradual [Low R]	VOC High VE	Daily deaths	100 (100, 100)
JulyGradual [Low R]	VOC High VE	Daily hospital admissions	900 (800, 1000)
JulyGradual [Low R]	VOC High VE	Daily hospital deaths	100 (0, 100)
JulyGradual [Low R]	VOC High VE	Daily infections	77600 (61100, 104100)
JulyGradual [Low R]	VOC High VE	Total additional COVID-19 deaths	9700 (6900, 13600)
JulyGradual [Low R]	VOC High VE	Total additional COVID-19 infections	4771700 (3209700, 6938800)
JulyGradual [Low R]	VOC Low VE	Daily deaths	100 (0, 200)
JulyGradual [Low R]	VOC Low VE	Daily hospital admissions	1300 (200, 2100)
JulyGradual [Low R]	VOC Low VE	Daily hospital deaths	100 (0, 100)
JulyGradual [Low R]	VOC Low VE	Daily infections	85500 (13800, 107600)
JulyGradual [Low R]	VOC Low VE	Total additional COVID-19 deaths	19400 (13300, 25600)
JulyGradual [Low R]	VOC Low VE	Total additional COVID-19 infections	12876600 (8531100, 17002300)
June-21	VOC Central VE	Daily deaths	1100 (600, 1500)
June-21	VOC Central VE	Daily hospital admissions	9300 (5400, 13400)
June-21	VOC Central VE	Daily hospital deaths	700 (400, 1000)
June-21	VOC Central VE	Daily infections	795200 (495100, 1030500)
June-21	VOC Central VE	Total additional COVID-19 deaths	38300 (27000, 48800)
June-21	VOC Central VE	Total additional COVID-19 infections	21772600 (16744300, 25921300)
June-21	VOC High VE	Daily deaths	900 (600, 1300)
June-21	VOC High VE	Daily hospital admissions	8000 (5100, 11100)
June-21	VOC High VE	Daily hospital deaths	600 (400, 800)
June-21	VOC High VE	Daily infections	590500 (407500, 717700)
June-21	VOC High VE	Total additional COVID-19 deaths	31100 (22400, 42600)
June-21	VOC High VE	Total additional COVID-19 infections	15648200 (12570600, 18538900)
June-21	VOC Low VE	Daily deaths	1000 (500, 1500)
June-21	VOC Low VE	Daily hospital admissions	11400 (5600, 17000)
June-21	VOC Low VE	Daily hospital deaths	600 (300, 1000)
June-21	VOC Low VE	Daily infections	764500 (425500, 1061800)
June-21	VOC Low VE	Total additional COVID-19 deaths	38000 (25600, 50400)
June-21	VOC Low VE	Total additional COVID-19 infections	24333200 (17725100, 30144400)

Continued on next page

Table S17 – continued from previous page			
Analysis	Scenario	Outcome	Value
June-21 [High R]	VOC Central VE	Daily deaths	1700 (1200, 2200)
June-21 [High R]	VOC Central VE	Daily hospital admissions	14700 (10300, 18700)
June-21 [High R]	VOC Central VE	Daily hospital deaths	1100 (800, 1400)
June-21 [High R]	VOC Central VE	Daily infections	1100600 (798000, 1310600)
June-21 [High R]	VOC Central VE	Total additional COVID-19 deaths	53400 (41700, 64000)
June-21 [High R]	VOC Central VE	Total additional COVID-19 infections	26690000 (22657400, 29994900)
June-21 [High R]	VOC High VE	Daily deaths	1500 (1100, 1900)
June-21 [High R]	VOC High VE	Daily hospital admissions	12600 (9400, 15800)
June-21 [High R]	VOC High VE	Daily hospital deaths	900 (700, 1100)
June-21 [High R]	VOC High VE	Daily infections	812500 (629800, 929300)
June-21 [High R]	VOC High VE	Total additional COVID-19 deaths	43800 (35000, 56000)
June-21 [High R]	VOC High VE	Total additional COVID-19 infections	19100900 (16625000, 21326600)
June-21 [High R]	VOC Low VE	Daily deaths	1600 (1100, 2100)
June-21 [High R]	VOC Low VE	Daily hospital admissions	19200 (12500, 24700)
June-21 [High R]	VOC Low VE	Daily hospital deaths	1100 (700, 1400)
June-21 [High R]	VOC Low VE	Daily infections	1196000 (825300, 1514000)
June-21 [High R]	VOC Low VE	Total additional COVID-19 deaths	53600 (42100, 65100)
June-21 [High R]	VOC Low VE	Total additional COVID-19 infections	30312700 (25606600, 34854400)
June-21 [Low R]	VOC Central VE	Daily deaths	500 (200, 900)
June-21 [Low R]	VOC Central VE	Daily hospital admissions	4400 (1900, 8100)
June-21 [Low R]	VOC Central VE	Daily hospital deaths	300 (100, 600)
June-21 [Low R]	VOC Central VE	Daily infections	429700 (199200, 725800)
June-21 [Low R]	VOC Central VE	Total additional COVID-19 deaths	22800 (12700, 33300)
June-21 [Low R]	VOC Central VE	Total additional COVID-19 infections	15148300 (9441300, 20807900)
June-21 [Low R]	VOC High VE	Daily deaths	500 (200, 800)
June-21 [Low R]	VOC High VE	Daily hospital admissions	3900 (1900, 6900)
June-21 [Low R]	VOC High VE	Daily hospital deaths	300 (100, 500)
June-21 [Low R]	VOC High VE	Daily infections	344200 (183500, 550500)
June-21 [Low R]	VOC High VE	Total additional COVID-19 deaths	18500 (11700, 29800)
June-21 [Low R]	VOC High VE	Total additional COVID-19 infections	11103400 (7410900, 15001900)
June-21 [Low R]	VOC Low VE	Daily deaths	400 (200, 900)

Continued on next page

Table S17 – continued from previous page

Analysis	Scenario	Outcome	Value
June-21 [Low R]	VOC Low VE	Daily hospital admissions	4900 (1800, 10200)
June-21 [Low R]	VOC Low VE	Daily hospital deaths	300 (100, 600)
June-21 [Low R]	VOC Low VE	Daily infections	373300 (164300, 772800)
June-21 [Low R]	VOC Low VE	Total additional COVID-19 deaths	22000 (12100, 35200)
June-21 [Low R]	VOC Low VE	Total additional COVID-19 infections	16259000 (9604800, 24135400)
JuneGradual	VOC Central VE	Daily deaths	500 (200, 700)
JuneGradual	VOC Central VE	Daily hospital admissions	3900 (1500, 5700)
JuneGradual	VOC Central VE	Daily hospital deaths	300 (100, 400)
JuneGradual	VOC Central VE	Daily infections	331100 (133300, 451100)
JuneGradual	VOC Central VE	Total additional COVID-19 deaths	27000 (18500, 35900)
JuneGradual	VOC Central VE	Total additional COVID-19 infections	16812600 (12426200, 21463000)
JuneGradual	VOC High VE	Daily deaths	300 (100, 500)
JuneGradual	VOC High VE	Daily hospital admissions	2800 (1300, 3900)
JuneGradual	VOC High VE	Daily hospital deaths	200 (100, 300)
JuneGradual	VOC High VE	Daily infections	219400 (102700, 279800)
JuneGradual	VOC High VE	Total additional COVID-19 deaths	19300 (13500, 28200)
JuneGradual	VOC High VE	Total additional COVID-19 infections	11158400 (8430900, 14379500)
JuneGradual	VOC Low VE	Daily deaths	500 (200, 600)
JuneGradual	VOC Low VE	Daily hospital admissions	5600 (1900, 6700)
JuneGradual	VOC Low VE	Daily hospital deaths	300 (100, 400)
JuneGradual	VOC Low VE	Daily infections	342700 (125500, 395800)
JuneGradual	VOC Low VE	Total additional COVID-19 deaths	30500 (21600, 40800)
JuneGradual	VOC Low VE	Total additional COVID-19 infections	20367200 (15160400, 26135500)
JuneGradual [High R]	VOC Central VE	Daily deaths	700 (300, 900)
JuneGradual [High R]	VOC Central VE	Daily hospital admissions	5600 (2700, 6900)
JuneGradual [High R]	VOC Central VE	Daily hospital deaths	400 (200, 600)
JuneGradual [High R]	VOC Central VE	Daily infections	443000 (221900, 507500)
JuneGradual [High R]	VOC Central VE	Total additional COVID-19 deaths	35000 (27000, 43300)
JuneGradual [High R]	VOC Central VE	Total additional COVID-19 infections	20108300 (16498500, 23764700)
JuneGradual [High R]	VOC High VE	Daily deaths	500 (300, 700)
JuneGradual [High R]	VOC High VE	Daily hospital admissions	4100 (2300, 5300)

Continued on next page

Table S17 – continued from previous page

Analysis	Scenario	Outcome	Value
JuneGradual [High R]	VOC High VE	Daily hospital deaths	300 (200, 400)
JuneGradual [High R]	VOC High VE	Daily infections	292500 (170600, 326200)
JuneGradual [High R]	VOC High VE	Total additional COVID-19 deaths	25400 (19900, 34200)
JuneGradual [High R]	VOC High VE	Total additional COVID-19 infections	13389200 (11115000, 16056500)
JuneGradual [High R]	VOC Low VE	Daily deaths	700 (300, 900)
JuneGradual [High R]	VOC Low VE	Daily hospital admissions	8600 (3700, 10000)
JuneGradual [High R]	VOC Low VE	Daily hospital deaths	500 (200, 600)
JuneGradual [High R]	VOC Low VE	Daily infections	497700 (209100, 602600)
JuneGradual [High R]	VOC Low VE	Total additional COVID-19 deaths	40600 (33000, 49500)
JuneGradual [High R]	VOC Low VE	Total additional COVID-19 infections	24803000 (20701100, 29244600)
JuneGradual [Low R]	VOC Central VE	Daily deaths	300 (100, 500)
JuneGradual [Low R]	VOC Central VE	Daily hospital admissions	2400 (700, 4600)
JuneGradual [Low R]	VOC Central VE	Daily hospital deaths	200 (0, 400)
JuneGradual [Low R]	VOC Central VE	Daily infections	221200 (70200, 387000)
JuneGradual [Low R]	VOC Central VE	Total additional COVID-19 deaths	18700 (10600, 28400)
JuneGradual [Low R]	VOC Central VE	Total additional COVID-19 infections	12874000 (7963700, 18622500)
JuneGradual [Low R]	VOC High VE	Daily deaths	200 (100, 400)
JuneGradual [Low R]	VOC High VE	Daily hospital admissions	1800 (600, 3700)
JuneGradual [Low R]	VOC High VE	Daily hospital deaths	100 (0, 200)
JuneGradual [Low R]	VOC High VE	Daily infections	149800 (55200, 242300)
JuneGradual [Low R]	VOC High VE	Total additional COVID-19 deaths	13400 (8300, 22700)
JuneGradual [Low R]	VOC High VE	Total additional COVID-19 infections	8603300 (5456300, 12501700)
JuneGradual [Low R]	VOC Low VE	Daily deaths	200 (0, 400)
JuneGradual [Low R]	VOC Low VE	Daily hospital admissions	3000 (600, 4600)
JuneGradual [Low R]	VOC Low VE	Daily hospital deaths	200 (0, 300)
JuneGradual [Low R]	VOC Low VE	Daily infections	202800 (46700, 316600)
JuneGradual [Low R]	VOC Low VE	Total additional COVID-19 deaths	19700 (11500, 31400)
JuneGradual [Low R]	VOC Low VE	Total additional COVID-19 infections	14708300 (9182000, 22246700)

7.3 Sensitivity analyses

The results of the sensitivity analyses are computed with Shapley values [56]. Shapley values are rooted in game theory and treat each variable as 'players' in a 'game' that contribute towards the final simulation. In our analyses we use Shapley values to firstly calculate the importance of each 'level' of a feature, where a level is a possible value the feature can take (e.g. optimistic/central/pessimistic for VE), and secondly to describe the overall importance of the feature. In the simplest explanation, a Shapley value is the expected increase or decrease in a projection caused by the variable (or level) of interest. For example, in Figure S27, the 'July-19 [High R]' scenario is expected to result in 19,697 more deaths than other scenarios, where 'other scenarios' is the expected number of deaths in all scenarios except 'July-19 [High R]'. To derive a measure of variable importance, we take the expectation of the absolute Shapley value across all levels in a variable, such as in Figure S28. Here, variables are ordered according to importance (top is most important and bottom is least) and the numbers correspond to the absolute expected difference. These plots provide a measure of variable importance by comparing the magnitude of the expected difference, e.g. the level of mixing (Scenario) is more important than waning rate as it can lead to an over 7-fold difference in number of expected deaths.

To demonstrate how these are computed, take the bottom-left cell as an example in which we compute the Shapley value for the 'July-19 [High R]' scenario on the total additional COVID-19 deaths, this is computed as follows, let

- f_{St} be a simulation for a given state, St ('Total deaths'), from our fitted model with all variables as described previously with different combinations of the variables listed below;
- SC_i be the ith level of the scenario variable ('July-19 [High R]');
- VE_j be the jth level of the vaccine effectiveness variable, e.g. 'optimistic';
- CI_k be the kth level of the cross immunity variable, e.g. 'optimistic';
- WR_l be the lth level of the waning rate variable, e.g. 'optimistic';
- ϕ_{v_i} be the Shapley value for the ith level of the vth variable (Shapley for 'July-19 [High R]' of the scenario variable);
- n_v be the number of levels in the variable of interest, v ($n_v = 6$);
- n'_v be the total number of levels in all variables except v ($n_{v'} = 27$).

Then for the scenario variable:

$$\phi_{v_i} = \frac{1}{n_v - 1} \sum_{i \neq i'} \frac{1}{n_{v'}} \sum_{j,k,l} f_{St}(SC'_i, VE_j, CI_k, WR_l) - f_{St}(SC_i, VE_j, CI_k, WR_l) \Big|_{v=SC} \qquad (255)$$

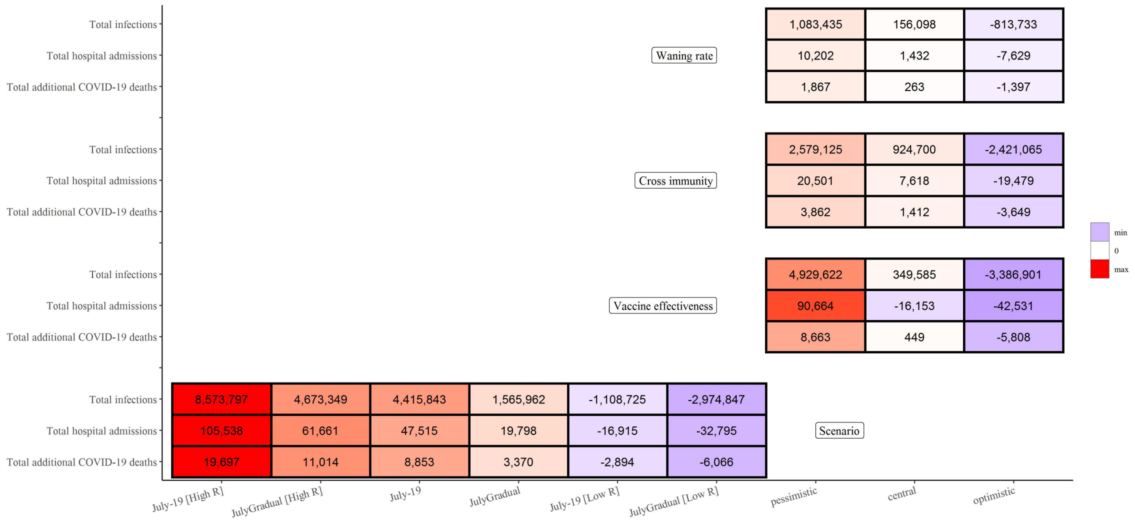

Figure S27: Shapley values computed from three different simulated states (total deaths, admissions, and hospitalisations) on 1 June 2022 from the model fit with 'central' assumptions. Shapley values are computed as the expected difference in the measured state within variables. For example, the bottom left cell indicates that the July-19 [High R] scenario is expected to lead to 19,697 more deaths than other scenarios.

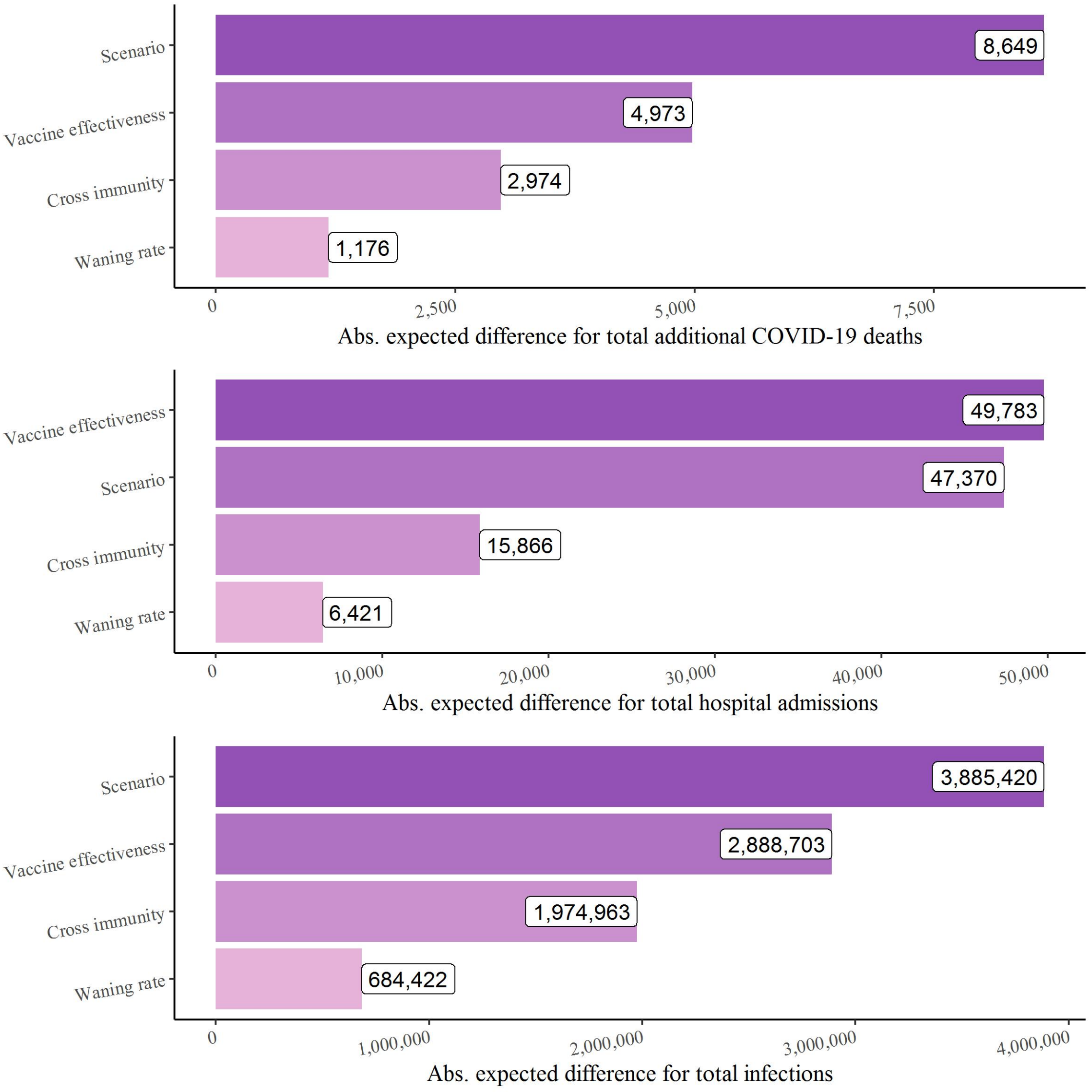

Figure S28: Absolute expected Shapley values for the model with central assumptions for total additional COVID-19 deaths (top), hospital admissions (middle), and COVID-19 infections (bottom) by 1 June 2022 (counted from 19 July 2021). Each plot is ordered from most important variable (top) to least (bottom), according to the expected absolute Shapley value with expectations taken over feature levels.

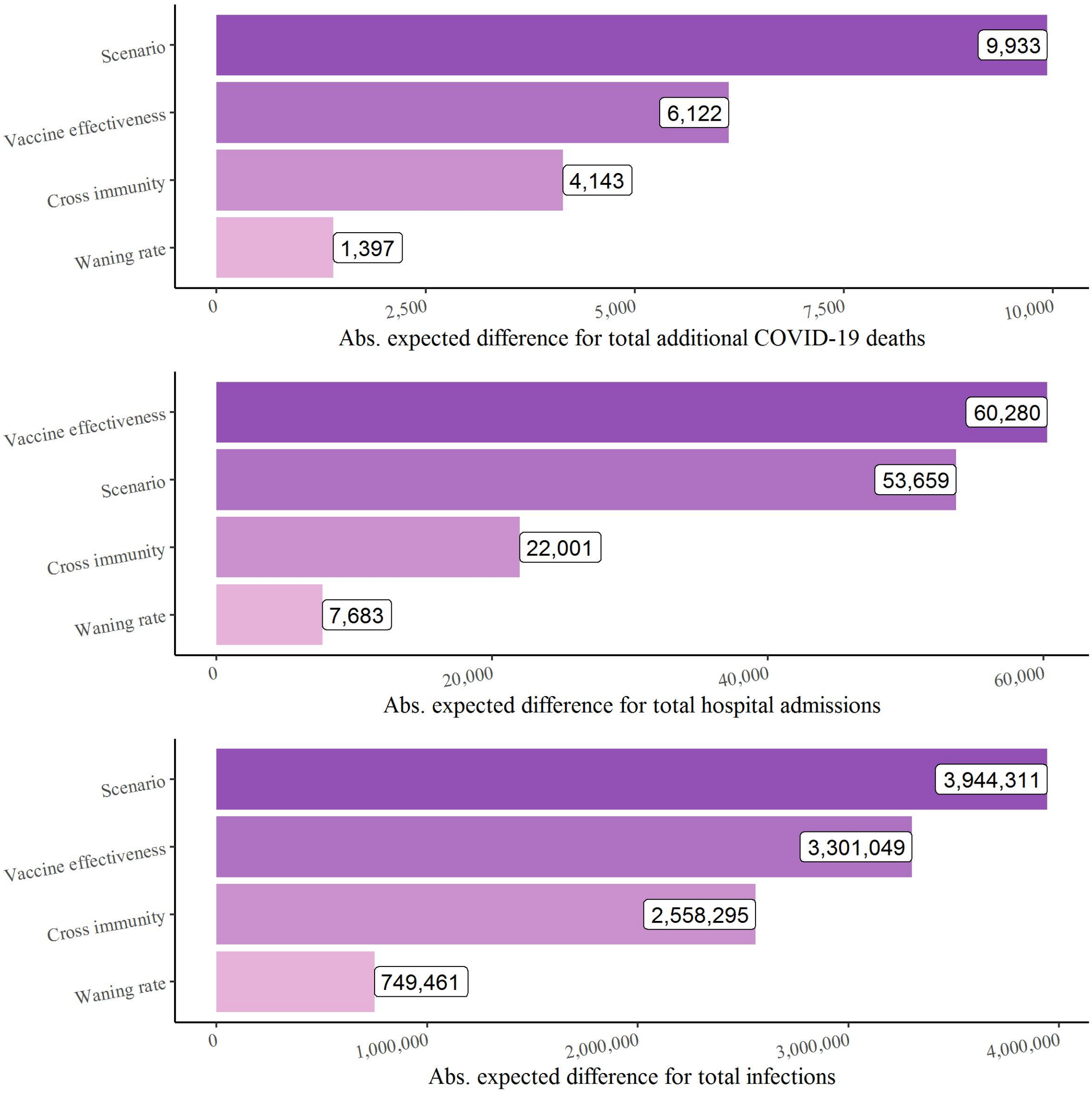

Figure S29: Absolute expected Shapley values for the model with optimistic assumptions for total additional COVID-19 deaths (top), hospital admissions (middle), and COVID-19 infections (bottom) by 1 June 2022 (counted from 19 July 2021). Each plot is ordered from most important variable (top) to least (bottom), according to the expected absolute Shapley value with expectations taken over feature levels.

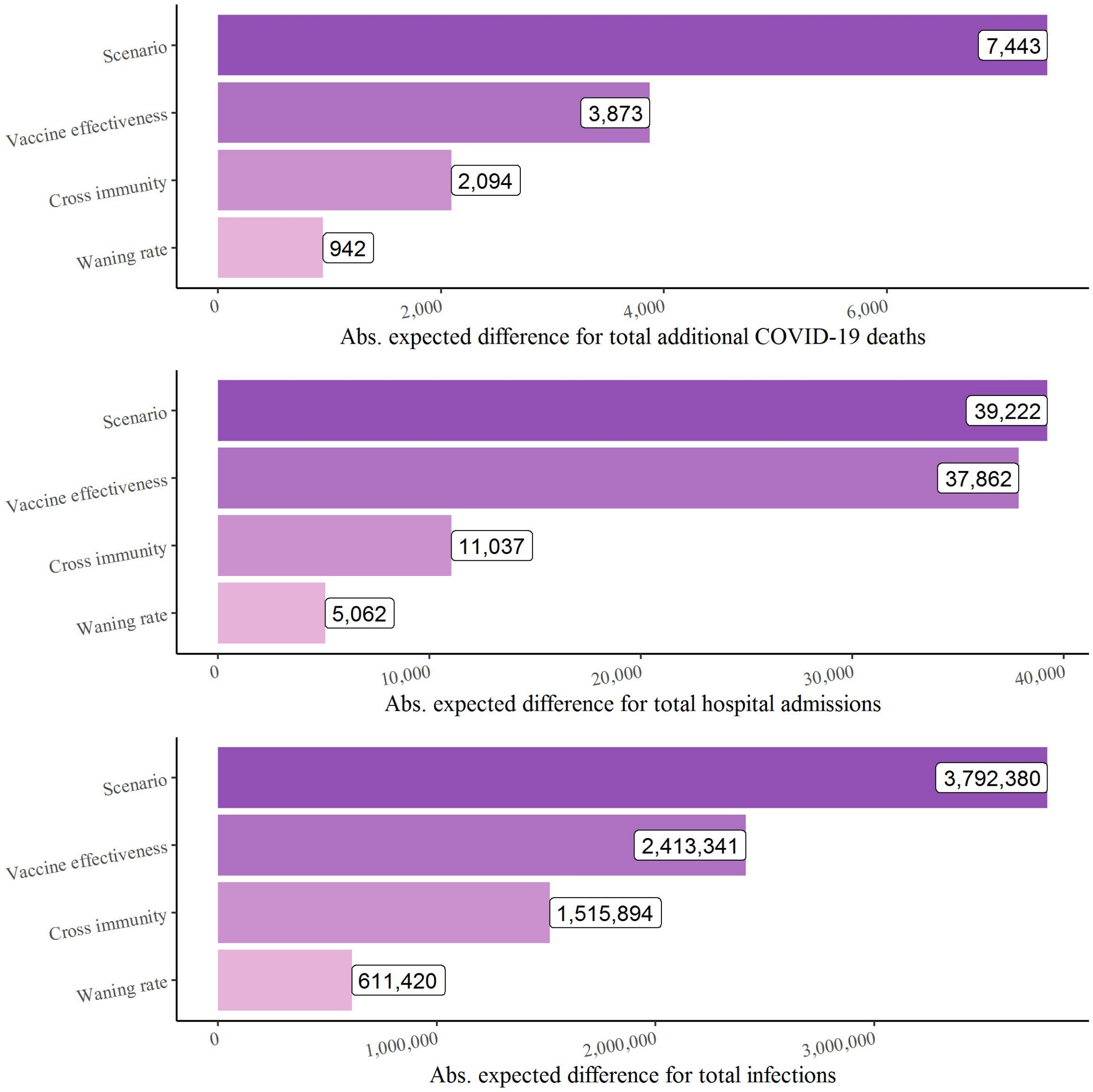

Figure S30: Absolute expected Shapley values for the model with pessimistic assumptions for total additional COVID-19 deaths (top), hospital admissions (middle), and COVID-19 infections (bottom) by 1 June 2022 (counted from 19 July 2021). Each plot is ordered from most important variable (top) to least (bottom), according to the expected absolute Shapley value with expectations taken over feature levels.

7.4 Waning vaccine effectiveness simulations

The implementation of VE waning is described in section 3.5.

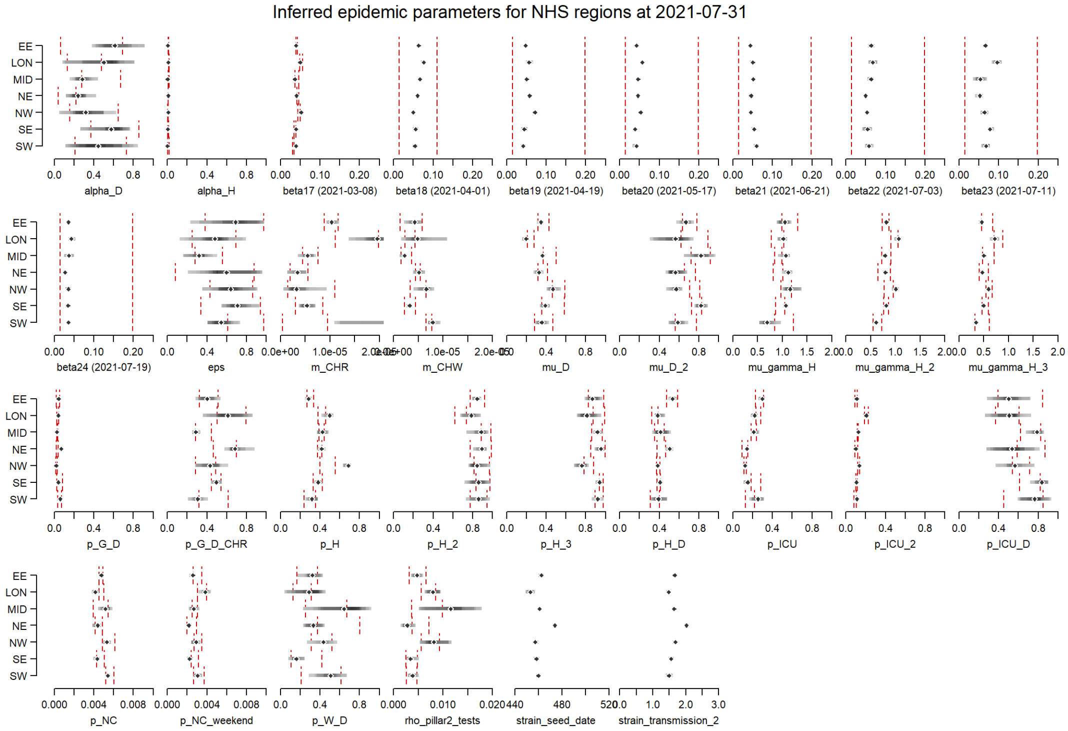

Figure S31: Model parameter posterior distributions assuming waning of VE. Red bars indicate prior distribution range, black bars indicate the 95% CrI.

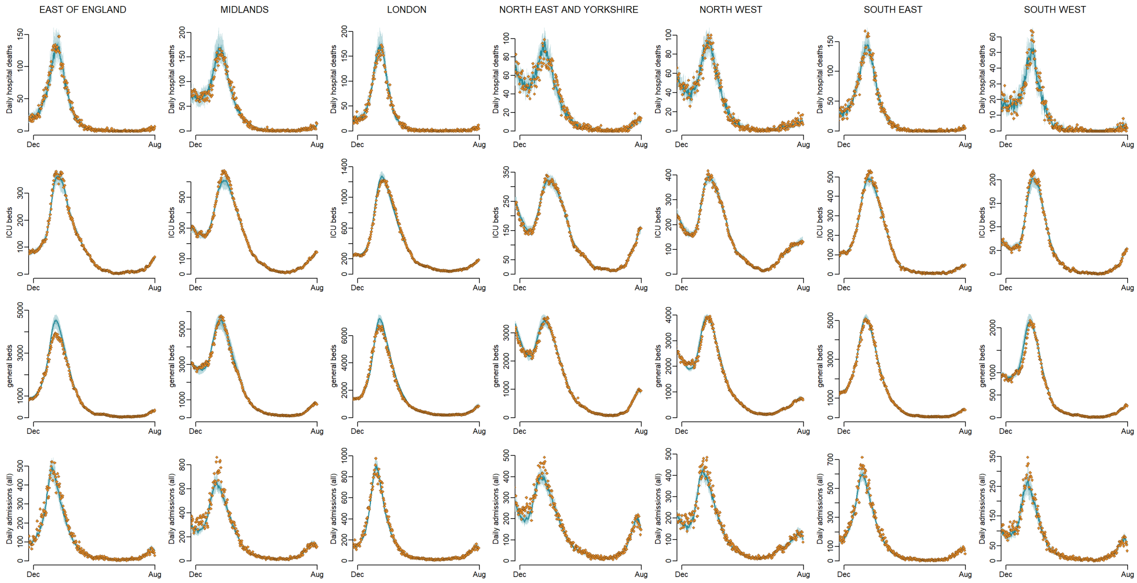

Figure S32: Model fits (assuming waning of VE) to England NHS regions: daily hospital deaths (top row), all hospital beds occupancy (second row), and all daily admissions (bottom row) by England NHS region (columns). The points show the data, the solid line the median model fit and the shaded area the 95% CrI.

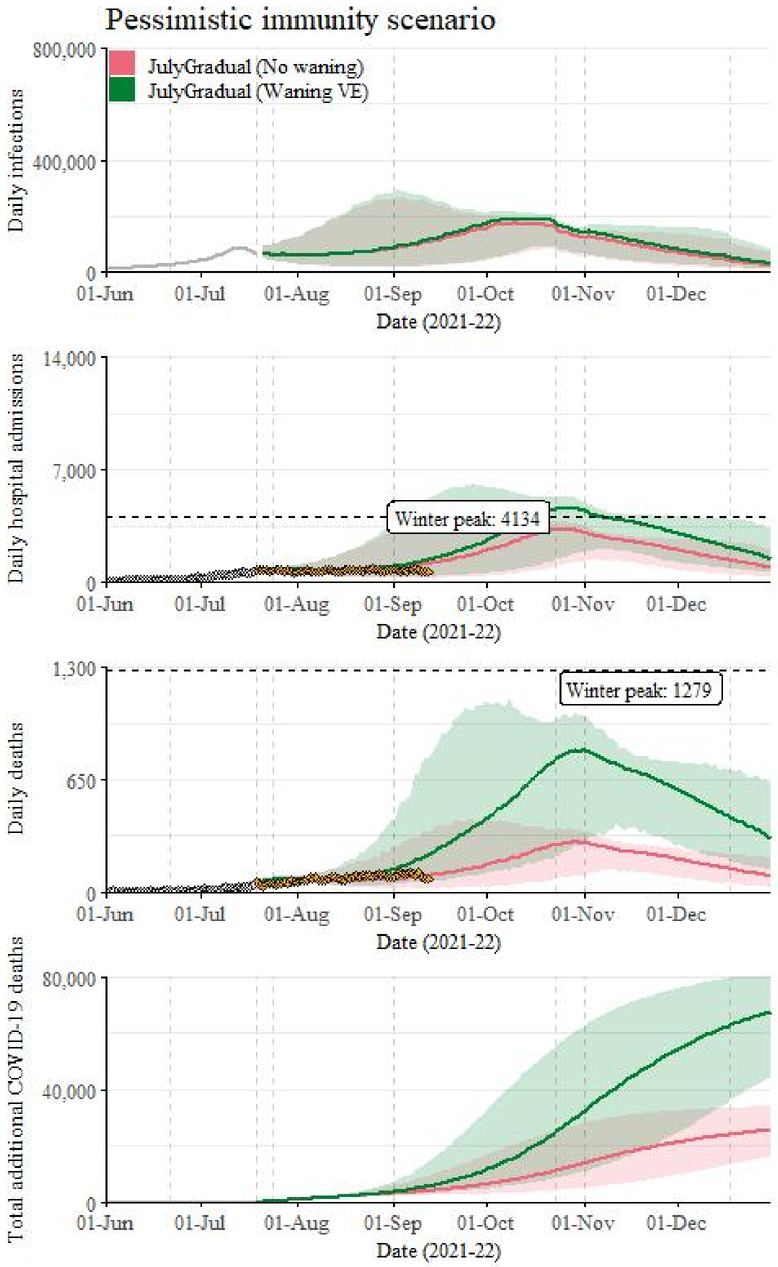

Figure S33: Daily infections (top), hospital admissions (second row), hospital deaths (third row), and cumulative deaths to 1 January 2021 (bottom) for the 'gradual' July full lift central R and pessimistic vaccine assumptions with no waning of vaccine immunity (red) and with waning of vaccine immunity (green). Coloured lines are medians, with shaded areas showing the 95% credible intervals. White points are the observed data used for fitting, orange points are observed data not used in fitting, dark grey lines are fitted trajectories from the waning VE scenario (fitted trajectories from non-waning VE scenario are very similar to waning VE scenario, see Figure 3). Note that y-axis limits are the same as in Figure 3, except for cumulative deaths which is extended from 60,000 to 80,000. The central projected trend is the median across all daily projections and is not a single trajectory.

Symbol Glossary

Symbol	Definition
	**Abbreviations**
CHW	Carehome workers
CHR	Carehome residents
VE	Vaccine effectiveness
AZ	AstraZeneca ChAdOx1 nCoV-19 (AZD1222) vaccine
PF	Pfizer-BioNTech COVID-19 Vaccine BNT162b2
Mod	Moderna mRNA-1273 vaccine
	**Model Compartments**
S^{ik}	Susceptible
$E^{i,j,k}$	Exposed
$I_P^{i,j,k}$	Infected pre-symptomatic
$I_A^{i,j,k}$	Infected aymptomatic
$I_{C_1}^{i,j,k}$	Symptomatic infected (infectious)
$I_{C_2}^{i,j,k}$	Symptomatic infected (not infectious)
$G_D^{i,j,k}$	Severe disease, not hospitalised
$D^{i,j,k}$	Deceased (as a result of COVID-19)
$R^{i,j,k}$	Recovered
V_k	Vaccination strata
$ICU_{pre}^{i,j,k}$	Awaiting admission to ICU
$ICU_{W_R}^{i,j,k}$	Hospitalised in ICU, leading to recovery
$ICU_{W_D}^{i,j,k}$	Hospitalised in ICU, leading to death following step-down from ICU
$ICU_D^{i,j,k}$	Hospitalised in ICU, leading to death
$W_D^{i,j,k}$	Step-down post-ICU period, leading to death
$W_R^{i,j,k}$	Step-down recovery period
$H_D^{i,j,k}$	Hospitalised on general ward leading to death
$H_R^{i,j,k}$	Hospitalised on general ward leading to recovery
	**Model Parameters**
$p_H^{i,j,k}(t)$	Probability of hospitalisation given symptomatic
$p_{G_D}^{i,j,k}$	Probability of severe disease but not hospitalised
$p_{ICU}^{i}(t)$	Probability of ICU admission given hospitalised
$p_{H_D}^{i,j,k}(t)$	Probability of death given hospitalised and not in ICU
$p_{ICU_D}^{i,j,k}(t)$	Probability of death given ICU
$p_{W_D}^{i,j,k}(t)$	Probability of death after discharge
$\chi^{i,j,k}(t)$	Susceptibility of an individual to variant j
$\xi^{i,j,k}(t)$	Infectivity of an individual infected with variant j
$\lambda^{i,j,k}(t)$	Variant-specific force of infection
$\Lambda^{i,k}(t)$	Combined force of infection (both variants)
$\zeta^{i,k}(t)$	Rate of progression from vaccine strata k to $k+1$
Continued on next page	

**Table S18 – continued from previous page**	
Symbol	Definition
γ_x	Rate of progression from compartment x
$R^j(t)$	R number for variant j at time t
$R_e^j(t)$	Effective R number for variant j at time t
t_0	Regional outbreak start date
t_{Delta}	Delta seeding date
σ	Delta transmission advantage
$m_{i,i'}(t)$	Person-to-person transmission rate
$c_{i,j}$	Person-to-person contact rate
$\beta(t)$	Transmission rate
β_i	Transmission rate at change-point t_i
$\Theta_{i,j,k}(t)$	Weighted number of infectious individuals
ε	Relative reduction in contacts between CHR and general population
$\Delta_I^{i,k}$	Mean duration of infectiousness weighted by infectivity
$\hat{\sigma}(t)$	Ratio of effective reproduction number for Alpha and Delta ($R^{Alpha}(t)$ and $R^{Delta}(t)$).
**Vaccine Effectiveness vs.**	
e_{inf}	Infection
e_{sympt}	Symptoms
e_{SD}	Severe disease
e_{death}	Death
$e_{sympt\|inf}$	Symptoms given infection
$e_{SD\|sympt}$	Severe disease given symptoms
$e_{death\|SD}$	Death given severe disease
e_{ins}	Infectiousness
**Fixed Parameters**	
$p_C^{i,j,k}$	Probability of being symptomatic given infected
$p^*(t)$	Probability of COVID-19 diagnosis confirmed prior to hospital admission
γ_U	Rate at which unconfirmed hospital patients are confirmed as infected
γ_R	Rate at which natural immunity from infection wanes
$p_{sero_{pos}}$	Probability of seroconversion following infection
$p_{sero_{spec}}$	Specificity of serology test
$p_{sero_{sens}}$	Sensitivity of serology test
$1/\gamma_{sero_{pre}}$	Mean time to seroconversion from onset of infectiousness
$1/\gamma_{sero^1_{pos}}$	Mean duration of seropositivity (Euroimmun assay)
$1/\gamma_{sero^2_{pos}}$	Mean duration of seropositivity (Roche N)
η	Proportion of cross-immunity to Delta following infection from Alpha
θ_{I_A}	Infectivity of an asymptomatic individual, relative to a symptomatic one

List of Figures

S1 Compartmental model diagram . . . 4
S2 Vaccination strata . . . 8
S3 Cited VE from the literature, compared against our assumptions . . . 10
S4 Vaccine uptake by type and age . . . 11
S5 Cumulative vaccine uptake by age . . . 13
S6 Compartmental model diagram . . . 15
S7 PCR and seropositivity structure . . . 18
S8 Daily seasonality multiplier . . . 45
S9 β posterior distributions (central) . . . 49
S10 Posterior distributions (central) . . . 50
S11 Pillar 2 model fits by region (central) . . . 51
S12 REACT-1 model fits by region (central) . . . 51
S13 Model fits by region to EuroImmun serology assay (central) . . . 52
S14 Model fits by region to Roche serology assay (central) . . . 52
S15 Model fits to data streams (central) . . . 53
S16 Inferred $R^j(t)$ by region over time (central) . . . 53
S17 Epidemic trajectory with Delta (central) . . . 54
S18 Epidemic trajectory with Delta (optimistic) . . . 55
S19 Model fits to data streams (optimistic) . . . 56
S20 Inferred $R^j(t)$ by region over time (optimistic) . . . 56
S21 Epidemic trajectory with Delta (pessimistic) . . . 57
S22 Model fits to data streams (pessimistic) . . . 58
S23 Inferred $R^j(t)$ by region over time (pessimistic) . . . 58
S24 Fitted and simulated $R(t)$ in June counterfactual . . . 59
S25 Simulation state trajectories with and without Delta . . . 60
S26 Simulations by age and vaccination status . . . 61
S27 Full sensitivity analysis shapley values . . . 72
S28 Absolute expected Shapley values (central) . . . 73
S29 Absolute expected Shapley values (optimistic) . . . 74
S30 Absolute expected Shapley values (pessimistic) . . . 75
S31 Posterior distributions (with VE waning) . . . 76
S32 Model fits to data streams (with VE waning) . . . 77
S33 Comparing impact of VE waning . . . 78

List of Tables

S1	Data sources and definitions	6
S2	Vaccination strata	8
S3	Vaccine effectiveness assumptions	9
S4	Conditional vaccine effectiveness values	12
S5	Waned vaccine effectiveness	14
S6	Definitions of Figure S1 compartments	16
S7	Definitions of Figure S1 parameters	17
S8	Fixed model parameters	38
S10	Model parameter combinations	42
S11	Forward projection scenarios	43
S12	School term dates	44
S13	Onward vaccine uptake assumptions	46
S14	Projected simulation scenarios	46
S15	Gradual July scenario $R^{Alpha}(t)$ schedule	47
S16	Gradual June scenario $R^{Alpha}(t)$ schedule	47
S17	Peak deaths, infections and admissions in forward projections	62

References

[1] Knock ES, Whittles LK, Lees JA, Perez-Guzman PN, Verity R, FitzJohn RG, et al. Key epidemiological drivers and impact of interventions in the 2020 SARS-CoV-2 epidemic in England. Science Translational Medicine. 2021;13(602). Available from: https://stm.sciencemag.org/content/13/602/eabg4262.

[2] Mossong J, Hens N, Jit M, Beutels P, Auranen K, Mikolajczyk R, et al. Social contacts and mixing patterns relevant to the spread of infectious diseases. PLoS Med. 2008;5(3):e74.

[3] Regulatory approval of COVID-19 Vaccine AstraZeneca;. Accessed: 2021-05-20. https://www.gov.uk/government/publications/regulatory-approval-of-covid-19-vaccine-astrazeneca.

[4] Regulatory approval of Pfizer/BioNTech vaccine for COVID-19;. Accessed: 2021-05-20. https://www.gov.uk/government/publications/regulatory-approval-of-pfizer-biontech-vaccine-for-covid-19.

[5] Moderna vaccine becomes third COVID-19 vaccine approved by UK regulator;. Accessed: 2021-05-28. https://www.gov.uk/government/news/moderna-vaccine-becomes-third-covid-19-vaccine-approved-by-uk-regulator.

[6] Coronavirus (COVID-19) in the UK;. Accessed: 2021-05-20. https://coronavirus.data.gov.uk.

[7] Riley S, Walters CE, Wang H, Eales O, Ainslie KE, Atchinson C, et al. REACT-1 round 7 updated report: regional heterogeneity in changes in prevalence of SARS-CoV-2 infection during the second national COVID-19 lockdown in England. medRxiv. 2020.

[8] Sero-surveillance of COVID-19;. Accessed: 2021-05-20. https://www.gov.uk/government/publications/national-covid-19-surveillance-reports/sero-surveillance-of-covid-19.

[9] Population estimates by output areas, electoral, health and other geographies, England and Wales: mid-2020;. Accessed: 2021-09-20. https://www.ons.gov.uk/peoplepopulationandcommunity/populationandmigration/populationestimates/bulletins/annualsmallareapopulationestimates/mid2020.

[10] Pung R, Mak TM, Kucharski AJ, Lee VJ, working group CC, et al. Serial intervals observed in SARS-CoV-2 B. 1.617. 2 variant cases. medRxiv. 2021.

[11] Sheikh A, McMenamin J, Taylor B, Robertson C. SARS-CoV-2 Delta VOC in Scotland: demographics, risk of hospital admission, and vaccine effectiveness. The Lancet. 2021.

[12] Edara VV, Lai L, Sahoo M, Floyd K, Sibai M, Solis D, et al. Infection and vaccine-induced neutralizing antibody responses to the SARS-CoV-2 B. 1.617. 1 variant. bioRxiv. 2021.

[13] COVID-19 vaccination programme: FAQs on second doses;. Accessed: 2021-05-20. https://www.england.nhs.uk/coronavirus/publication/covid-19-vaccination-programme-faqs-on-second-doses/.

[14] Mulligan MJ, Lyke KE, Kitchin N, Absalon J, Gurtman A, Lockhart S, et al. Phase I/II study of COVID-19 RNA vaccine BNT162b1 in adults. Nature. 2020;586(7830):589–593.

[15] Ramasamy MN, Minassian AM, Ewer KJ, Flaxman AL, Folegatti PM, Owens DR, et al. Safety and immunogenicity of ChAdOx1 nCoV-19 vaccine administered in a prime-boost regimen in young

and old adults (COV002): a single-blind, randomised, controlled, phase 2/3 trial. The Lancet. 2020;396(10267):1979–1993.

[16] Voysey M, Clemens SAC, Madhi SA, Weckx LY, Folegatti PM, Aley PK, et al. Safety and efficacy of the ChAdOx1 nCoV-19 vaccine (AZD1222) against SARS-CoV-2: an interim analysis of four randomised controlled trials in Brazil, South Africa, and the UK. The Lancet. 2021;397(10269):99–111.

[17] Polack FP, Thomas SJ, Kitchin N, Absalon J, Gurtman A, Lockhart S, et al. Safety and efficacy of the BNT162b2 mRNA Covid-19 vaccine. New England Journal of Medicine. 2020;383(27):2603–2615.

[18] Harris RJ, Hall JA, Zaidi A, Andrews NJ, Dunbar JK, Dabrera G. Impact of vaccination on household transmission of SARS-COV-2 in England. medRxiv. 2021.

[19] Bernal JL, Andrews N, Gower C, Robertson C, Stowe J, Tessier E, et al. Effectiveness of the Pfizer-BioNTech and Oxford-AstraZeneca vaccines on covid-19 related symptoms, hospital admissions, and mortality in older adults in England: test negative case-control study. bmj. 2021;373.

[20] Bernal JL, Andrews N, Gower C, Stowe J, Tessier E, Simmons R, et al. Effectiveness of BNT162b2 mRNA vaccine and ChAdOx1 adenovirus vector vaccine on mortality following COVID-19. medRxiv. 2021.

[21] Ismail S, Vilaplana T, Elgohari S, et al.. Effectiveness of BNT162b2 mRNA and ChAdOx1 adenovirus vector COVID-19 vaccines on risk of hospitalisation among older adults in England: an observational study using surveillance data; 2021.

[22] Vasileiou E, Simpson CR, Shi T, Kerr S, Agrawal U, Akbari A, et al. Interim findings from first-dose mass COVID-19 vaccination roll-out and COVID-19 hospital admissions in Scotland: a national prospective cohort study. The Lancet. 2021.

[23] Public Health England vaccine effectiveness report;. Accessed: 2021-05-20. `https://assets.publishing.service.gov.uk/government/uploads/system/uploads/attachment_data/file/971017/SP_PH__VE_report_20210317_CC_JLB.pdf`.

[24] Hyams C, Marlow R, Maseko Z, King J, Ward L, Fox K, et al. Assessing the effectiveness of bnt162b2 and chadox1ncov-19 covid-19 vaccination in prevention of hospitalisations in elderly and frail adults: A single centre test negative case-control study. SSRN Electronic Journal. 2021.

[25] Stowe J, Andrews N, Gower C, Gallagher E, Utsi L, Simmons R. Effectiveness of COVID-19 vaccines against hospital admission with the Delta (B. 1.617. 2) variant. Public Health England. 2021.

[26] Hall VJ, Foulkes S, Saei A, Andrews N, Oguti B, Charlett A, et al. Effectiveness of BNT162b2 mRNA vaccine against infection and COVID-19 vaccine coverage in healthcare workers in England, multicentre prospective cohort study (the SIREN Study). SSRN Electronic Journal. 2021.

[27] Voysey M, Clemens SAC, Madhi SA, Weckx LY, Folegatti PM, Aley PK, et al. Single-dose administration and the influence of the timing of the booster dose on immunogenicity and efficacy of ChAdOx1 nCoV-19 (AZD1222) vaccine: a pooled analysis of four randomised trials. The Lancet. 2021;397(10277):881–891.

[28] Bernal JL, Andrews N, Gower C, Gallagher E, Simmons R, Thelwall S, et al. Effectiveness of Covid-19 vaccines against the B. 1.617. 2 (Delta) variant. New England Journal of Medicine. 2021;385(7):385–394.

[29] Nasreen S, He S, Chung H, Brown KA, Gubbay JB, Buchan SA, et al. Effectiveness of COVID-19 vaccines against variants of concern, Canada. medRxiv. 2021.

[30] Decline in Vaccine Effectiveness Against Infection and Symptomatic Illness;. Accessed: 2021-08-04. https://www.gov.il/en/departments/news/05072021-03.

[31] Folegatti PM, Bittaye M, Flaxman A, Lopez FR, Bellamy D, Kupke A, et al. Safety and immunogenicity of a candidate Middle East respiratory syndrome coronavirus viral-vectored vaccine: a dose-escalation, open-label, non-randomised, uncontrolled, phase 1 trial. The Lancet Infectious Diseases. 2020;20(7):816–826.

[32] Coronavirus vaccine rollout;. Accessed: 2021-05-20. https://www.instituteforgovernment.org.uk/explainers/coronavirus-vaccine-rollout.

[33] COVID-19 Greenbook chapter 14a;. Accessed: 2021-06-16. https://assets.publishing.service.gov.uk/government/uploads/system/uploads/attachment_data/file/984310/Greenbook_chapter_14a_7May2021.pdf.

[34] JCVI advice to mitigate impact of B1.617.2 variant;. Accessed: 2021-09-14. https://www.gov.uk/government/news/jcvi-advice-to-mitigate-impact-of-b1-617-2-variant.

[35] Andrews N, Tessier E, Stowe J, Gower C, Kirsebom F, Simmons R, et al. Vaccine effectiveness and duration of protection of Comirnaty, Vaxzevria and Spikevax against mild and severe COVID-19 in the UK. medRxiv. 2020.

[36] Funk S. Socialmixr: Social mixing matrices for infectious disease modelling. 2018.

[37] COVID-19 Hospital Activity;. Accessed: 2021-05-20. https://www.england.nhs.uk/statistics/statistical-work-areas/covid-19-hospital-activity/.

[38] Lauer SA, Grantz KH, Bi Q, Jones FK, Zheng Q, Meredith HR, et al. The incubation period of coronavirus disease 2019 (COVID-19) from publicly reported confirmed cases: estimation and application. Annals of internal medicine. 2020;172(9):577–582.

[39] Gillespie DT. Approximate accelerated stochastic simulation of chemically reacting systems. The Journal of chemical physics. 2001;115(4):1716–1733.

[40] Diekmann O, Heesterbeek JAP, Metz JA. On the definition and the computation of the basic reproduction ratio R 0 in models for infectious diseases in heterogeneous populations. Journal of mathematical biology. 1990;28(4):365–382.

[41] SGSS and CHESS data;. Accessed: 2021-05-20. https://digital.nhs.uk/about-nhs-digital/corporate-information-and-documents/directions-and-data-provision-notices/data-provision-notices-dpns/sgss-and-chess-data.

[42] LSHTM: Interim roadmap assessment – prior to steps 3 and 4, 5 May 2021;. Accessed: 2021-07-27. https://assets.publishing.service.gov.uk/government/uploads/system/uploads/attachment_data/file/984546/S1230_LSHTM_Interim_roadmap_assessment_prior_to_steps_3_and_4.pdf.

[43] Brazeau N, Verity R, Jenks S, Fu H, Whittaker C, Winskill P, et al. Report 34: COVID-19 infection fatality ratio: estimates from seroprevalence. Imperial College London COVID-19 Reports. 2020.

[44] Borremans B, Gamble A, Prager K, Helman SK, McClain AM, Cox C, et al. Quantifying antibody kinetics and RNA detection during early-phase SARS-CoV-2 infection by time since symptom onset. Elife. 2020;9:e60122.

[45] Harris RJ, Whitaker HJ, Andrews NJ, Aiano F, Amin-Chowdhury Z, Flood J, et al. Serological surveillance of SARS-CoV-2: Six-month trends and antibody response in a cohort of public health workers. Journal of Infection. 2021;82(5):162–169.

[46] Chen S, Flegg JA, White LJ, Aguas R. Levels of SARS-CoV-2 population exposure are considerably higher than suggested by seroprevalence surveys. MedRxiv. 2021.

[47] Planas D, Veyer D, Baidaliuk A, Staropoli I, Guivel-Benhassine F, Rajah MM, et al. Reduced sensitivity of SARS-CoV-2 variant Delta to antibody neutralization. Nature. 2021:1–7.

[48] Public Health Scotland COVID-19 Statistical Report As at 28 June 2021;. Accessed: 2021-07-29. `https://www.publichealthscotland.scot/media/8268/21-06-30-covid19-publication_report.pdf`.

[49] Evaluating the Roadmap out of Lockdown: Step 3;. Accessed: 2021-07-27. `https://assets.publishing.service.gov.uk/government/uploads/system/uploads/attachment_data/file/984688/S1228_Imperial_Evaluating_the_Roadmap_out_of_Lockdown_Step_3.pdf`.

[50] Engelbrecht FA, Scholes RJ. Test for Covid-19 seasonality and the risk of second waves. One Health. 2021;12:100202.

[51] Baker RE, Yang W, Vecchi GA, Metcalf CJE, Grenfell BT. Assessing the influence of climate on wintertime SARS-CoV-2 outbreaks. Nature communications. 2021;12(1):1–7.

[52] Evaluating the Roadmap out of Lockdown for England: modelling the delayed step 4 of the roadmap in the context of the Delta variant;. Accessed: 2021-07-30. `https://assets.publishing.service.gov.uk/government/uploads/system/uploads/attachment_data/file/1001177/S1303_Imperial_College_London_Evaluating_the_Roadmap_out_of_Lockdown_for_England_modelling_the_delayed_step_4.2_of_the_roadmap_in_the_context_of_the_Delta_variant__7_July_2021__1_.pdf`.

[53] COVID-19 daily announced vaccinations 16 July 2021;. Accessed: 2021-07-20. `https://www.england.nhs.uk/statistics/statistical-work-areas/covid-19-vaccinations/covid-19-vaccinations-archive/`.

[54] FitzJohn R, Knock E, Whittles L, Perez-Guzman P, Bhatia S, Guntoro F, et al. Reproducible parallel inference and simulation of stochastic state space models using odin, dust, and mcstate [version 2; peer review: 1 approved, 1 approved with reservations]. Wellcome Open Research. 2021;5(288).

[55] R Core Team. R: A Language and Environment for Statistical Computing. Vienna, Austria; 2018. Available from: `https://www.R-project.org/`.

[56] Shapley LS. A Value for N-Person Games. Santa Monica, CA: RAND Corporation; 1952.
